# Nuclear blebs are associated with destabilized chromatin-packing domains

**DOI:** 10.1242/jcs.262161

**Published:** 2025-02-11

**Authors:** Emily M. Pujadas Liwag, Nicolas Acosta, Luay Matthew Almassalha, Yuanzhe (Patrick) Su, Ruyi Gong, Masato T. Kanemaki, Andrew D. Stephens, Vadim Backman

**Affiliations:** ^1^ Department of Biomedical Engineering, Northwestern University, Evanston, IL 60208, USA; ^2^ IBIS Interdisciplinary Biological Sciences Graduate Program, Northwestern University, Evanston, IL 60208, USA; ^3^ Center for Physical Genomics and Engineering, Northwestern University, Evanston, IL 60208, USA; ^4^ Department of Gastroenterology and Hepatology, Northwestern Memorial Hospital, Chicago, IL 60611, USA; ^5^ Department of Chromosome Science, National Institute of Genetics, ROIS, Mishima, Shizuoka 411-8540, Japan; ^6^ Graduate Institute for Advanced Studies, SOKENDAI, Mishima, Shizuoka 411-8540, Japan; ^7^ Department of Biological Science, The University of Tokyo, Tokyo 113-0033, Japan; ^8^ Biology Department, University of Massachusetts Amherst, Amherst, MA 01003; ^9^ Molecular and Cellular Biology, University of Massachusetts Amherst, Amherst, MA 01003, USA

**Keywords:** Chromatin motion, Chromatin nanodomains, Heterochromatin, Lamin, Nuclear blebbing, Nuclear mechanics, Nanoscale imaging

## Abstract

Disrupted nuclear shape is associated with multiple pathological processes including premature aging disorders, cancer-relevant chromosomal rearrangements and DNA damage. Nuclear blebs (i.e. herniations of the nuclear envelope) can be induced by (1) nuclear compression, (2) nuclear migration (e.g. cancer metastasis), (3) actin contraction, (4) lamin mutation or depletion, and (5) heterochromatin enzyme inhibition. Recent work has shown that chromatin transformation is a hallmark of bleb formation, but the transformation of higher-order structures in blebs is not well understood. As higher-order chromatin has been shown to assemble into nanoscopic packing domains, we investigated whether (1) packing domain organization is altered within nuclear blebs and (2) whether alteration in packing domain structure contributed to bleb formation. Using dual-partial wave spectroscopic microscopy, we show that chromatin-packing domains within blebs are transformed both by B-type lamin depletion and the inhibition of heterochromatin enzymes compared to what is seen in the nuclear body. Pairing these results with single-molecule localization microscopy of constitutive heterochromatin, we show fragmentation of nanoscopic heterochromatin domains within bleb domains. Overall, these findings indicate that chromatin within blebs is associated with a fragmented higher-order chromatin structure.

## Teaser

**Highlighted Article:** Nuclear blebs are linked to various pathologies, including cancer and premature aging disorders. We investigate alterations in higher-order chromatin structure within blebs, revealing fragmentation of nanoscopic heterochromatin domains.

## INTRODUCTION

The mammalian cell nucleus is a membrane-enclosed organelle that provides an enclosure for chromatin, the assembly of DNA and associated proteins that regulates crucial processes, such as gene transcription, replication and DNA repair. Chromatin, chromatin proteins and chromatin-related processes directly influence nuclear mechanics and shape (Berg et al., 2023; Chiu et al., 2024; Currey et al., 2022; Stephens et al., 2017, 2018). Nuclear stability is further maintained by multiple processes, including by the nuclear lamina, a meshwork of type V intermediate filament proteins called lamins (Funkhouser et al., 2013). Besides its role in maintaining nuclear stiffness and stability, the lamina plays crucial roles in regulating gene expression and DNA replication through chromatin interactions. Located immediately underneath the inner nuclear membrane, the lamina consists of four major types of lamin proteins: lamin A, lamin C, lamin B1 and lamin B2. A-type lamins, which consist of lamins A and C, are primarily associated with developmental roles, contribute to nuclear stiffness, mainly expressed in differentiated cells and are spatially located near the nucleoplasm (Berg et al., 2023; Chiu et al., 2024; Currey et al., 2022; Funkhouser et al., 2013; Nmezi et al., 2019; Stephens et al., 2017, 2018). B-type lamins, in contrast, are expressed in all cell types throughout development and differentiation, provide global integrity of chromatin structure through chromatin tethering and are tightly associated with the inner nuclear membrane (Burke and Stewart, 2013; Chang et al., 2022; Nmezi et al., 2019). In mammalian cells, lamins interact with heterochromatin to form lamina-associated domains (LADs), identified through the DamID technique which maps protein–DNA interactions in a genome-wide manner, and are typically transcriptionally repressive environments. Disruption of these LADs has been linked to epigenetic changes in cancer and pre-malignant processes, such as the onset and evasion of senescence (Lochs et al., 2019).

Abnormal nuclear morphology and disruption of genome organization are associated with pathologies, such as laminopathies (e.g. Hutchinson–Gilford progeria syndrome), cancer and cardiac disorders (Kalukula et al., 2022; Stephens et al., 2019a, 2018). Among the most radical deformations in nuclear shape is the protrusion of chromatin from the nuclear surface, known as a nuclear bleb, which is associated with pathological transformation (Funkhouser et al., 2013; Karoutas and Akhtar, 2021; Stephens et al., 2019a, 2018). Although these blebs are highly associated with gene-rich euchromatin and are believed to only contain lamin A/C (Nmezi et al., 2019), recent evidence has indicated that non-canonical blebs also contain B-type lamins (Bunner et al., 2025; Stephens et al., 2018). A- and B-type lamins both contribute to nuclear mechanics and morphology, and depletion of either has been widely shown to induce both abnormal nuclear shape and a higher propensity for nuclear rupture, an increased presence of micronuclei and more nuclear blebbing events (Kalukula et al., 2022; Karoutas and Akhtar, 2021; Vahabikashi et al., 2022). Nucleus micromanipulation force measurements reveal that the nucleus is softer upon inhibition of either histone deacetylation (i.e. histone deacetylases; HDACs) or histone methyltransferases (HMTs), which leads to nuclear bleb formation independently of lamins (Stephens et al., 2019a, 2018). Thus, chromatin and lamins resist external antagonistic forces from actin contraction (Jung-Garcia et al., 2023; Mistriotis et al., 2019; Nmezi et al., 2019; Pho et al., 2024) and compression (Hatch and Hetzer, 2016; Le Berre et al., 2012), as well as internal transcription forces (Berg et al., 2023), to maintain nuclear shape. These studies indicate that nuclear mechanics are influenced by the balance of euchromatin and heterochromatin, and that perturbation of this balance can result in abnormal nuclear morphology and DNA damage, both hallmarks of human disease (Evangelisti et al., 2022; Karoutas and Akhtar, 2021; Stephens et al., 2019a). At the nuclear periphery, the dynamics of cytoskeleton reorganization and chromatin structural changes contribute to mechanotransduction and transcription, independently of lamins. For example, mechanosensitive ion channels embedded in the plasma membrane activate Ca^2+^ signaling upon cellular stress, which can contribute to heterochromatin reorganization and chromatin mobility (Dupouy et al., 2024; Nava et al., 2020; Qin et al., 2021; Stephens et al., 2019a).

Recent work has demonstrated that chromatin assembles into higher-order polymeric domain structures (nanodomains, packing domains and chromatin cores), which range between 50–200 nm in size and contain ∼200 kbp to 2 Mbp of genomic content across multiple cell types (Li et al., 2022, 2021; Szabo et al., 2020). A crucial feature of these domains is the formation of high-density centers with surrounding regions of decreased density until a transition into low-density space with RNA-polymerase activity forming primarily at the boundary. In the context of these findings, the structure of the genome assembles from disordered nucleosomes (5 to 25 nm) transitioning into domains (50–150 nm) and then into territorial polymers (>200 nm). As has been previously shown, within the regime of chromatin assembling into domains, chromatin acts as a power-law polymer with dimension *D* relating how the mass is distributed within the occupied volume (Almassalha et al., 2025; Li et al., 2022). Notably, within supra-nucleosomal length scales, chromatin is not assembled purely as a space-fulling globule (*D*=3) nor is it a poorly structured polymer with monomers primarily favoring solvent interactions (*D*=5/3), instead it is typically within these ranges and varies from cell to cell. A key feature identified in this higher-order assembly is the coupling between heterochromatin centers (dense cores) with a corrugated euchromatic periphery (Li et al., 2021). As power-law polymeric assemblies, the space filling geometry of these chromatin-packing domains is quantifiable by the relationship *M* ∝ *r^D^*, relating how genome content fills an occupied volume as a function of its radial distance, *r.* This organization can be measured by live-cell dual-partial wave spectroscopic (dual-PWS) microscopy and quantified via the local average chromatin packing scaling *D*_a_*(x,y)*, and its reported ensemble average *D*_n_, which is proportional to the volume fraction that these chromatin-packing domains occupy within the nucleus and their space filling geometry (see Materials and Methods). In addition to quantifying the packing domain structure in live cells, PWS microscopy allows measurement of the effective diffusion coefficient, *D*_e_, and the fractional moving mass (FMM), which quantifies the fraction of chromatin demonstrating coherent motion within a diffraction limited volume. Utilizing this technique, we have previously demonstrated that B-type lamin depletion is associated with increased levels of chromatin fractional moving mass and repositioning of heterochromatin cores (Pujadas Liwag et al., 2024).

A major challenge in studying alteration in chromatin due to blebbing is that these represent infrequent, but crucial events in disrupting nuclear structure. As such, techniques such as Hi-C or ChIP-Seq, which capture collective events from millions of cells, might not reveal the disruption event and instead this requires the utilization of microscopic methods that can directly quantify changes in high-order genome structure. Therefore, in this study, we utilize live-cell PWS microscopy to investigate the interplay between the disruption of the nuclear lamina and heterochromatin enzymes in the structure of higher-order chromatin within blebs. Our results indicate distinct roles for the nuclear lamina and heterochromatin remodeling processes in regulating higher-order chromatin domains, both of which are associated with bleb formation. Finally, pairing our findings with super-resolution microscopy, we show that a key transformation of higher-order chromatin occurs within blebs of nanoscopic domains.

## RESULTS

### B-type lamin depletion or heterochromatin loss promotes aberrant nuclear morphology in HCT-116 cells

**Fig. 1.**
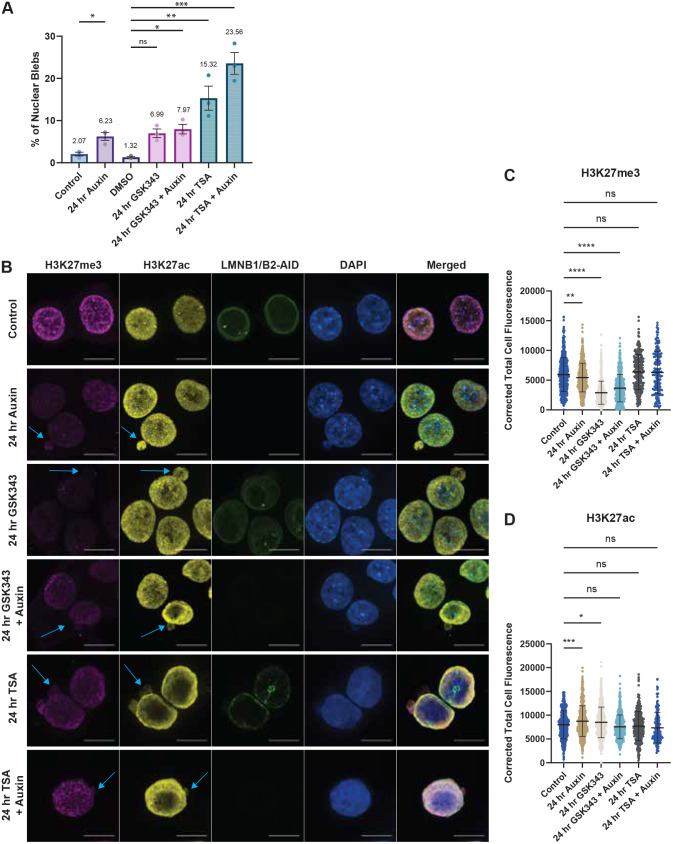
**Aberrant nuclear morphology is induced by the loss of B-type lamins or heterochromatin.** (A) Percentages of cells with nuclear blebs compiled over each field of view for untreated (control), 24-h auxin conditions in HCT116^LMN(B1&B2)-AID^ cells, DMSO, 24-h GSK343, 24-h TSA, 24-h GSK343 with auxin and 24-h TSA with auxin. Each dot represents a technical replicate (*N*=3; Control *n*=1102, auxin *n*=1081, DMSO *n*=295, GSK343 *n*=1096, TSA *n*=456, GSK343+auxin *n*=933, TSA+auxin *n*=456). Results shown as mean±s.e.m. (B) Representative confocal images of H3K27me3 (magenta), H3K27ac (yellow), lamin B1/B2-AID (green), DAPI (blue) and merged fluorescence for control, 24-h auxin, 24-h GSK343, 24-h GSK343+auxin, 24-h TSA, and 24-h TSA+auxin treatment conditions in HCT116^LMN(B1&B2)-AID^ cells. Scale bars: 10 µm. Blue arrows indicate nuclear bleb deformations. (C) Corrected total cell fluorescence measurements of H3K27me3 and (D) H3K27ac for control, 24-h auxin, 24-h GSK343, 24-h GSK343+auxin, 24-h TSA, and 24-h TSA+auxin treatment conditions in HCT116^LMN(B1&B2)-AID^ cells. Each dot represents a cell nucleus. Violin plots with the median and quartiles overlaid are shown. For C,D, data are representative of two technical replicates (*N*=2, total *n*>150 for each condition). *P≤0.05, **P≤0.01, ***P≤0.001, ****P≤0.0001; ns, not significant [unpaired two-tailed *t*-test with Holm–Šídák test for multiple comparisons (A); one-way ANOVA with Dunnett's test for multiple comparisons (C,D)].

Bleb formation has been identified in numerous cell types, but the frequencies of bleb formation have been shown to depend on multiple factors. Therefore, we first investigated the role of processes well established to induce bleb formation – inhibition of B-type lamins and disruption in heterochromatin enzymes (Stephens et al., 2018, 2019b). To assess the impact of lamin degradation on nuclear morphology, we applied the auxin-inducible degron (AID) system to HCT116 colorectal carcinoma epithelial cells to induce simultaneous degradation of lamin B1 and lamin B2 as previously described (Pujadas Liwag et al., 2024; Yesbolatova et al., 2019). This cell line has an mClover fluorescent reporter fused to the lamin B1 and B2 proteins that were used in our degradation experiments. Using immunofluorescence imaging, we quantified the percentages of nuclear blebbing in HCT116^LMN(B1&B2)-AID^ cells before and after depletion of B-type lamins by auxin treatment for 24 h. We found that in comparison to untreated controls, auxin treatment promoted a significant increase in the percentage of cells containing nuclear blebs (2.07% versus 6.23%; 4.163±1.033, mean±s.e.m., *P*-value=0.016, two-tailed unpaired Student's *t*-test) (Fig. 1A, Table S1), in agreement with past studies (Chen et al., 2018; Stephens et al., 2018; Vargas et al., 2012). Previous work from this group has demonstrated that inhibition of HDACs to increase euchromatin content in mammalian cells or inhibition of HMTs to decrease heterochromatin content results in a softer nucleus and promotes nuclear blebbing, without perturbing lamins (Stephens et al., 2019a, 2018). We therefore hypothesized that in addition to B-type lamin loss increasing nuclear blebbing, heterochromatin loss would also result in a substantial increase in nuclear blebs in HCT-116 cells. To test this, we treated HCT116^LMN(B1&B2)-AID^ cells with either GSK343, an inhibitor of the histone methyltransferase enhancer of zeste homolog 2 (EZH2) or trichostatin A (TSA), an inhibitor of class I and II HDACs for 24 h.

Our results confirmed that GSK343 and TSA treatment significantly increased the percentage of blebbed nuclei within HCT-116 cells; TSA treatment also induced micronuclei formation and was associated with a deformed nuclear periphery because of an increased number of blebs (Fig. 1A; Table S1). The effect of TSA treatment on nuclear blebbing frequency was drastically higher than that of auxin or GSK343 in HCT116^LMN(B1&B2)-AID^ cells (auxin 6.23%, GSK343 6.99% and TSA 15.32%). Next, we explored how combined treatment of either auxin and GSK343 or auxin and TSA would impact the frequency of nuclear blebs. Combination of depletion of B-type lamins with disruption of heterochromatin resulted in only slightly increased rates of nuclear blebs in comparison to what was seen with lamin depletion, GSK343 or TSA treatment alone (no significant difference for auxin+GSK343 at 7.97% and auxin+TSA at 23.56%) (Fig. 1A). We found no significant difference between cells treated with or without auxin in both TSA- and GSK343- treated groups (TSA versus TSA+auxin, *P*=0.078, and GSK343 vs GSK343+auxin, *P*=0.8235).

Using immunofluorescence microscopy, we visualized these blebs and compared relative levels of H3K27me3 and H3K27ac between the untreated control and auxin-, GSK343- and TSA-treated conditions within the cell nucleus (Fig. 1B). Consistent with prior reports, we observed a reduction in H3K27me3 levels upon auxin-induced degradation of B-type lamins and GSK343 treatment respectively. For H3K27ac, we observe an increase in levels in the auxin-treatment group and GSK343 treatment respectively (Fig. 1C,D). Taken together, these results indicated that TSA-mediated heterochromatin disruption promotes nuclear blebbing to similar levels or greater levels than B-type lamin loss in HCT-116 cells.

### Nanoscale chromatin-packing domains are disrupted within nuclear blebs

We have recently demonstrated that the decreased DNA density is conserved across multiple bleb mechanisms and is a consistently preserved feature of blebs (Bunner et al., 2025). We investigate here in greater detail the influence in the change of higher-order chromatin organization upon bleb formation. While it is commonly assumed that heterochromatin is primarily localized to the nuclear periphery and peri-nucleolar space, recent work with super resolution imaging indicates high-density heterochromatin throughout the nuclear interior. We have previously shown that live-cell PWS microscopy, which does not resolve each individual domain but measures the local ensemble in individual nuclei (see Materials and Methods), is sensitive to detecting the assembly into supra-nucleosome structures in individual cells with measurements comparable to those observed on electron microscopy by measuring the variations in the visible-light interference spectrum from within the nucleus (Almassalha Luay et al., 2016; Gladstein et al., 2019, 2018; Li et al., 2022, 2021). The relationship between genomic length (*N*) and radius (*r*) can be used to quantify how chromatin is packed within a given volume. This relationship is characterized by the mass fractal dimension (*D*), which describes how densely the chromatin mass fills the space it occupies. In principle, mass scaling can be quantified at various levels of genome organization, including an entire chromosome or the genomic scaling within individual chromatin-packing domains (Li et al., 2022). We focus on supra-nucleosomal domain length-scales (50–150 nm) within this work due to recent observations demonstrating that this regime is paired with crucial functions, such as gene transcription (Li et al., 2021; Miron et al., 2020). PWS enables the measurement of local average chromatin packing scaling (*D*_a_) at specific (*x*,*y*) locations within the nucleus with sensitivity primarily to structures within the length-scales of chromatin domains (50–200 nm). *D*_a_(*x,y*) is proportional to the average packing scaling within the coherence volume probed. The nuclear average scaling (*D*_n_), obtained by averaging *D*_a_(*x*,*y*) across the nucleus, is proportional to the volume fraction of chromatin domains within the nucleus and their scaling *D*. As chromatin is organized in mass-fractal packing domains, *D*_n_ measures the likelihood of chromatin organizing into domains with the ensemble scaling. Likewise, we have shown that by analyzing the temporal interference spectrum at a single-wavelength, dual-PWS microscopy can measure the temporal evolution of chromatin density and the fractional moving mass (FMM), which measures the volume fraction of- and mass of- chromatin moving coherently with a sensitivity to mass density fluctuations as low as ∼5×10^−21^ g, and the effective diffusion coefficient (*D*_e_) within the nucleus (ranging between ∼0.065 µm^2^/s and 3.5×10^−5^ µm^2^/s) (Gladstein et al., 2019). In the context that the mass of an individual nucleosome of ∼10^−19^ g, the typical values of FMM measured represent the movement of nucleosome clutches moving coherently (as an ensemble). With respect to the *D*_e_, the observed values are typically between the observed rate of diffusion for genomic loci (∼10^−4^ µm^2^/s) and the rate of mRNA through the nucleus (∼5×10^−2^ µm^2^/s) (Li et al., 2021). Given these considerations, we utilized dual-live cell PWS microscopy to probe the higher-order structure of chromatin in blebs, nuclei with blebs and stable nuclei.

**Fig. 2.**
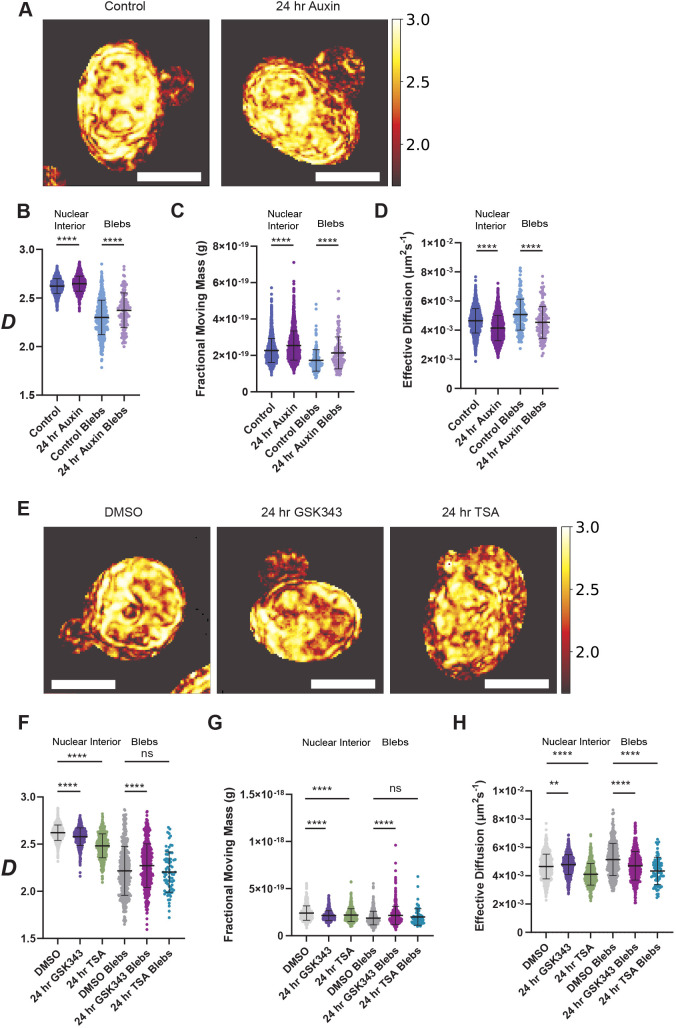
**Loss of B-type lamins, EZH2 inhibition and HDAC inhibition induce a bleb-associated chromatin phenotype.** (A) Representative PWS *D* maps and (B) *D* values for the nuclear bodies and nuclear blebs for control and 24-h auxin-treatment conditions in HCT116^LMN(B1&B2)-AID^ cells. (C) FMM values for nuclear bodies and nuclear blebs for control and 24-h auxin-treatment conditions in HCT116^LMN(B1&B2)-AID^ cells. (D) Effective diffusion coefficient values for the nuclear bodies and nuclear blebs for control and 24-h auxin-treatment conditions in HCT116^LMN(B1&B2)-AID^ cells. (E,F) Representative PWS *D* maps (E) and values (F) for the nuclear bodies and nuclear blebs for DMSO (vehicle control), 24-h GSK343 (EZH2 inhibition) and 24-h TSA-treatment (HDAC inhibition) conditions in HCT116^LMN(B1&B2)-AID^ cells. (G) FMM values for the nuclear bodies and nuclear blebs for DMSO (vehicle control), 24-h GSK343, and 24-h TSA-treatment conditions in HCT116^LMN(B1&B2)-AID^ cells. (H) Effective diffusion coefficient values for the nuclear bodies and nuclear blebs for DMSO (vehicle control), 24-h GSK343 and 24-h TSA-treatment conditions in HCT116^LMN(B1&B2)-AID^ cells. For B–D, F–H, each dot represents a cell nucleus (control *n*=2451, auxin *n*=2140, Control Blebs *n*=200, auxin Blebs *n*=129, DMSO *n*=741, GSK343 *n*=790, TSA *n*=498, DMSO Blebs *n*=564, GSK343 Blebs *n*=467, TSA Blebs *n*=77). Data are compiled from three technical replicates (*N*=3). Violin plots with the median and quartiles overlaid are shown. **P≤0.01; ****P≤0.0001; ns, not significant (unpaired two-tailed *t*-test between selected groups). Scale bars: 5 µm.

Applying dual-PWS to the three well-known processes that contribute to bleb formation, we investigated the structure of higher-order chromatin and mobility after B-type lamin depletion, HDAC inhibition (TSA) and in EZH2 inhibition (GSK343). As each perturbation has a distinct means to promote bleb formation, we first evaluated the structure of chromatin-packing domains observed within blebs in all conditions (controls, GSK343, TSA and lamin depletion) to see whether any commonalities were present (Table 1; Fig. 2A–F). Overall, this indicates that independently of the mechanism of bleb formation, higher-order chromatin organization within blebs is associated with a lower-likelihood of well-formed packed domains (low *D*) and fragmented clutches (decreased FMM) with increased mobility (*D*_e_) compared to what is seen in the nuclear body. Comparing the observed behavior of chromatin domains across the nuclei in these conditions, we observed that inhibition of with GSK343 or TSA resulted in decreased *D* and FMM (Fig. 2F,G) in comparison to what is seen in untreated controls.

**Table 1.** Dual-PWS measurements of nuclear blebs

Condition	Average *D*	Average FMM (g)	Average *D_e_* (µm^2^/s)
Control (untreated)	2.30	1.76×10^−19^	5.09×10^−3^
Control (DMSO)	2.22	1.89×10^−19^	5.12×10^−3^
(−) Lamin B1/B2	2.37 (±0.03)	2.26×10^−19^ (±	4.35×10^−3^ (±0.0003)
(−) EZH2	2.27 (±0.03)	2.16×10^−19^ (±1.53×10^−20^)	4.71×10^−3^ (±0.0001)
(−) HDACs	2.20 (±0.03)	1.99×10^−19^ (±1.53×10^−20^)	4.34×10^−3^ (±0.0001)

All results are mean±s.e.m. Control untreated, *n*=564 blebs in three replicates; control DMSO, *n*=458 in three replicates; lamin B1 and B2, *n*=129 in three replicates; EZH2, *n*=466 in three replicates; TSA, *n*=77 in three replicates.

Strikingly, even though the higher-order chromatin structure within blebs was similar in all states compared to the nuclear body, the transformation of chromatin within the body was distinct for each mechanism. Specifically, B-type lamin depletion was associated with an increase in *D* and FMM compared to control cells (Fig. 2B,C) whereas inhibition of heterochromatin enzymes resulted in low *D* compared to their respective controls (Fig. 2F,G). This indicates that disruption of heterochromatin enzymatic processes produces chromatin domain fragmentation that is not occurring with B-type lamin depletion. Next, we compared the behavior of chromatin domains within blebs across all conditions. We unexpectedly observed that domains caused by B-type lamin depletion had a comparably higher *D* and FMM compared to those that occur caused by inhibition of heterochromatin remodeling enzymes (Fig. 2B,C,F,G; Fig. S1A,B). When paired with the findings in the nuclear body of each respective perturbation, this suggests a potential differential mechanism of bleb translocation for each model. Specifically, heterochromatin inhibition might be producing smaller domains that are spontaneously passing through a smaller initial deformation of the nucleus. By contrast, the disruption of the lamina might be resulting in larger membrane defects that facilitate the passage of otherwise stable domains.

**Fig. 3.**
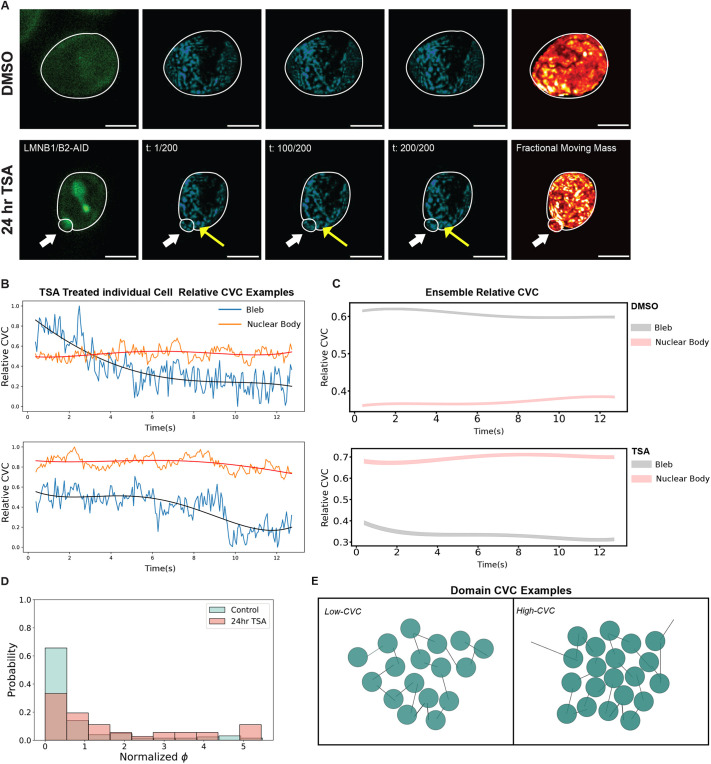
**Chromatin density decreases rapidly in the boundary adjacent to the nuclear bleb.** (A) Representative fluorescent mClover signal images, FMM ‘hot’ heat map, and individual frames of the temporal interference signal, inversely proportional to chromatin density over the imaging acquisition time of live HCT116^LMN(B1&B2)-AID^ cells (non-degraded) for DMSO (control) and 24-h TSA treatment all acquired via the Dual-PWS system. White arrows indicate nuclear blebs and yellow arrows indicate areas of interest of motion. Data are representative of three technical replicates (*N*=3; DMSO *n*=741, TSA *n*=498). Fluorescence, FMM and individual frame images have the same scaling between treatment conditions. Scale bars: 5 µm. (B) Representative relative CVC change over time for two regions of interest in a single cell treated with TSA within the dataset. Blue and orange curves represent the Relative CVC for blebs and nuclear body respectively. Both colors show the original dynamic PWS intensity as a function of time, with high frequency components measuring the FMM and the low frequency components records the average CVC change. A fourth order polynomial is fit to the raw signal as shown by the red and black curves following the body and bleb signal respectively. (C) An ensemble relative CVC for all nuclei in DMSO (top) and TSA (bottom)-treated conditions over time. Line and shaded region represent average fourth order polynomial fit with standard error. (D) Bleb average CVC for DMSO (control, 172 bleb, 308 body) and 24-h TSA treatment (treated, 40 bleb, 217 body) measured from dynamics PWS signal for nuclear body and bleb regions. Data are the same as in A. (E) Shows bleb-to-body ratio for relative CVC measurements for CTRL (98 pairs) and SA (31 pairs) treatment conditions. (F) Schematic illustrating the differences between low and high CVC domains.

To further characterize the temporal dynamics of nuclear blebbing induced by either B-type lamin loss or heterochromatin disruption, we used dual-PWS to measure the mobility of mass between the nuclear bleb and nuclear body in live cells. As TSA treatment resulted in the most substantial increase in the frequency of nuclear blebs in the experiments above, we treated HCT116^LMN(B1&B2)-AID^ cells with either DMSO or TSA. By measuring the spectral interference at a single wavelength as a function of time from within nuclei and within blebs, we could directly image and measure how mass was transitioning between these spaces (Gladstein et al., 2019). An advantage of this approach is that it can account for the transfer of both chromatin and non-chromatin proteins which would otherwise be challenging to quantify concurrently. As discussed above, variations in the temporal interference quantifies the FMM whereas the temporal-average signal is inversely proportional to the chromatin volume concentration (chromatin density) and the density of other nuclear macromolecules (Eid et al., 2020; Gladstein et al., 2019). Chromatin volume concentration (CVC), as the name implies, describes how chromatin occupies the volume on a relative scale of 0 to 1. Two examples of relative CVC that are seen in our analysis are illustrated in Fig. 3F.

Utilizing this approach, we can visualize the temporal evolution of density in both the nucleus and the bleb with a very high temporal resolution of 50 ms per frame (acquired over 15 s total in this instance). On imaging chromatin motion in the DMSO control nuclei, it is visually apparent that density moves randomly within the nucleus at short timescales (Movies 1–3). In contrast, within a TSA-treated cell with a visually apparent bleb, density decreases rapidly adjacent to the nuclear bleb (Fig. 3A,B; Movies 1–3). In the TSA-treated case (Fig. 3A, bottom), we observed transit of small amounts of mass between the nuclear bleb and the main nuclear body. This is evidenced by an increased local density and the temporally evolving density in the bleb (Fig. 3A, from TSA t:1/200 to t:200/200). When measuring the relative CVC change over time in individual cells, we see that there is a heterogeneity when looking at both nuclear body and bleb regions of interest (Fig. 3B). Interestingly, in the population ensemble (mean trendline fits±s.e.m.), we observe a higher relatively higher CVC for the nuclear body in the TSA-treated case compared to that for the accompanying blebs (Fig. 3C, bottom).

In contrast, blebs occurring in control cells which showed the opposite trend from TSA-treated cells, with higher local density relative to the adjacent nucleus. In the context that DNA density is decreased in blebs relative to the body (Bunner et al., 2025) and the disruption of domains as quantified by the observed *D* within blebs, this indicates that blebs might become enriched in other non-chromatin macromolecules. When looking at regional chromatin concentration for bleb and nuclear regions for DMSO control and TSA, we observed an increase in density for both the bleb and nuclear body in the TSA-treated cells compared to that in the control (Fig. 3D). Given that *D* decreases throughout the nucleus in TSA-treated cells, this finding suggests that unexpectedly, even as chromatin domains are destabilized, nuclear density might be increasing from the presence of other macromolecules. Directly looking at the CVC for the bleb-to-nuclear body ratio for paired nuclei, we see that there is a lower bleb-to-body ratio for the TSA-treated case. Collectively, these findings demonstrate a destabilization of chromatin domains within blebs with a concurrent accumulation in non-chromatin macromolecules.

### Super-resolution imaging of chromatin heterochromatin clusters in nuclear blebs

**Fig. 4.**
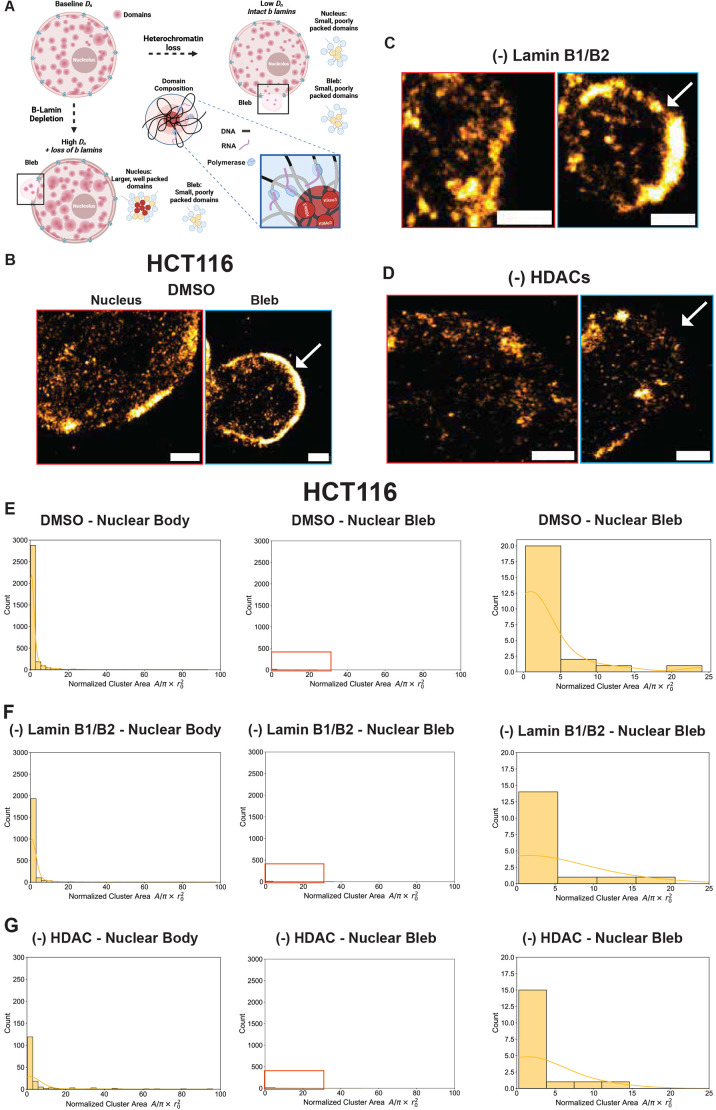
**Characterization of heterochromatin nanodomains from SMLM images in HCT116 cells.** (A) Schematic of nucleus illustrating heterochromatin nanodomains. Change in D and inhibition of heterochromatin (HDACi) causes domain collapse and fragmentation in the blebs. While treatment with auxin leads to loss of b type lamin and higher Dn. As highlighted, the nuclear blebs contain small domain cores. Created in BioRender by Almassalha, L., 2025. https://BioRender.com/l20m056. This figure was sublicensed under CC-BY 4.0 terms. (B–D) Representative SMLM images of HCT116^LMN(B1&B2)-AID^ cells with magnified views (B) before (DMSO, 300 nM) and after (C) 24-h auxin treatment or (D) 24-h TSA treatment (300 nM). Whole nucleus images have red borders and nuclear bleb views have a blue border. Yellow: H3K9me3. White arrows placed to highlight presence of H3K9me3 signal in the nuclear blebs. Scale bars: 1 µm. (E–G) Quantification of the number and size of heterochromatin nanodomains in control, auxin and TSA treatment conditions for HCT116 cells within nuclear body and nuclear bleb formation. Data presented in a 3×3 panel, where the first panel is the count for the nuclear body, the middle panel is the nuclear bleb with the same scale used in the and the third panel is the same data, but with adjusted scales so the histogram count is visible. HCT116 DMSO, *n*=12; HCT116 TSA, *n*=10; U2OS DMSO, *n*=15; U2OS TSA, *n*=7.

**Fig. 5.**
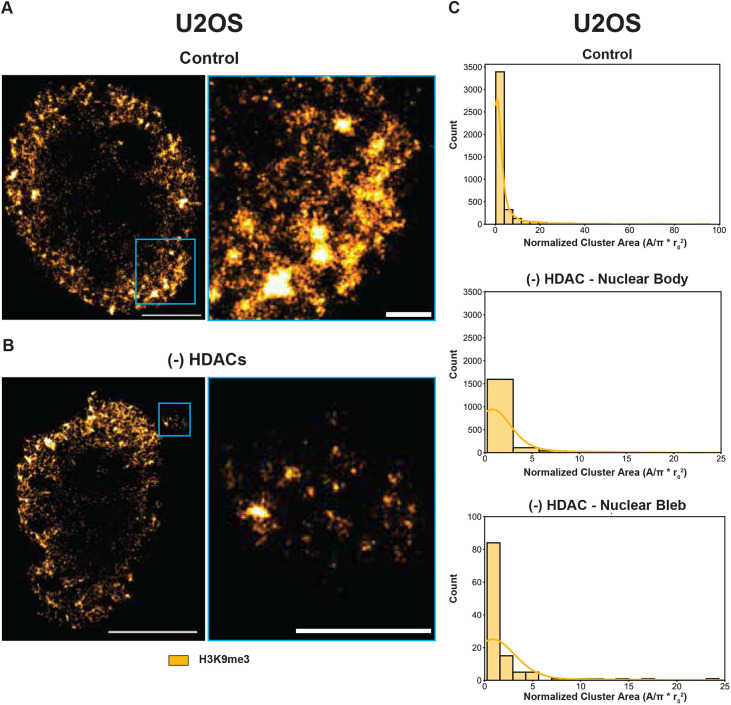
**Heterochromatic nanodomains are reorganized during nuclear bleb formation.** (A,B) Representative SMLM images of U2OS cells with magnified views (A) before and (B) 24-h TSA treatment. Yellow, H3K9me3. Data are representative of three technical replicates [*N*=3; Control *n*=3, TSA (HDAC inhibition) *n*=3]. Scale bars: 5 µm (whole nucleus), 1 µm (magnified nuclear bleb, blue border). (C) Quantification of the number and size of heterochromatin nanodomains in control and TSA treatment conditions for U2OS cells.

Nuclear lamins and heterochromatin have been shown to act in parallel to maintain the mechanical properties of the nucleus but the consequence of these on chromatin nanodomains in bleb formation have not been investigated (Hoskins et al., 2021; Nava et al., 2020; Stephens et al., 2019b). Additionally, chromatin structure and dynamics are often closely related, which may support mechanisms of either granting or limiting access to regions with high local chromatin concentration (Barth et al., 2020). In the context of prior work suggesting that chromatin domains are composed of high-density, presumably heterochromatic centers (Li et al., 2022, 2021; Miron et al., 2020; Szabo et al., 2020), we investigated the transformation in constitutive heterochromatin domains between the nuclear bleb and the nuclear body in spontaneously forming blebs (controls), in lamin B1 and B2 depletion-associated blebs, and in heterochromatin enzyme-inhibited blebs (TSA) using single-molecule localization microscopy (SMLM). SMLM is a super-resolution imaging technique that allows for the precise localization of individual fluorescent molecules, achieving spatial resolutions beyond the diffraction limit of light. This method enables detailed visualization of molecular structures and interactions within cells, providing insights into biological processes at the nanoscale. A specific version of this technique, stochastic optical reconstruction microscopy (STORM), was used here to image chromatin, specifically allowing us to differentiate between distinct structures within a diffraction limited volume or the ensemble behavior of mass. In this paper, we chose to image the positioning of H3K9me3 as it is associated with high-density regions that are observed to form the centroids of domain cores on electron microscopy (Miron et al., 2020; Almassalha et al., 2025). By pairing this with dual-PWS microscopy, we can investigate both the ensemble behavior of chromatin throughout the nucleus and the nanoscopic structure of high-density regions formed by heterochromatin. This is achieved with its high resolution capable of resolving nanoscopic structures between 10 and 30 nm. Owing to the limitation of bleb formation being a low-frequency process (<15% of the time) (Table S1), we were only able to identify blebs in a few nuclei in total in HCT-116 cells (Fig. 4B–D). Given this limitation, we utilized a second cell line model, U2OS cells, which are associated with higher rates of bleb formation upon HDAC inhibition with TSA than HCT-116 cells (Fig. 5).

Visually, we observed distinct differences in H3K9me3 chromatin nanodomains (Fig. 4A) in these three conditions. In blebs formed spontaneously (Fig. 4B), blebs formed in B-type lamin depletion (Fig. 4C) and blebs formed due to inhibition of HDACs (Fig. 4D), it is visually apparent that nanoscopic heterochromatin domains are observed. Within the nuclear body, as previously demonstrated (Pujadas Liwag et al., 2024), auxin-induced depletion of B-type lamins resulted in reduced peripheral heterochromatic cores at the nuclear periphery, however, domains formed within the nuclear interior were typically larger in size (Fig. 4E–G). With respect to heterochromatin nanodomains in GSK343-treated HCT116 cells (Pujadas Liwag et al., 2024) and TSA-treated U2OS cells, these were smaller than those in control cells and in lamin B-depletion as expected due to the inhibition of heterochromatin enzymes within the nuclear body (Fig. 4E–G; Fig. 5C). In contrast to prior work, we found that the nuclear blebs arising from either B-type lamin degradation or TSA treatment contained heterochromatin around the periphery of blebs and within the center of the bleb (Fig. 4B–D; Fig. 5A,B). This finding highly contradicts the plethora of research stating that all nuclear blebs are devoid of heterochromatin (Bercht Pfleghaar et al., 2015; Helfand et al., 2012; Lammerding et al., 2006). Instead, these results show that independent from B-type lamin loss, TSA treatment gives rise to non-canonical nuclear blebs enriched in heterochromatin around near their boundaries and the transfer of nanoscopic heterochromatin domains into the bleb. This also challenges the notion that euchromatin enrichment is the most reliable marker of nuclear blebs (Bunner et al., 2025), and further suggests that other cellular mechanisms could play a role in the morphological properties of these herniations.

## DISCUSSION

In this work, we found that nuclear packing domains are transformed within blebs induced by the loss of either B-type lamins or inhibition of heterochromatin enzymes. Specifically, the domains observed within blebs were typically poorly formed, with increased fragmentation and a higher effective diffusion coefficient compared to the domains observed in the nuclear body in a manner that was independent of the conditions. Despite the similarities across groups, we saw that domains associated with lamin B depletion were likely larger than those produced by inhibition of heterochromatin remodeling enzymes (GSK343 inhibition of EZH2 and TSA inhibition of HDACs) suggesting that the barrier to movement of domains or nucleosome clutches is larger in the loss of B-type lamins whereas the inhibition of heterochromatin enzymes fragments domains to facilitate blebbing in the nuclear border (Fig. 2A,E). Given these findings, one possible and interesting explanation is that the size of domains influences their likelihood to transit through temporally evolving defects in the nuclear envelope. In the absence of B-type lamins, such defects are potentially more likely to have a larger cavity, facilitating the transit of domains that would be too large to transfer in control cells. As such, depletion of B-type lamins might increase the frequency of large barrier disruption events or potentially result in larger transient defects that allow passage of larger domains into the bleb body. Further supporting these findings is the observation that the heterochromatin clusters structures upon B-type lamin depletion are larger in size compared to those in nuclei treated with heterochromatin enzyme inhibitors as seen by super-resolution microscopy (Fig. 4F,G) within the nuclear body. Likewise, although limited by the low frequency of bleb events, domains observed within all blebs were smaller in size and more disperse than those observed in the adjacent nuclear body, indicating that domain size is a potential consideration in the likelihood of bleb formation (Figs 4,5).

The changes observed in chromatin domains within blebs could be related to functional consequences in signaling, possibly arising from applied mechanical stress when the nuclear lamina or heterochromatin are disrupted. For example, using single-nucleus isolation and micromanipulation assays, we have previously demonstrated that nuclei with reduced heterochromatin levels are softer and succumb to nuclear blebbing, whereas nuclei with more heterochromatin levels are stiffer and resist blebbing (Stephens et al., 2018). This chromatin histone-modification-based nuclear rigidity could be related to the differential transcriptional responsiveness (i.e. transcriptional plasticity) previously observed in low- versus high-chromatin packing areas upon exposure to external stressors (Virk et al., 2020). In many cases, nuclear blebbing is a marker of cell death (i.e. apoptosis) and is often observed during normal developmental processes or in response to various extracellular stressors. The transformation of domains within blebs upon either B-type lamin or heterochromatin enzyme disruption could potentially accelerate these processes by increasing DNA damage or cytoskeletal reorganization. Alternatively, the observation that density across the nucleus increases even as domain structure fragments indicates that pressure might accumulate from the mobilization of non-chromatin bound macromolecules. Bleb formation could in this case be a necessary event to maintain the stability of the remaining chromatin domains to ensure their continued cellular function (Fig. 3).

Although loss of B-type lamins and inhibition of heterochromatin enzymes both induced nuclear morphological changes and increased FMM within nuclear blebs, it is important to consider that these perturbations may not always reflect the same changes in cell phenotype. Although B-type lamins are required for proper spatial positioning of heterochromatin and gene-specific loci (Chang et al., 2022; Pujadas Liwag et al., 2024), B-type lamin loss and heterochromatin disruption may impact different cellular mechanisms that give rise to these morphological changes. For example, in previous work, we found that decreasing heterochromatin promoted decreased nuclear rigidity and increased nuclear blebbing without necessarily altering lamins (Stephens et al., 2018). Conversely, removal of B-type lamins resulted in both a reduction of heterochromatin and increased nuclear blebbing (Chang et al., 2022; Pujadas Liwag et al., 2024). Therefore, although lamins and heterochromatin interact, depletion of either could have differential effects on chromatin organization. In this work, we also found that TSA or GSK343 treatment promoted more nuclear blebs in comparison to auxin treatment to remove B-type lamins. Although combined treatment of either auxin and TSA or auxin and GSK343 did not result in a statistically significant increase in these bleb frequency in comparison to TSA or GSK343 alone, our results were similar to previous findings that conclude disruption of chromatin alone is sufficient to cause nuclear blebbing (Stephens et al., 2018). However, the distortions in the nucleus caused in part by the breakdown of connections between chromatin and the nuclear lamina might be intensified by pressure gradients resulting from external influences (Cao et al., 2016).

These external factors, such as confinement imposed by the actin cytoskeleton or the surrounding environment, could further contribute to the increased deformation of the nucleus. Additionally, processes such as inhibition of HDACs or HMTs could act by expanding the volume of heterochromatin centers or destabilizing packing domains altogether. In theory, as weak unstable packing domain (i.e. nascent domain) cores expand in size, one possible consequence could be increased variations in temporally active processes, such as gene transcription, resulting in amplified chromatin motion. Consequently, modifications to higher-order chromatin assemblies could promote bleb formation by degrading packing domains and/or altering chromatin-based nuclear mechanics. However, further assessment is needed to confirm this theoretical interplay between packing domain formation, nuclear mechanics and transcription.

The complexity of interactions within the genome results in varying chromatin dynamics at different length scales. Intrinsic characteristics of chromatin, which involve the dynamic rearrangement of histones, interactions among chromosome segments, chromatin remodelers, replication proteins and transcriptional regulators are required for proper spatiotemporal genome organization. Other than dual-PWS, several techniques have been utilized to investigate the contributions of chromatin dynamics to this organization. For example, a combination of photoactivated localization microscopy (PALM) and tracking of single nucleosomes has been recently applied to assess the role of nucleosome–nucleosome interactions and cohesion–RAD21 in domain formation and dynamics (Nozaki et al., 2017). In line with our results, TSA treatment increased chromatin dynamics. Recently, proximity ligation-based chromatin assembly assays have been applied to investigate the kinetics of nuclear lamina binding to newly replicated DNA in MEFs (Lovejoy et al., 2023 preprint). Finally, computational models have been applied to probe the time evolution of the chromatin over the G1 phase of the interphase in *Drosophila* that successfully predict dynamic positioning of all LADs at the nuclear envelope (Tolokh et al., 2023). Although chromatin scanning transmission electron microscopy (ChromSTEM) does not have live-cell imaging capabilities to resolve chromatin mobility (Li et al., 2022), future work might involve this high-resolution imaging technique to investigate how the shift in chromatin dynamics seen here could be related to shifts in chromatin density, volume and shape.

## MATERIALS AND METHODS

### HCT116 cell culture

HCT116^LMN(B1&B2)-AID^ cells and U2OS cells (ATCC) were grown in McCoy's 5A modified medium (#16600-082, Thermo Fisher Scientific, Waltham, MA, USA) supplemented with 10% FBS (#16000-044, Thermo Fisher Scientific) and 100 μg/ml penicillin-streptomycin (#15140-122, Thermo Fisher Scientific) (hereafter denoted complete cell medium). To create HCT116^LMN(B1&B2)-AID^ cells, HCT116 cells (ATCC, #CCL-247) were tagged with the AID system as previously described (Pujadas Liwag et al., 2024). All cells were cultured under recommended conditions at 37°C and 5% CO_2_. All cells in this study were maintained between passage 5 and 20. Cells were allowed at least 24 h to re-adhere and recover from trypsin-induced detachment. All imaging was performed when the surface confluence of the dish was at 40–70%. All cells were tested for mycoplasma contamination (ATCC, #30-1012K) before starting perturbation experiments, and they have given negative results.

### Auxin treatment

HCT116^LMN(B1&B2)-AID^ cells were plated at 50,000 cells per well of a six-well plate (Cellvis, P12-1.5H-N). To induce expression of OsTIR1, 2 μg/ml doxycycline (Thermo Fisher Scientific, #10592-13-9) was added to cells 24 h prior to auxin treatment. 1000 μM indole-3-acetic acid sodium salt (IAA, Sigma Aldrich, #6505-45-9) was solubilized in RNase-free water (Thermo Fisher Scientific, #10-977-015) before each treatment as a fresh solution and added to HCT116^LMN(B1&B2)-AID^ cells.

### GSK343 treatment

HCT116^LMN(B1&B2)-AID^ cells were plated at 50,000 cells per well of a six-well plate (Cellvis, P12-1.5H-N). Cells were given at least 24 h to re-adhere before treatment. GSK343 (Millipore Sigma, #SML0766) was dissolved in DMSO to create a 10 mM stock solution. This was further diluted in complete cell medium to a final treatment concentration of 10 µM. An equivalent final concentration of DMSO in medium was used for the control group such that DMSO was diluted in medium and added to cells in a 0.1% (v/v) ratio.

### Trichostatin A treatment

HCT116^LMN(B1&B2)-AID^ cells were plated at 50,000 cells per well of a 6-well plate (Cellvis, P12-1.5H-N). Cells were given at least 24 h to re-adhere before treatment. TSA (Millipore Sigma, #T1952) was diluted in complete cell medium and added to cells at a final treatment concentration of 300 nM. A final concentration of 0.1% (v/v) of DMSO was used as a control for this drug treatment.

### Immunofluorescence sample preparation

HCT116^LMN(B1&B2)-AID^ cells at a low passage (less than ten) were plated at 100,000 cells per well of a six-well glass-bottom plate (Cellvis, #P06-1.5H-N). Following auxin treatment, cells were washed twice with 1× phosphate buffered saline (PBS; Gibco, #10010031). Cells were fixed with 4% paraformaldehyde (PFA) (Electron Microscopy Sciences, #15710) for 10 min at room temperature, followed by washing with PBS three times for 5 min each. Cells were permeabilized using 0.2% Triton X-100 (10%) (Sigma-Aldrich, #93443) in 1× PBS, followed by another wash with 1× PBS for three times for 5 min each. Cells were blocked using 3% bovine serum albumin (BSA; Sigma-Aldrich, #A7906) in 1× PBS 0.2% (v/v) Tween-20 in (PBST; Sigma-Aldrich, #P9416) at room temperature. The following primary antibodies were added overnight at 4°C: anti-H3K27ac (Abcam, #ab177178, dilution 1:7000) and anti-H3K27me3 (Abcam, #ab6002, dilution 1:200). Cells were washed with 1× PBS three times for 5 min each. The following secondary antibodies were added for 1 h at room temperature: goat anti-rabbit IgG (H+L) conjugated to Alexa Fluor 568 (Abcam, #ab175471, dilution 1:1000) and goat anti-mouse IgG (H+L) highly cross-adsorbed secondary antibody conjugated to Alexa Fluor Plus 647 (Thermo Fisher Scientific, #A32728, dilution 1:200). Cells were washed with 1× PBS three times for 5 min each. Finally, cells were stained with DAPI (Thermo Fisher Scientific, #62248, diluted to 0.5 μg/ml in 1× PBS) for 10 min at room temperature. Prior to imaging, cells were washed with 1× PBS twice for 5 min each.

### Immunofluorescence imaging

Live and fixed cells were imaged using the Nikon SoRa Spinning Disk confocal microscope equipped with a Hamamatsu ORCA-Fusion Digital CMOS camera. Live cells were imaged under physiological conditions (37°C and 5% CO_2_) using a stage top incubator (Tokai Hit). Images were collected using a 60×/1.42 NA oil-immersion objective mounted with a 2.8× magnifier. mClover was excited with a 488 nm laser, Alexa Fluor 647 was excited with a 640 nm laser, and DAPI was excited with a 405 nm laser. Imaging data were acquired by Nikon acquisition software.

### Dual-PWS imaging

For live-cell measurements, cells were imaged and maintained under physiological conditions (5% CO_2_ and 37°C) using a stage-top incubator (In Vivo Scientific, Salem, SC; Stage Top Systems). Live-cell PWS measurements were obtained using a commercial inverted microscope (Leica, DMIRB) using a Hamamatsu Image-EM charge-coupled device (CCD) camera (C9100-13) coupled to a liquid crystal tunable filter (LCTF, CRi VariSpec) to acquire monochromatic spectrally resolved images ranging from 500–700 nm at 2-nm intervals as previously described (Almassalha Luay et al., 2016; Gladstein et al., 2019, 2018). Broadband illumination was provided by a broad-spectrum white light LED source (Xcite-120 LED, Excelitas). The system was equipped with a long-pass filter (Semrock BLP01-405R-25) and a 63× oil immersion objective (Leica HCX PL APO). All cells were given at least 24 h to re-adhere before treatment (for treated cells) and imaging. Briefly, PWS imaging is performed using a specialized microscope design that captures interference spectra resulting from the interaction between a reference wave and waves scattered from spatial refractive index variations within the coherence volume. The refractive index is proportional to the local chromatin density, which is linked to the 3D conformation of the chromatin polymer. The standard deviation of the interference spectra is proportional to the Fourier transform of the autocorrelation function of chromatin density integrated over the Fourier transform of the coherence volume. By analyzing the PWS image cubes (*x*,*y*,λ) calculating the standard deviation of pixel intensity across wavelengths, the local average chromatin packing scaling *D*_a_(*x*,*y*) can be determined for each (*x*,*y*) location within the nucleus. This calculation utilizes known optical parameters of illumination and signal acquisition, as well as the functional form of the chromatin density autocorrelation function previously measured by chromatin electron tomography. The nuclear average scaling *D*_n_ is then obtained by averaging *D*_a_(*x*,*y*) across the entire nucleus, providing a measure of the upregulation of chromatin domains within the nucleus (Eid et al., 2020; Gladstein et al., 2019; Li et al., 2021; Virk et al., 2020). Changes in *D*_n_ resulting from each condition are quantified by averaging cells, taken across the technical replicates. The average (mean) *D_n_* was calculated by first averaging *D_n_* values from PWS measurements within each cell nucleus and then averaging these measurements over the entire cell population for each treatment condition.

### Dynamic PWS measurements

Temporal PWS data was acquired as previously described (Gladstein et al., 2019; Pujadas Liwag et al., 2024). Briefly, dynamics measurements [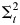, fractional moving mass (*m*_f_), and diffusion] are collected by acquiring multiple backscattered wide-field images at a single wavelength (550 nm) over time (acquisition time), to produce a three-dimensional image cube, where 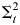 is temporal interference and *t* is time. Diffusion is extracted by calculating the decay rate of the autocorrelation of the temporal interference as previously described (Gladstein et al., 2019). The fractional moving mass is calculated by normalizing the variance of 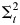 at each pixel. Using the equations and parameters supplied and explained in detail in the supplementary information of our recent publication (Gladstein et al., 2019), the FMM is obtained by using the following equation to normalize 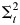 by *ρ*_0_, the density of a typical macromolecular cluster:
(1)

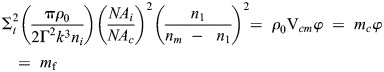
With this normalization, 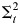 is equivalent to *m*_f_, which measures the mass moving within the sample. This value is calculated from the product of the mass of the typical moving cluster (*m*_*c*_) and the volume fraction of mobile mass (ϕ). *m*_*c*_ is obtained by *m*_*c*_=V_*cm*_*ρ*_0_, where V_*cm*_is the volume of the typical moving macromolecular cluster. To calculate this normalization, we approximate *n*_*m*_=1.43 as the refractive index (RI) of a nucleosome, *n*_1_=1.37 as the RI of a nucleus, *n*_*i*_=1.518 as the refractive index of the immersion oil, and *ρ*_0_=0.55 g *cm*^−3^ as the dry density of a nucleosome. Additionally, *k*=1.57×10^5^ cm^−1^ is the scalar wavenumber of the illumination light and Γ is a Fresnel intensity coefficient for normal incidence. *NA*_*c*_=1.49 is the numerical aperture (NA) of collection and *NA*_*i*_=0.52 is the NA of illumination. As stated previously (Gladstein et al., 2019), 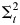 is sensitive to instrument parameters such as the depth of field and substrate refractive index. These dependencies are removed through normalization with the proper pre-factor calculated above for obtaining biological measurements. It should also be noted that backscattered intensity is prone to errors along the transverse direction (Gladstein et al., 2019). Owing to these variations, these parameters are more accurate when calculating the expected value over each pixel.

Chromatin volume concentration is calculated by Fresnel reflection coefficient. Recall that the reflectance at a RI mismatch interface:
(2)

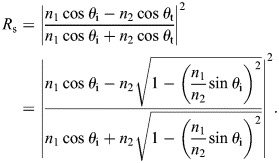

(3)

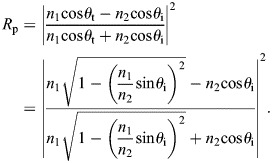
Although the chromatin is inhomogeneous and not infinitely large, the correlation between chromatin average RI and reflection coefficients still holds as confirmed by finite difference time domain (FDTD) simulations (Fig. S2). Briefly, we used our in-house FDTD software (available upon request) to simulate the entire PWS imaging system, from incident to light-matter interaction and then collection (Çapoǧlu et al., 2013). Light beams representing the characteristics of experimental *NA_i_* are first introduced to the simulation space. The simulation space contains a layer of glass and random media that represents chromatin average RI and packing behavior. The electromagnetic wave after light-matter interaction is then collected with the same *NA_c_* and far field PWS image is analyzed the same way in experiments. We did a series of simulations with different average RI and measured the mean reflection coefficients. We have fitted:
(4)

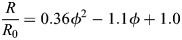
where *φ* is chromatin volume concentration, related to RI through Gladstone–Dale equation (Gladstein et al., 2019; Virk et al., 2020):
(5)

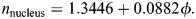


### Normalization of dynamics PWS data

Normalization for data seen in Fig. 3 is done in two ways. For a single-cell region of interest and the relative CVC change over time, the data is normalized to a range of 0,1. This is done by calculating: 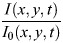 for the entire field of view, where *I*_0_ is from the reference image taken during all PWS dynamics experiments. We then take the inverse and normalize the data range to fall within 0–1. The ensemble is the average of the results applied to all cell regions of interest for a given treatment condition. The second normalization method is acquired by first calculating the average intensity across all wavelengths for a given region of interest. Then, using the established relationship between intensity and CVC, we calculate the equivalent CVC value and convert it into the range of 0 to 1.

### SMLM sample preparation and imaging

Primary antibody rabbit anti-H3K9me3 (Abcam, #ab176916, dilution 1:1000) was aliquoted and stored at −80°C. The secondary antibody goat anti-rabbit-IgG conjugated to Alexa Fluor 647 (Thermo Fisher Scientific, #A-21245, dilution 1:1000) was stored at 4°C. The cells were plated on no. 1 borosilicate bottom eight-well Lab-Tek Chambered cover glass with at a seeding density of 1.25×10^4^. After 48 h, the cells were fixed in 3% paraformaldehyde in PBS for 10 min, and then subsequently washed with PBS once for 5 min. Thereafter the samples were quenched with freshly prepared 0.1% sodium borohydride in PBS for 7 min and rinsed with PBS three times at room temperature. The fixed samples were permeabilized with a blocking buffer (3% BSA and 0.5% Triton X-100 in PBS) for 20 min and then incubated with rabbit anti-H3K9me3 in blocking buffer for 1–2 h at room temperature and rinsed with a washing buffer (0.2% BSA and 0.1% Triton X-100 in PBS) three times. The fixed samples were further incubated with the corresponding goat secondary antibody–dye conjugates, anti-rabbit-IgG conjugated to Alexa Fluor AF647, for 40 min, washed thoroughly with PBS three times at room temperature and stored at 4°C. Imaging of samples was performed on a STORM optical setup built on the commercially available Nikon Ti2 equipped with a Photometric 95B sCMOS camera and a 1.49 NA 100× oil immersion objective lens. Samples were illuminated with the MPB Communications 2RU-VFL-P-2000-647-B1R 647 nm 200 mW laser. Image acquisition was performed at 20–30 ms exposure for 10,000–15,000 frames.

### Data and image analysis

We used GraphPad Prism 10.1.1 for making all plots. For immunofluorescence imaging, maximum intensity projection of *Z*-series images was performed using FIJI software (Schindelin et al., 2012). To quantify nuclear bleb frequency, we considered blebs to be herniations that were still connected to the nuclear body. We considered ruptures to be cells that were no longer intact, and we considered micronuclei to be herniations that were no longer connected to the nuclear body and of similar sizes to nuclear blebs. For each field of view, the number of nuclei and the number of each nuclear bleb type was manually counted using FIJI. We then determined the percentages of total cells within each tested condition that displayed each nuclear bleb type.

### Super-resolution data analysis

We used the Thunder-STORM FIJI Plug-in (Ovesný et al., 2014) to apply maximum likelihood estimation fitting of a gaussian point spread function to our image stack. Localization datasets were then put into our Python script (available upon request; also see Pujadas Liwag et al., 2025) that utilized DBSCAN (epsilon=50, min_pts=3) to cluster our localized heterochromatic events. Heterochromatin domain size was estimated by fitting a polygon to the peripheral cluster points using the *scipy* Convex Hull method (https://docs.scipy.org/doc/scipy/reference/generated/scipy.spatial.ConvexHull.html). Outlier clusters smaller than twice the mean uncertainty of our localization (∼25 nm) or larger than 800 nm were removed from the analysis. Results displayed are concatenations of identified heterochromatic domains across all cells in that condition.

### Statistical analysis and quantification

Statistical analysis was performed using GraphPad Prism 10.1.1 and Microsoft Excel. Pairwise comparisons were calculated on datasets consisting of, at a minimum, biologically independent duplicate samples using a two-tailed unpaired *t*-test or Mann–Whitney test. The type of statistical test is specified in each case. Experimental data are presented either the mean±s.e.m. or mean±s.d., as stated in figure legends. *P*<0.05 was considered significant. Statistical significance levels are denoted as follows: n.s., not significant; *P<0.05; **P<0.01; ***P<0.001; ****P<0.0001. Sample numbers (number of nuclei, *n*), the number of replicates (*N*), and the type of statistical test used is indicated in figure legends.

## Supplementary Material



10.1242/joces.262161_sup1 Supplementary information
