# Supplementary material for: Nuclear blebs are associated with destabilized chromatin-packing domains
Source: J Cell Sci. 2025 Feb 11;138(3):jcs262161. doi: 10.1242/jcs.262161 (PMC11883274; doi:10.1242/jcs.262161)
Supplement: Supplementary information [file joces-138-262161-s1.pdf]

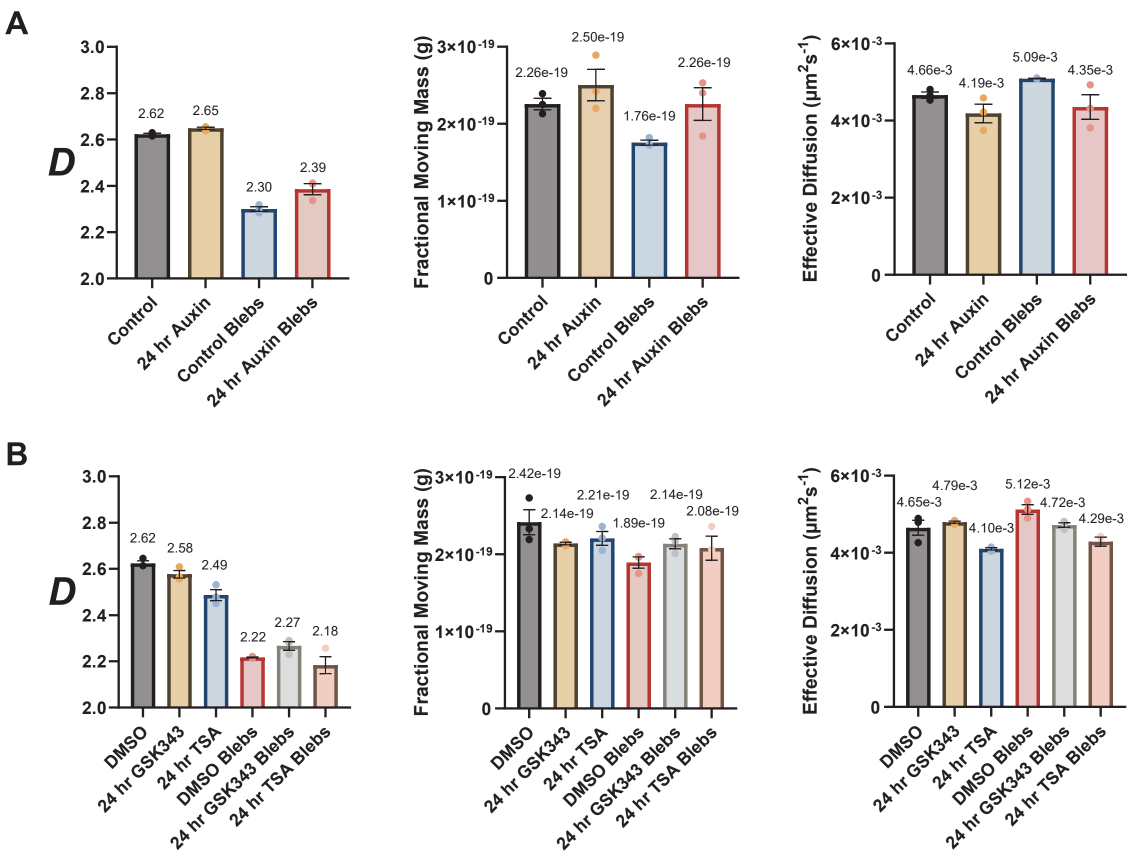

Fig. S1. Nuclear blebbing induces the redistribution and reorganization of packing domains.

(A) Averages of all three replicates for *D* values, fractional moving mass, and effective diffusion coefficient for the nuclear bodies and nuclear blebs for control and 24-hour auxin-treatment conditions in HCT116$^{LMN(B1\&B2)-AID}$ cells. **(B)** Averages of all three replicates for *D* values, fractional moving mass, and effective diffusion coefficient for DMSO (vehicle control) 24-hour GSK343, and 24-hour TSA-treatment conditions in HCT116$^{LMN(B1\&B2)-AID}$ cells. For **(A, B)** means of all replicates are presented above each bar in the plots. Error bars represent mean ± SEM. For **(A-B)**, Data are compiled from three technical replicates (N = 3; Control n = 2451, Auxin n = 2140, Control Blebs n = 200, Auxin Blebs n = 129, DMSO n = 741, GSK343 n = 790, TSA n = 498, DMSO Blebs n = 564, GSK343 Blebs n = 467, TSA Blebs n = 77).

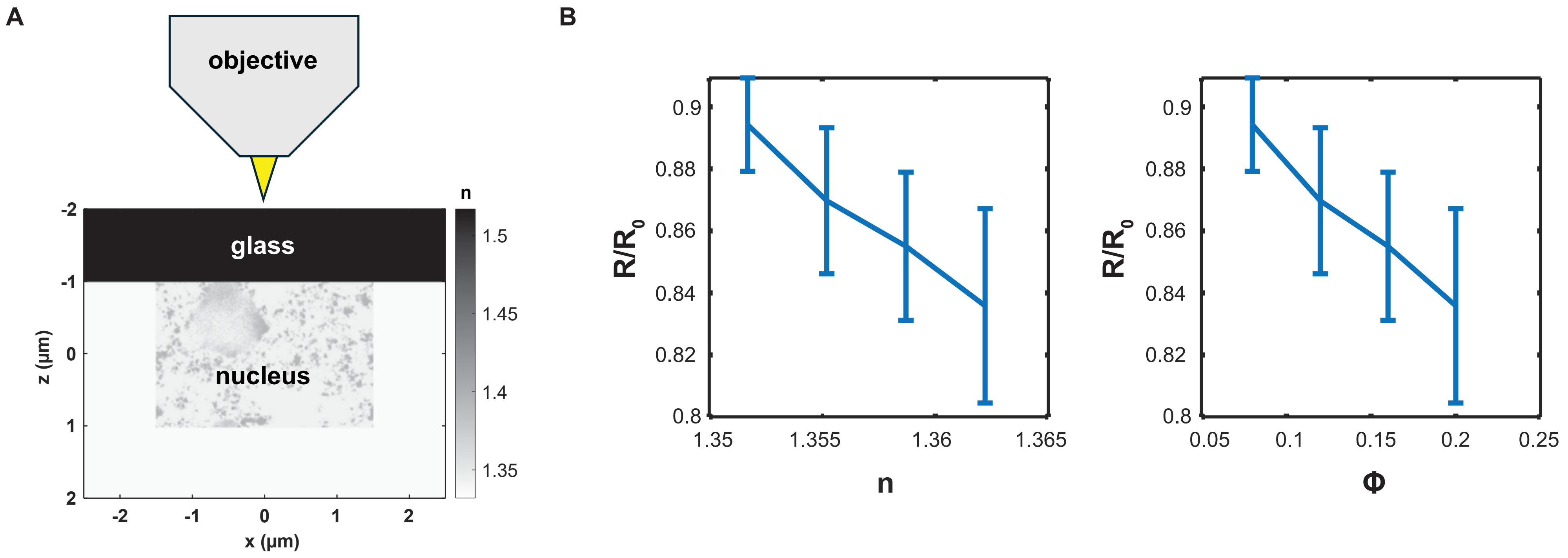

Fig. S2. FDTD simulation confirms the correlation between PWS Intensity with nuclear average RI and CVC.
(A) Schematic of FDTD simulation setup. Light is illuminated from objective and focused on the cell glass interface. Random media with autocorrelation coefficients representing chromatins are placed into the simulation space. By solving Maxwell equations numerically, the back scattering light intensity field is resolved and used to synthesize simulation PWS images. **(B)** Negative correlation between PWS normalized reflectance and media average RI and phi. n = 10 for each condition.

Table S1. Average Number of Nuclei, Blebs and Micronuclei per FOV across three replicates

	Contro l	DMSO	24 hr. Auxin	24 hr. GSK343	24 hr. Auxin + GSK 343	24 hr. TSA	24 hr. Auxin + TSA
# of Nucelli	379.50	98.33	388.50	365.33	311.00	152.00	152.00
# Blebs	7.00	1.33	21.00	24.33	25.33	26.33	37.33
# Micro-nuclei	3	0.667	18.5	9.333	9.667	12.000	13.000

Journal of Cell Science • Supplementary information

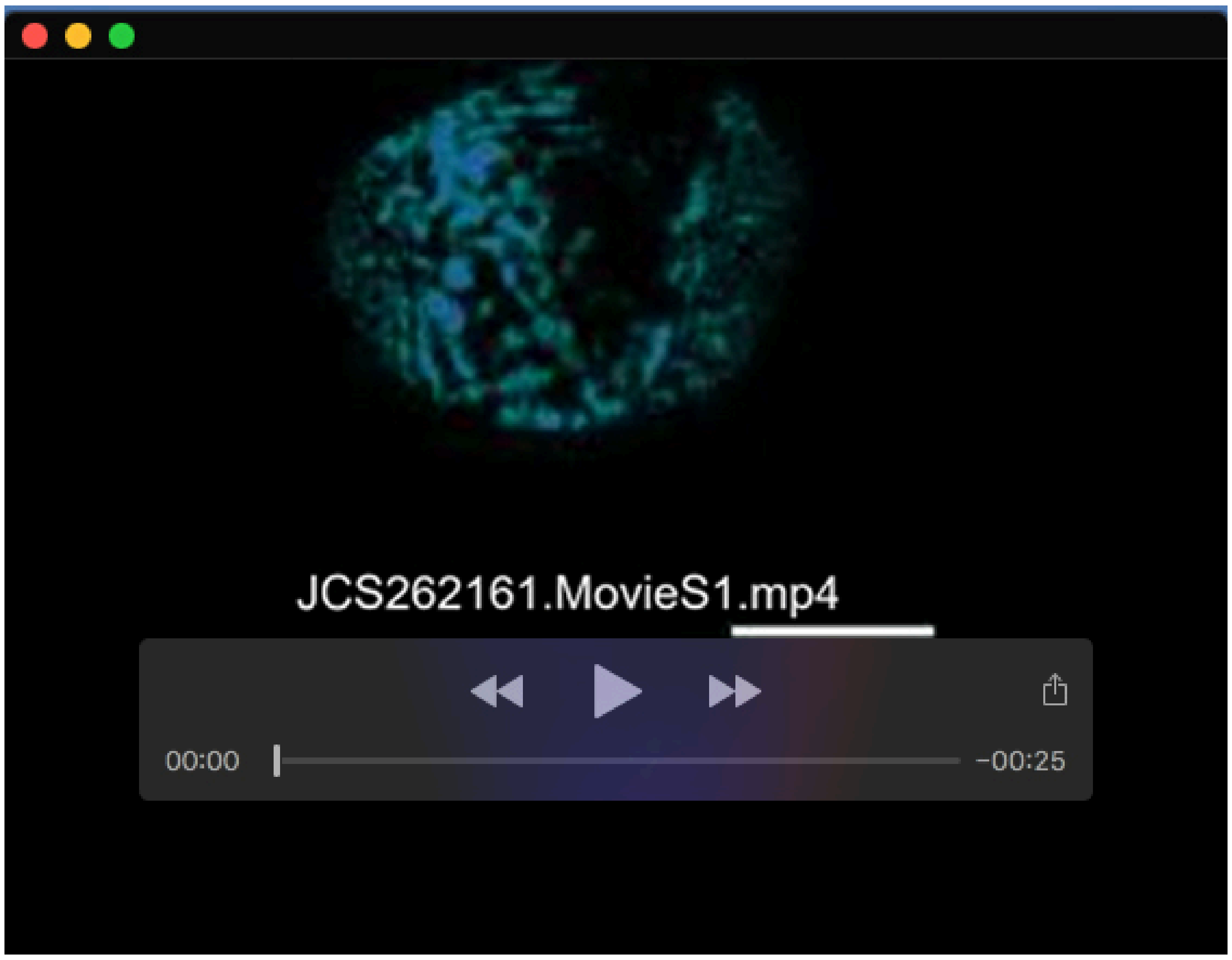

Movie 1. Fractional Moving Mass and Representative CVC time series Movies. Time series Fractional moving mass for HCT116 cell treated with DMSO corresponding to Fig. 3A Top.

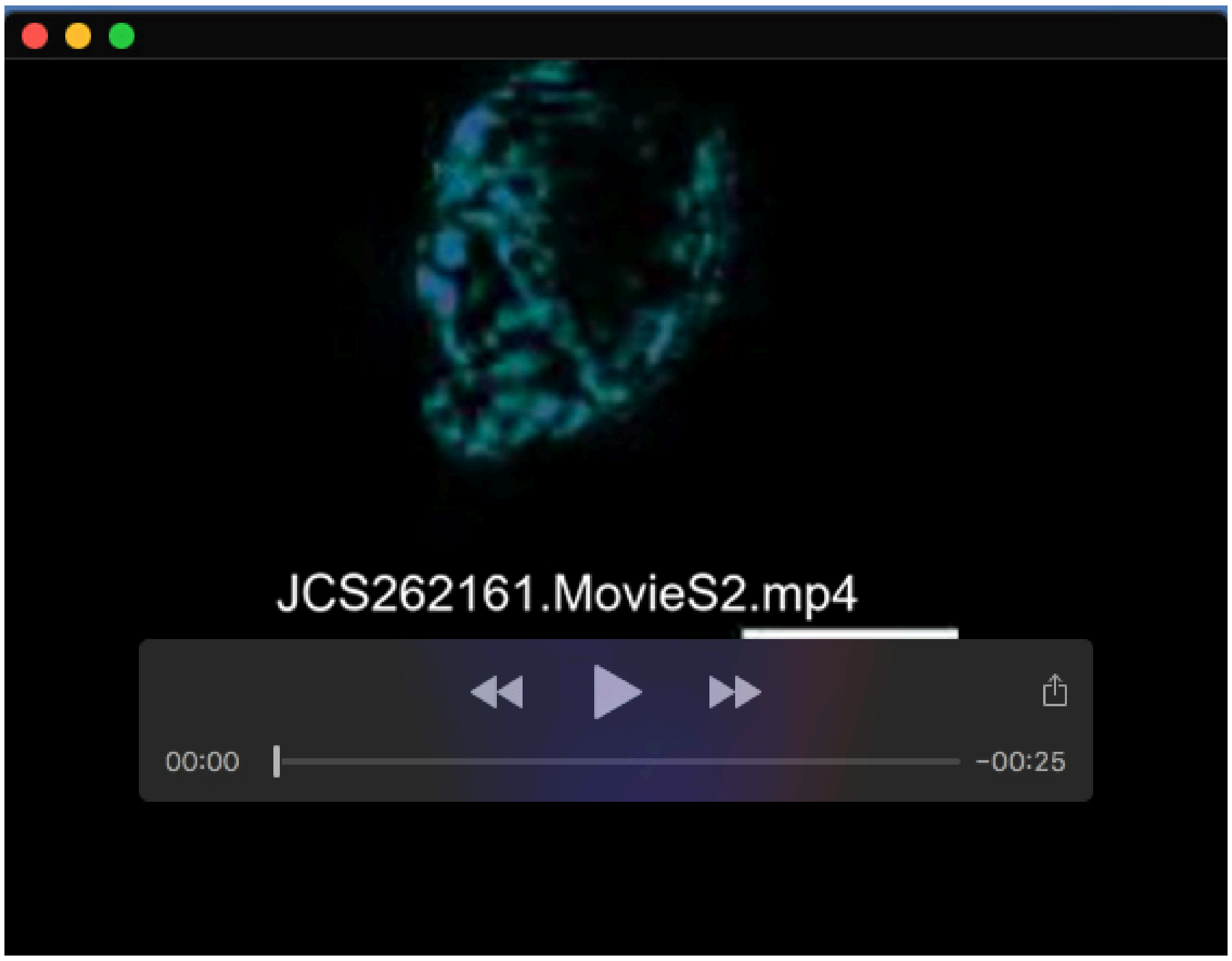

Movie 2. Fractional Moving Mass and Representative CVC time series Movies. Time series Fractional moving mass for HCT116 cell treated with TSA corresponding to Fig.3A Bottom.

Journal of Cell Science • Supplementary information

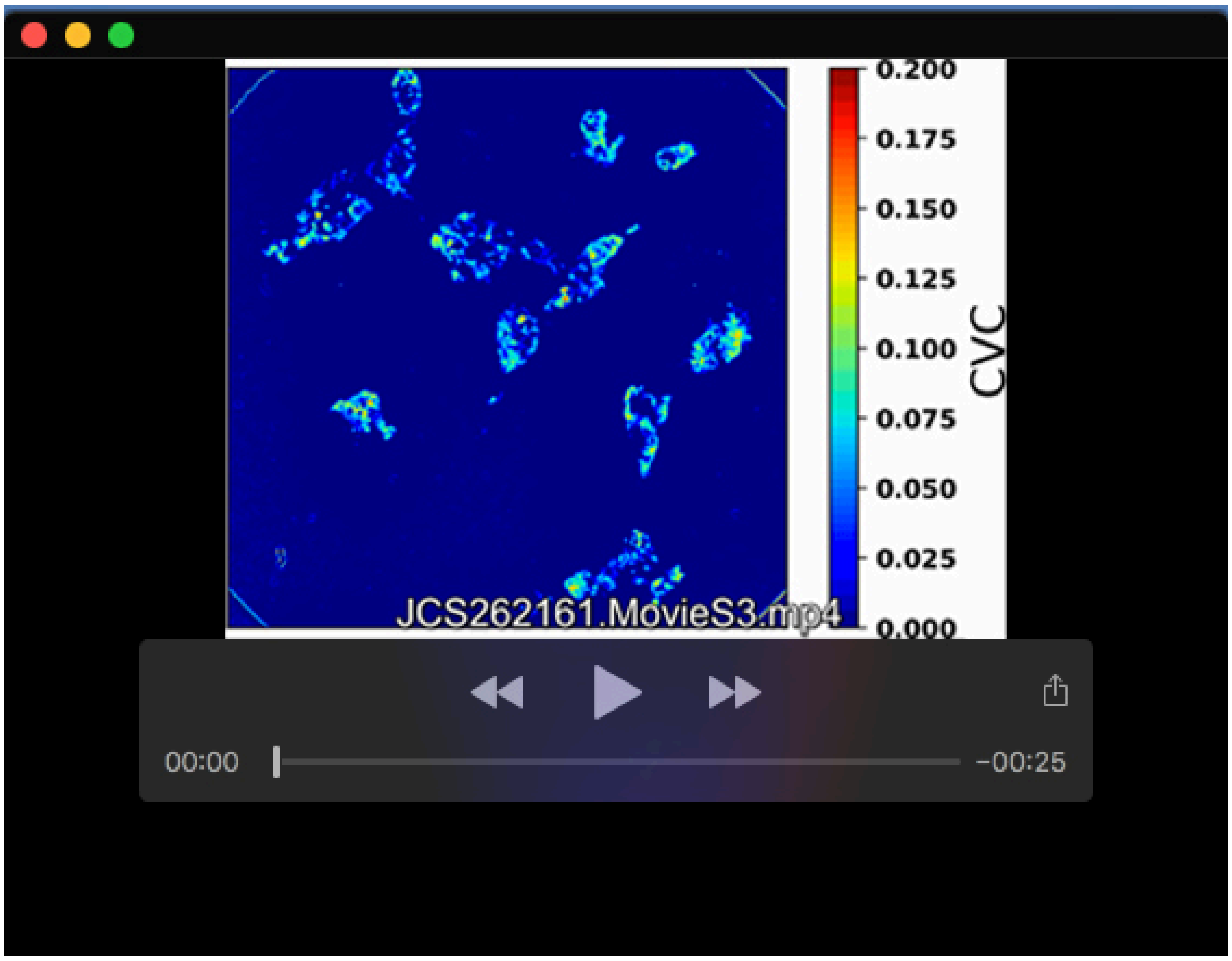

Movie 3. Fractional Moving Mass and Representative CVC time series Movies. Representative imaging of CVC time series for full field of view of HCT116 Cells.
