# Supplementary material for: Nuclear blebs are associated with destabilized chromatin-packing domains
Source: J Cell Sci. 2025 Feb 11;138(3):jcs262161. doi: 10.1242/jcs.262161 (PMC11883274; doi:10.1242/jcs.262161)
Supplement: Supplementary file 1 [file joces-138-262161_review_history.pdf]

Nuclear blebs are associated with destabilized chromatin packing domains

Emily M Pujadas Liwag, Nicolas Acosta, Luay Matthew Almassalha, Yuanzhe (Patrick) Su, Ruyi Gong, Masato T Kanemaki, Andrew D Stephens and Vadim Backman
DOI: 10.1242/jcs.262161

Editor: Guillaume Jacquemet

Review timeline

Original submission:	28 March 2024
Editorial decision:	20 May 2024
First revision received:	12 September 2024
Editorial decision:	25 October 2024
Second revision received:	26 November 2024
Accepted:	24 December 2024

Original submission

First decision letter

MS ID#: JOCES/2024/262161

MS TITLE: Nuclear blebs are associated with destabilized chromatin packing domains

AUTHORS: Emily M Pujadas Liwag, Nicolas Acosta, Luay Matthew Almassalha, Yuanzhe (Patrick) Su, Ruyi Gong, Masato T Kanemaki, Andrew D Stephens, and Vadim Backman

Dear Dr. Backman,

We have now reached a decision on the above manuscript.

To see the reviewers' reports and a copy of this decision letter, please go to: https://submit-jcs.biologists.org and click on the 'Manuscripts with Decisions' queue in the Author Area. (Corresponding author only has access to reviews.)

As you will see, the reviewers raise a number of substantial criticisms that prevent me from accepting the paper at this stage. They suggest, however, that a revised version might prove acceptable, if you can address their concerns. If you think that you can deal satisfactorily with the criticisms on revision, I would be pleased to see a revised manuscript. We would then return it to the reviewers.

Reviewer 1

Advance summary and potential significance to field

Comments were uploaded and are available as a PDF at https://submit-jcs.biologists.org.

Comments for the author

In this manuscript Emily et al., investigate the role of the nuclear lamina protein laminB and

© 2025. Published by The Company of Biologists under the terms of the Creative Commons Attribution License (https://creativecommons.org/licenses/by/4.0/).

heterochromatin enzymes in bleb formation and the structure/state and dynamics of chromatin in the blebs versus in the main nucleus. Toward that end they induce laminB degradation by employing an auxin-degron system or by employing drugs that inhibit heterochromatin (which was previously shown to cause an increase bleb in frequency). They observed an increase in frequency of nuclear blebs in all perturbations. Authors claim that domains observed within blebs were typically poorly formed, with increased fragmentation and a higher diffusion compared to the domains observed in the nuclear body. However, it was difficult to interpret the data throughout the paper, in part because the writing was not clear and in part because of what I expose below. The image and graphs shown are not convincing and the data are poorly/confusingly represented. Moreover, authors draw wrong conclusions and often interpret their own data incorrectly. Below my comments:

1- Throughout the manuscript authors interchangeably use the terms “nuclear deformations” and “nuclear blebs” as synonyms, which is not right and makes it difficult to interpret the data. Authors should explain/define the differences of each term and use only nuclear blebs (since this is their subject of study).

2- In line 166 authors write: “…and associated with severely deformed nuclear periphery (Fig. 1B; Fig. S1A)”. However, this data is not shown in these panels nor in the paper.

3- GSK343 and TSA should be used throughout the manuscript as opposed to sometimes using EZH2 and HDAC inhibitor, respectively. This also applies to all the graphs/figures. It is very confusing as it is.

4- It is impossible to understand the following sentence as it is (please revise it): “Comparing the observed behavior of chromatin domains across the nuclei in these conditions, we observed that treatment with EZH2 or TSA resulted in decreased D with an associated increase in FMM in comparison to untreated controls whereas D and FMM increased upon B-type lamin depletion, indicating that disruption of heterochromatin enzymatic processes result in fragmentation of chromatin domains and the loss of coherent chromatin motion”.

5- Authors mention that “treatment with EZH2 or TSA resulted in decreased D with an associated increase in FMM”. However, the graph in fig. 1E shows quite the opposite (unless I’m missing something major here).

6- It’s very hard to compare and interpret the data in the graphs in Figure 2 (one has to keep going back and forth between different graphs). Please put them all in the same graph.

7- The following sentence in line 223 needs revision in order to be understood/clear: “As removal of B- type lamins led to a significant increase in nuclear blebbing, these findings point to a bleb-associated chromatin phenotype, in which nuclear blebs contain fewer packing domains, and B-type lamin degradation could reduce the barriers for nuclear bleb formation that results from the restructuring or redistribution of packing domains”.

8- mClover signal is shown in figure 3 however no mention is made in the main text. Please explain and justify the use of mClover.

9- In fig. 3A authors mention that temporal interference signal is inversely proportional to chromatin density. In line 242 authors mention that: “…within a TSA treated cell with a visually apparent bleb, chromatin decreases rapidly adjacent to the nuclear bleb…”. However, the frames show no apparent changes (let alone an increase) in the temporal interference signal (which would be expected if chromatin density were to decrease).

10- In line 245 authors say: “…evidenced by increased local chromatin density and the visual changing of density in the bleb”. I could not see or understand what the authors are referring to. Can the authors point more specifically to the referred data?

11- Authors should briefly explain Single Molecule Localization Microscopy and justify this approach in the main text.

© 2025. Published by The Company of Biologists under the terms of the Creative Commons Attribution License (https://creativecommons.org/licenses/by/4.0/).

12- In line 277 authors write: "we found that the nuclear blebs arising from either B-type lamin degradation or TSA treatment contained heterochromatin around the periphery of blebs and within the center of the bleb (Fig. 4C, E, F; Fig. S2)". However, the figure panels they are referring to do not match what they are saying AND there is no heterochromatin enrichment in any bleb region of TSA-treated U2OS cells. Also, why there is no "Nuclear bleb" and "Nuclear body" for the Control U2OS cells in panel 4F (but there is "Nuclear bleb" and "Nuclear body" for the Control HCT116 cells in fig. S2)? Where are the graphs for auxin and GS343 treatments for U2OS cells? The scales in the graphs shown in panel 4F are different making it difficult to interpret the results. This entire figure needs a major revision to be interpretable.

13- In line 281 authors say: "HDAC inhibition gives rise to non-canonical nuclear blebs enriched in heterochromatin around near their boundaries and the transfer of nanoscopic heterochromatin domains into the bleb". However, the data shows no heterochromatin enrichment in the bleb and it is unclear what the authors meant by "transfer of nanoscopic heterochromatin domains into the bleb" (where is the data for that?).

14- Figure legend of figure S4 is wrong and incomplete.

15- There is no reason to have Figure 1A, B and C. Instead, they should be merge into one single graph (i.e., make Fig S1 into main figure 1).

16- All the observed changes in panels 1E, F are very minimal, so one could wonder how relevant these changes are. Authors should perform WB analysis of the histone markers.

17- In line 177 authors write: Consistent with prior reports, we observe a reduction in H3K27me3 levels upon auxin-induced degradation of B-type lamins, GSK343, and TSA treatment…". However, panel 1E shows no reduction in H3K27me3 levels upon TSA treatment (there is even a "ns" on the comparison between Control and 24hr TSA).

Reviewer 2

Advance summary and potential significance to field

This manuscript investigates chromatin associated with nuclear blebs, which are herniations of the nuclear membrane. Abnormal nuclear morphology is a hallmark of many diseases and also nuclear blebs have been reported in pathological conditions. Previous studies have shown that both the nuclear lamina and general chromatin status influence nuclear bleb formation. Nevertheless, this relationship is not fully understood.
In this study, the authors use degradation of lamin B (both B1 and B2) as well as inhibition of HDACs and histone methyltransferases, which increase euchromatin and decrease heterocromatin, respectively, and show that all of these treatments cause increased nuclear blebbing, in accordance with previous studies. They then use the interesting method of Dual-Partial Wave Spectroscopic microscopy to characterize the chromatin associated with the nuclear blebs further, and find that the chromatin within the nuclear blebs had a lower likelihood of well-formed packed domains, fragmented clutches and increased mobility compared to the overall nuclear chromatin. They also use SMLM to show somewhat surprisingly the presence of heterochromatin, although fragmented, in the nuclear blebs.
The topic of the manuscript is very interesting, since we still do not fully understand how the nuclear blebs are formed. The used methods are unique, and the result that nuclear blebs would contain heterochromatin is interesting.
However, the manuscript appears somewhat premature, fragmented and unpolished.

Comments for the author

Authors conclude that the effects of chromatin drugs and lamin B deletion are not additive. Indeed, the increase in nuclear blebs with GSK upon lamin B deletion seems negligible. Nevertheless, the

© 2025. Published by The Company of Biologists under the terms of the Creative Commons Attribution License (https://creativecommons.org/licenses/by/4.0/).

combined effect of TSA and lamin B deletion seems significant (23,56% vs 6,23 and 15,23%). Was statistics applied here?

In Fig 1E-F, the authors use immunofluorescence to study overall levels of H3K27me3 and H3K27ac upon the different treatments, and conclude that "we observe a reduction in H3K27me3 levels upon auxin-induced degradation of B-type lamins, GSK343, and TSA treatment…". However, in the graph, the effect of TSA on H3K27me3 seems not to be statistically significant, or are the labels on the bottom of the figure wrong?

On lines 184-185, it is stated that "We recently demonstrated that the decreased DNA density is conserved across multiple bleb mechanisms and is a consistently preserved feature of blebs." There is no reference to this, but I presume the authors are referring to this preprint: DOI: 10.1101/2024.02.06.579152. In this preprint figure 4, the authors report very similar results as shown here in figure 2 in terms of the effects of lamin B deletion, GSK and TSA treatments on the parameter D, although the statistical comparisons are different than presented here. Are these sets of data the same? If yes, it would be important to indicate this clearly and especially refer to the preprint.

On line 214, it is stated that sample was treated with EZH2, when the treatment is with the inhibitor, GSK343. Also, it would make it simpler for the reader, if the reference to the treatments would be consistent throughout the manuscript.
Now there is a clear switch in text between descriptions of figure 1 (Auxin GSK343, TSA) and figure 2 (EZH2i, HDACi, and lamin depletion). Overall, I recommend rewriting the description for the figure 2 for clarity. From this figure, the authors conclude that "As such, these results suggest that the domains translocating into blebs are larger in size and more stable with the disruption of the lamina compared to the other conditions". This might be an overstatement, since there are no statistical comparisons made between the different conditions, but always to the respective control. Similarly, it is stated in the discussion (line 293) that lamin deletion causes larger blebs than TSA/GSK, but the statistical comparison has not been made.

On line 244, what does "active transit" here actually refer to? Similarly, line 248 mentions "an active process".

Figure legend for figure 4 is missing description of several panels. The finding that the nuclear blebs would contain heterochromatin is interesting, and therefore this part of the manuscript should be further strengthened. The way the data is presented at the moment is not optimal. It is unclear, which inset panel shows a nuclear bleb, and which one just the nuclear periphery. How large fraction of nuclear blebs contain H3K9me3? Are they also positive for other repressive histone marks, such as H3K27me3?

The relevance and additional value of figure S1 is not obvious.

In the discussion, it is stated that "HDAC or EZH2 inhibition promoted more nuclear deformations in comparison to auxin treatment to remove B-type lamins".
The statistical comparison for this was not made, and it seems unlikely that the statement holds through at least for GSK (6,99% vs 6,23%)

Reviewer 3

Advance summary and potential significance to field

The manuscript explores the chromatin organization in nuclear blebs triggered by loss of heterochromatin induced by chemical agents using live-cell PWS-microscopy.
The manuscript addresses a very novel area in biology relevant to both physiological and pathological conditions using an emerging technique.

Comments for the author

However, the authors should address the following points before the manuscript can be accepted.

© 2025. Published by The Company of Biologists under the terms of the Creative Commons Attribution License (https://creativecommons.org/licenses/by/4.0/).

Major comments:

1. Figure 1: The authors should clearly mention what they have stained for in “Lamin B1/B2-AID (green)” in panel D.
2. Line 176-178: “Consistent with prior reports, we observe a reduction in H3K27me3 levels upon auxin induced degradation of B-type lamins, GSK343, and TSA treatment, however, only a concomitant increase in H3K27ac levels was observed in the auxin-treatment group”. The analysis of the fluorescence changes is not clear from this statement. The authors should clearly state the changes induced by GSK343 and TSA treatment compared to auxin.
3. Table 1 and Figure 2: The authors mention “Chromatin packing scaling D” in the table and figure. However, what is the functional relevance of this paraemeter is not clear. Authors should explain more on that front.
4. Figure 2, panels A and D: the authors should use a different color gradient as the differences in the chromatin organization and dynamics are not apparent. Also, the zoomed in images of the blebs formed under different treatments should be provided.
5. Figure 2, panels B, C, E and F: The authors should differentiate the measurements done on the nuclear body vs those done for the blebs.
6. Figure 3: What is the scientific basis of comparing TSA treatment with DMSO. Was TSA dissolved in DMSO.
7. Figure 4, panels A to C: Left panel should show the regions (with a box) from where the zoomed in sections are taken.
8. Figure 4: The difference in the nanodomain formation in the nuclear blebs is not clear from the STORM images obtained with anti-H3K9me3 primary antibody staining. These results do not strengthen the scientific conclusions obtained with PWS-based imaging. In my opinion, this figure and section should be removed.
9. What is the frequency of bleb formation in a field of view, i.e. how many cells compared to the total cell population show the bleb formation upon treatment with GSK343, TSA and auxin treatments. This should be clearly mentioned in the text.
10. The authors should provide a schematic at the end to illustrate where is the spatial location of the nuclear body compared to nuclear blebs and how the different treatments bring about change in the chromatin organization and dynamics.
11. The authors should compare the diffusion coefficients obtained by PWS method with other methods such as FRAP, FCS, fluorescence anisotropy measurements in the discussion.

First revision

Author response to reviewers' comments

Reviewer Comments:

Reviewer 1:

In this manuscript Emily et al., investigate the role of the nuclear lamina protein laminB and heterochromatin enzymes in bleb formation and the structure/state and dynamics of chromatin in the blebs versus in the main nucleus. Toward that end they induce laminB degradation by employing an auxin-degron system or by employing drugs that inhibit heterochromatin (which was previously shown to cause an increase bleb in frequency). They observed an increase in frequency of nuclear blebs in all perturbations. Authors claim that domains observed within blebs were typically poorly formed, with increased fragmentation and a higher diffusion compared to the domains observed in the nuclear body. However, it was difficult to interpret the data throughout the paper, in part because the writing was not clear and in part because of what I expose below. The image and graphs shown are not convincing and the data are poorly/confusingly represented. Moreover, authors draw wrong conclusions and often interpret their own data incorrectly.

Below my comments:

1-Throughout the manuscript authors interchangeably use the terms “nuclear deformations” and “nuclear blebs” as synonyms, which is not right and makes it difficult to interpret the data. Authors should explain/define the differences of each term and use only nuclear blebs (since this is their subject of study).

© 2025. Published by The Company of Biologists under the terms of the Creative Commons Attribution License (https://creativecommons.org/licenses/by/4.0/).

We appreciate the reviewers feedback and modifying the text throughout to clarify that blebs are a subset of nuclear deformations. We then rectified the text to indicate that the measurements are focused on blebs in line 71 with "Among the most radical deformations in nuclear shape is the protrusion of chromatin from the nuclear surface, known as a nuclear bleb, that are associated with pathological transformation.[2, 6, 15, 16]"

2-In line 166 authors write: "…and associated with severely deformed nuclear periphery (Fig. 1B; Fig. S1A)". However, this data is not shown in these panels nor in the paper.

We appreciate the reviewer's careful review and apologize for this omission, we have corrected the manuscript as follows:
Line 158-162: "Our results confirmed that GSK343 and TSA treatment significantly increased the percentage of blebbed nuclei within HCT-116 cells; with TSA treatment also inducing micronuclei formation and associated with a deformed nuclear periphery via increased number of blebs **(Fig. 1A)**."

We have also added arrows to the figure panel to highlight the nuclear blebs in the TSA case.

3-GSK343 and TSA should be used throughout the manuscript as opposed to sometimes using EZH2 and HDAC inhibitor, respectively. This also applies to all the graphs/figures. It is very confusing as it is.

We thank the reviewer for their feedback and simplified the manuscript to refer to EZH2 inhibition and HDAC inhibition instead of alternating between the mechanism of the drug and the agent itself.

4- It is impossible to understand the following sentence as it is (please revise it): "Comparing the observed behavior of chromatin domains across the nuclei in these conditions, we observed that treatment with EZH2 or TSA resulted in decreased D with an associated increase in FMM in comparison to untreated controls whereas D and FMM increased upon B-type lamin depletion, indicating that disruption of heterochromatin enzymatic processes result in fragmentation of chromatin domains and the loss of coherent chromatin motion".

We have revised this as follows in lines 227-231:
"Comparing the observed behavior of chromatin domains across the nuclei in these conditions, we observed that treatment with EZH2 or TSA resulted in decreased D and FMM ***(Fig. 2F, G)*** *in comparison to untreated controls. Upon B-type lamin depletion, we observed an increase in D and FMM* ***(Fig 2. B,C)****, indicating that disruption of heterochromatin enzymatic processes result in fragmentation of chromatin domains and loss of coherent chromatin motion.".*

5-Authors mention that "treatment with EZH2 or TSA resulted in decreased D with an associated increase in FMM". However, the graph in fig. 1E shows quite the opposite (unless I'm missing something major here).

We thank the reviewer for their feedback. We have pointed to the associated figure in the text (Fig. 2) as well as changed the text in lines 228-230: ", ***we observed that inhibition of with GSK343 or TSA resulted in decreased D and FMM (Fig. 2F, G) in comparison to untreated controls."***

6-It's very hard to compare and interpret the data in the graphs in Figure 2 (one has to keep going back and forth between different graphs). Please put them all in the same graph.

We have consolidated the data into a single graph as requested. All quantitative measurements were placed in a graph with 3x3 subpanels for easier navigation.

7-The following sentence in line 223 needs revision in order to be understood/clear: "As removal of B- type lamins led to a significant increase in nuclear blebbing, these findings point to a bleb-associated chromatin phenotype, in which nuclear blebs contain fewer packing domains, and B-type

© 2025. Published by The Company of Biologists under the terms of the Creative Commons Attribution License (https://creativecommons.org/licenses/by/4.0/).

lamin degradation could reduce the barriers for nuclear bleb formation that results from the restructuring or redistribution of packing domains”.

We have modified the text as follows in lines 241-246:
“When paired with the findings in the nuclear body of each respective perturbation, this suggests a potential differential mechanism of bleb translocation for each model. Specifically, heterochromatin inhibition may be producing smaller domains that are spontaneously passing through smaller initial deformation of the nucleus. By contrast, the disruption of the lamina may be resulting in larger membrane defects that facilitate the passage of otherwise stable domains..”

8-mClover signal is shown in figure 3 however no mention is made in the main text. Please explain and justify the use of mClover.

The use of mClover is a marker for the stable introduction of the cell line. We have modified the text to reflect that appropriately in lines 142-143.
“This cell line has an mClover fluorescent reporter fused to the lamin B1/B2 proteins that was used in our degradation experiments.”

Additionally, please refer to the methods section of *Depletion of lamins B1 and B2 promotes chromatin mobility and induces differential gene expression by a mesoscale-motion-dependent mechanism. Genome Biol 25, 77 (2024). https://doi.org/10.1186/s13059-024-03212-y.*

9-In fig. 3A authors mention that temporal interference signal is inversely proportional to chromatin density. In line 242 authors mention that: “…within a TSA treated cell with a visually apparent bleb, chromatin decreases rapidly adjacent to the nuclear bleb…”. However, the frames show no apparent changes (let alone an increase) in the temporal interference signal (which would be expected if chromatin density were to decrease).

This observation was in reference to the accompanying video with still captures of the events. We have added arrows to indicate the locations we are referring to on the respective image.

10-In line 245 authors say: “…evidenced by increased local chromatin density and the visual changing of density in the bleb”. I could not see or understand what the authors are referring to. Can the authors point more specifically to the referred data?

We thank the reviewer for their comment and have modified the text as follows to improve clarity in lines 265-268:
*“In the TSA treated case **(Fig. 3A bottom)**, we observe active transit of small amounts of mass between the nuclear bleb and the main nuclear body. This is evidenced by an increased local chromatin density and the temporally evolving density in the bleb **(Fig. 3A TSA t:1/200-t:200/200)**.”*

We have also added arrows to in the figure panel to indicate the area of interest with the referred change in density.

11-Authors should briefly explain Single Molecule Localization Microscopy and justify this approach in the main text.

We thank the reviewer for this comment and have edited the text to include this description of Single Molecule Localization Microscopy in lines 297-309:
“*Single Molecule Localization Microscopy (SMLM) is a super-resolution imaging technique that allows for the precise localization of individual fluorescent molecules, achieving spatial resolutions beyond the diffraction limit of light. This method enables detailed visualization of molecular structures and interactions within cells, providing insights into biological processes at the nanoscale. A specific version of this technique STORM (stochastic optical reconstruction microscopy) was used to image chromatin, specifically allows us to differentiate between distinct structures within a diffraction limited volume or the ensemble behavior of mass. In the*

© 2025. Published by The Company of Biologists under the terms of the Creative Commons Attribution License (https://creativecommons.org/licenses/by/4.0/).

manuscript, we chose to image the positioning of H3K9me3 as it's associated with high-density regions that are observed to form the centroids of domain cores on electron microscopy. By pairing this with dual-PWS microscopy, we can investigate both the ensemble behavior of chromatin throughout the nucleus and the nanoscopic structure of high-density regions formed by heterochromatin. This is achieved with its high resolution capable of resolving nanoscopic structures between 10-30nm"

12-In line 277 authors write: "we found that the nuclear blebs arising from either B-type lamin degradation or TSA treatment contained heterochromatin around the periphery of blebs and within the center of the bleb (Fig. 4C, E, F; Fig. S2)".

1. However, the figure panels they are referring to do not match what they are saying AND there is no heterochromatin enrichment in any bleb region of TSA-treated U2OS cells.
2. Also, why there is no "Nuclear bleb" and "Nuclear body" for the Control U2OS cells in panel 4F (but there is "Nuclear bleb" and "Nuclear body" for the Control HCT116 cells in fig. S2)? Where are the graphs for auxin and GSK343 treatments for U2OS cells?
3. The scales in the graphs shown in panel 4F are different making it difficult to interpret the results. This entire figure needs a major revision to be interpretable.

We have modified the manuscript as follows:

1. **Figure 4 and Fig. S2 has been split into two figures (Figure 4 and Figure 5). This was done to emphasize the purpose of each dataset. While both used SMLM to visualize heterochromatin in the nuclear body and blebs, the HCT116 lamin degraded dataset was used to visually confirm findings from PWS. The U2OS dataset in figure 5 was used to quantify these nanodomains statistically as the n for HCT116 cells was low. In each figure we have added arrows to clearly dictate the presence of heterochromatin both in the periphery and interior of these nuclear blebs in the lamin degradation and TSA treatment case. Quantification of these images have been placed along with the respective cell line. The manuscript text has been modified to reflect these changes between lines in the SMLM results section.**
2. **There is no CTRL DMSO nuclear bleb and nuclear body plot as the U2OS cell line has lower frequency of blebbing and no bleb images in the DMSO case were obtained. Only TSA was shown here as this treatment has been shown (Fig. 1A) to cause the highest percentage of nuclear blebs and as such was**

the pharmacological agent used to perturb the cells and induce nuclear blebbing.

3. **The scales are different as the number of heterochromatin localizations (H3K9me3) captured in the nuclear body vs nuclear bleb are different. The nuclear body has upwards of ~20,000 localizations while the bleb has less than ~8,000. As such, clustering algorithms will find less clusters in the bleb by default, so we chose a smaller range for our count histogram so the data can be visualized. If the same scales were used, the bleb graph would not be seen as its scale (<100) is far smaller than the nuclear body (<3500).**

13-In line 281 authors say: "HDAC inhibition gives rise to non-canonical nuclear blebs enriched in heterochromatin around near their boundaries and the transfer of nanoscopic heterochromatin domains into the bleb". However, the data shows no heterochromatin enrichment in the bleb and it is unclear what the authors meant by "transfer of nanoscopic heterochromatin domains into the bleb" (where is the data for that?).

We thank the reviewer for this comment. The stained molecule is constitutive heterochromatin, H3K9me3, which is clearly visualized within the nuclear bleb. SMLM utilizes a time acquisition of blinking fluorophores (~10,000 frames, 30 ms exposure). This data is used to reconstruct the images shown in this figure with brighter pixels representing the presence of many localizations within that area. The inset for Fig. 5B with the added arrow shows bright areas in the bleb near the periphery representing the enrichment for heterochromatin we are referring to for the TSA treatment case.

14-Figure legend of figure S4 is wrong and incomplete.

We apologize for the confusion, but there is no fourth figure in supplementary figures. We have revised all legends and titles

© 2025. Published by The Company of Biologists under the terms of the Creative Commons Attribution License (https://creativecommons.org/licenses/by/4.0/).

15-There is no reason to have Figure 1A, B and C. Instead, they should be merged into one single graph (i.e., make Fig S1 into main figure 1).

We have merged this into one graph per the reviewer's request

16-All the observed changes in panels 1E, F are very minimal, so one could wonder how relevant these changes are. Authors should perform WB analysis of the histone markers.

We have previously demonstrated that these histone marks are changed with WB analysis in the cell population. Western blot analysis for these treatments have been done in https://doi.org/10.1091/mbc.E17-06-0410 which corroborate our results. Further, these agents are widely used for this exact intended purpose.

17-In line 177 authors write: Consistent with prior reports, we observe a reduction in H3K27me3 levels upon auxin-induced degradation of B-type lamins, GSK343, and TSA treatment…". However, panel 1E shows no reduction in H3K27me3 levels upon TSA treatment (there is even a "ns" on the comparison between Control and 24hr TSA).

We apologize for this mistake; we have modified the text as follows in lines 174-176:
"Consistent with prior reports, we observe a reduction in H3K27me3 levels upon auxin-induced degradation of B-type lamins, GSK343, however, only a concomitant increase in H3K27ac levels was observed in the auxin-treatment group ***(Fig. 1E, F)****."*

Reviewer 2
Comments for the Author:
Authors conclude that the effects of chromatin drugs and lamin B deletion are not additive. Indeed, the increase in nuclear blebs with GSK upon lamin B deletion seems negligible. Nevertheless, the combined effect of TSA and lamin B deletion seems significant (23,56% vs 6,23 and 15,23%). Was statistics applied here?

Due to the infrequent nature of blebs, we refrained from multiple comparison testing in this manuscript. We have previously demonstrated that bleb frequency increases on combination treatment with a chromatin modifying agent and with depletion of b-type Lamins.

We have added a sentence here highlighting that in contrast to prior work, we do not see an increase in blebs for additive treatment cases. We apologize for not including this statistical test in the initial submission.
"Combination of depletion of B-type lamins with disruption of heterochromatin resulted in only slightly increased rates of nuclear blebs in comparison to lamin depletion, GSK343, or TSA treatment alone (No significant Difference: Auxin + GSK343 7.97%, Auxin + TSA 23.56%) (Fig. 1A). We found no significant difference between cells treated with or without auxin ini both TSA- and GSK343- treated groups (TSA vs TSA+Auxin P = 0.078, and GSK343 vs GSK343+Auxin P=0.8235)."

In Fig 1E-F, the authors use immunofluorescence to study overall levels of H3K27me3 and H3K27ac upon the different treatments and conclude that "we observe a reduction in H3K27me3 levels upon auxin-induced degradation of B-type lamins, GSK343, and TSA treatment…". However, in the graph, the effect of TSA on H3K27me3 seems not to be statistically significant, or are the labels on the bottom of the figure wrong?

We apologize for this mistake and have corrected the text appropriately. We measured H3K9me3 levels with TSA treatment and verified the depletion of heterochromatin. We have modified the manuscript text as follows in lines 174-176:
"Consistent with prior reports, we observe a reduction in H3K27me3 levels upon auxin-induced degradation of B-type lamins, GSK343, however, only a concomitant increase in H3K27ac levels was observed in the auxin-treatment group ***(Fig. 1E, F)****."*

© 2025. Published by The Company of Biologists under the terms of the Creative Commons Attribution License (https://creativecommons.org/licenses/by/4.0/).

On lines 184-185, it is stated that "We recently demonstrated that the decreased DNA density is conserved across multiple bleb mechanisms and is a consistently preserved feature of blebs." There is no reference to this, but I presume the authors are referring to this preprint: DOI: 10.1101/2024.02.06.579152. In this preprint figure 4, the authors report very similar results as shown here in figure 2 in terms of the effects of lamin B deletion, GSK and TSA treatments on the parameter D, although the statistical comparisons are different than presented here. Are these sets of data the same? If yes, it would be important to indicate this clearly and especially refer to the preprint.

We have clarified this within the manuscript as follows by adding the citation to this data in lines 181-182:

"We recently demonstrated that the decreased DNA density is conserved across multiple bleb mechanisms and is a consistently preserved feature of blebs (Bunner, Prince, Srikirshna, et al. 2024)."

On line 214, it is stated that sample was treated with EZH2, when the treatment is with the inhibitor, GSK343. Also, it would make it simpler for the reader, if the reference to the treatments would be consistent throughout the manuscript. Now there is a clear switch in text between descriptions of figure 1 (Auxin, GSK343, TSA) and figure 2 (EZH2i, HDACi, and lamin depletion). Overall, I recommend rewriting the description for the figure 2 for clarity. From this figure, the authors conclude that "As such, these results suggest that the domains translocating into blebs are larger in size and more stable with the disruption of the lamina compared to the other conditions". This might be an overstatement, since there are no statistical comparisons made between the different conditions, but always to the respective control. Similarly, it is stated in the discussion (line 293) that lamin deletion causes larger blebs than TSA/GSK, but the statistical comparison has not been made.

We thank the reviewer for their comments here. We have adjusted the description of figure 2 for clarity as follows:

*"**(A)** Representative PWS D maps **(B)** and D values for the nuclear bodies and nuclear blebs for control and 24-hour auxin-treatment conditions in HCT116$^{LMN(B1\&B2)-AID}$ cells. **(C)** Fractional moving mass and **(D)** Effective diffusion coefficient values for nuclear bodies and nuclear blebs for control and 24-hour auxin-treatment conditions in HCT116$^{LMN(B1\&B2)-AID}$ cells **(E)** Representative PWS D maps and **(F)** D values for the nuclear bodies and nuclear blebs for DMSO (vehicle control), 24-hour GSK343, and 24-hour TSA-treatment conditions in HCT116$^{LMN(B1\&B2)-AID}$ cells. **(G)** Fractional moving mass values and **(H)** Effective diffusion coefficient value for the nuclear bodies and nuclear blebs for DMSO (vehicle control, 24-hour GSK343, and 24-hour TSA-treatment conditions in HCT116$^{LMN(B1\&B2)-AID}$. For **(B-D,F-H)**, each dot represents a cell nucleus. (Control n = 2451, Auxin n = 2140, Control Blebs n = 200, Auxin Blebs n = 129, DMSO n = 741, GSK343 n = 790, TSA n = 498, DMSO Blebs n = 564, GSK343 Blebs n = 467, TSA Blebs n = 77). Error bars represent mean ± SD. Data are compiled from three technical replicates (N = 3). Violin plots show the median and quartiles for the unpaired two-tailed t-test between selected groups. *P ≤ 0.05, **P ≤ 0.01, ***P ≤ 0.001, ****P ≤ 0.0001. Scale bars = 5 µm."*

On line 244, what does "active transit" here actually refer to? Similarly, line 248 mentions "an active process".

We thank the reviewer for the opportunity to clarify the manuscript. We can understand that the initial phrasing "active transit" and "active process" can connotate that a kinetic activity is occurring to direct mass from the nucleus and into the bleb. Instead, our point was that analysis of mass within the bleb and the boundary of the nucleus at the bleb indicates that density is changing at the boundary as would be consistent with communication between these two partitions. This was visually observed by measuring the relative change in density over time at the area adjacent to the bleb and within the bleb itself. We have modified the text to clarify this as follows:

"In the TSA treated case **(Fig. 3A bottom)**, we observe transit of small amounts of mass between the nuclear bleb and the main nuclear body"

© 2025. Published by The Company of Biologists under the terms of the Creative Commons Attribution License (https://creativecommons.org/licenses/by/4.0/).

We also thank the reviewer for the comment regarding the comparison of domains traveling into nuclear blebs after lamin depletion or heterochromatin inhibition. We have clarified the text as shown below in lines 240-245.

"When paired with the findings in the nuclear body of each respective perturbation, this suggests a potential differential mechanism of bleb translocation for each model. Specifically, heterochromatin inhibition may be producing smaller domains that are spontaneously passing through smaller initial deformation of the nucleus. By contrast, the disruption of the lamina may be resulting in larger membrane defects that facilitate the passage of otherwise stable domains."

Figure legend for figure 4 is missing description of several panels. The finding that the nuclear blebs would contain heterochromatin is interesting, and therefore this part of the manuscript should be further strengthened. The way the data is presented at the moment is not optimal. It is unclear, which inset panel shows a nuclear bleb, and which one just the nuclear periphery. How large fraction of nuclear blebs contain H3K9me3? Are they also positive for other repressive histone marks, such as H3K27me3?

We thank the reviewer for this feedback and have modified the text to clarify this as follows. We have split figure 4 into two figures that focus on the results from HCT116 and U2OS cells. Insets are now labeled for nuclear body/periphery and nuclear bleb. From our imaging results, every bleb observed and imaged with SMLM has H3K9me3, however this is due to the fact we only labeled H3K9me3. We have added two-color SMLM data for HCT116 in figure 4 and can see that our blebs contain RNAP II. We did not label for H3K27me3 so we cannot declare the presence of this mark within our blebs, however we theorize for this to be the case.

The relevance and additional value of figure S1 is not obvious.

We thank the reviewer for their comment and have replaced Fig. 1A,B,C with the consolidated plot of seen in Fig. S1A.

In the discussion, it is stated that "HDAC or EZH2 inhibition promoted more nuclear deformations in comparison to auxin treatment to remove B-type lamins". The statistical comparison for this was not made, and it seems unlikely that the statement holds through at least for GSK (6,99% vs 6,23%)

We thank the reviewer for their comment, and we agree that these values are comparable. We refer to SI figure 6 in additional file 2 prior publication here: https://doi.org/10.1186/s13059-024-03212-y.

*****

Reviewer 3

Advance Summary and Potential Significance to Field:

The manuscript explores the chromatin organization in nuclear blebs triggered by loss of heterochromatin induced by chemical agents using live-cell PWS-microscopy. The manuscript addresses a very novel area in biology relevant to both physiological and pathological conditions using an emerging technique.

Reviewer 3 Comments for the Author:

However, the authors should address the following points before the manuscript can be accepted.

Major comments:

1.Figure 1: The authors should clearly mention what they have stained for in "Lamin B1/B2-AID (green)" in panel D.

© 2025. Published by The Company of Biologists under the terms of the Creative Commons Attribution License (https://creativecommons.org/licenses/by/4.0/).

We appreciate the reviewers comment and have edited the manuscript as follows:
"Therefore, we first investigated the role of processes well established to induce bleb formation: inhibition of B-type lamins and disruption in heterochromatin enzymes.[2, 34] To assess the impact of lamin degradation on nuclear morphology, we applied the AID system to HCT116 colorectal carcinoma epithelial cells to induce simultaneous degradation of lamin B1 and lamin B2 as previously described.[32, 33, 35]. This cell line has an mClover fluorescent reporter fused to the lamin B1/B2 proteins that was used in our degradation experiments."

2.Line 176-178: "Consistent with prior reports, we observe a reduction in H3K27me3 levels upon auxin induced degradation of B-type lamins, GSK343, and TSA treatment, however, only a concomitant increase in H3K27ac levels was observed in the auxin-treatment group". The analysis of the fluorescence changes is not clear from this statement. The authors should clearly state the changes induced by GSK343 and TSA treatment compared to auxin.

We apologize for this mistake and have corrected the text appropriately. We have modified the manuscript text as follows in lines 174-176:
*"Consistent with prior reports, we observe a reduction in H3K27me3 levels upon auxin-induced degradation of B-type lamins, GSK343, however, only a concomitant increase in H3K27ac levels was observed in the auxin-treatment group **(Fig. 1E, F)**."*
3.Table 1 and Figure 2: The authors mention "Chromatin packing scaling D" in the table and figure. However, what is the functional relevance of this parameter is not clear. Authors should explain more on that front.

We apologize for not emphasizing the importance of this parameter in our work. We have included additional clarification of D as follows:
Lines 191-206:
"As previously mentioned, chromatin structure can be quantified by how its mass fills an occupied volume using the relationship between, D, known as the packing density scaling or mass fractal dimension, quantifies the relationship between the genomic length N and the radius of the occupied volume r within chromatin structures. In principle, mass scaling can be quantified at various levels of genome organization: including an entire chromosome or the genomic scaling within individual chromatin packing domains [29]. We focus on supra-nucleosomal domain length-scales (50-150nm) within this work due to recent observations demonstrating that this regime is paired with crucial functions such as gene transcription [30.44]. Partial Wave Spectroscopy (PWS) enables the measurement of local average chromatin packing scaling (Da) at specific (x,y) locations within the nucleus with sensitivity primarily to structures within the length-scales of chromatin domains (50-200nm). Da(x,y) is proportional to the average packing scaling within the coherence volume probed. The nuclear average scaling (Dn), obtained by averaging Da(x,y) across the nucleus, is proportional to the volume fraction of chromatin domains within the nucleus and their scaling D. As chromatin is organized in mass-fractal packing domains, Dn measures the likelihood of chromatin organizing into domains with the ensemble scaling.."
We also refer the reviewer to the text here for further clarification on D and its importance:
Lines 514-531:
". Briefly, PWS imaging is performed using a specialized microscope design that captures interference spectra resulting from the interaction between a reference wave and waves scattered from spatial refractive index variations within the coherence volume. The refractive index is proportional to the local chromatin density, which is linked to the 3D conformation of the chromatin polymer. The standard deviation of the interference spectra is proportional to the Fourier transform of the autocorrelation function of chromatin density integrated over the Fourier transform of the coherence volume. By analyzing the PWS image cubes and calculating the standard deviation of pixel intensity across wavelengths, the local average chromatin packing scaling $D_a(x,y)$ can be determined for each (x,y) location within the nucleus. This calculation utilizes known optical parameters of illumination and signal acquisition, as well as the functional form of the chromatin density autocorrelation function previously measured by chromatin electron tomography. The nuclear average scaling D_n is then obtained by averaging Da(x,y) across the entire nucleus, providing a measure of the upregulation of chromatin domains within the nucleus [30, 39, 41, 48]. Changes in D_n resulting from each condition are quantified by averaging cells, taken across 3 technical replicates. Average D_n was calculated by first averaging D_n values from PWS

© 2025. Published by The Company of Biologists under the terms of the Creative Commons Attribution License (https://creativecommons.org/licenses/by/4.0/).

measurements within each cell nucleus and then averaging these measurements over the entire cell population for each treatment condition."

We also provide more context with our description here.
The parameter D, which represents the packing density scaling or mass fractal dimension of chromatin, is crucial in understanding the complex organization and conformation of chromatin within the nucleus. In our work, we focus on the nuclear average chromatin packing scaling (Dn), which is measured using Partial Wave Spectroscopy (PWS). This non-invasive optical spectroscopic nanosensing technique allows us to quantify the local average chromatin packing scaling (Da) at specific locations within the nucleus. Da(x,y) is proportional to the average packing scaling within the coherence volume, while Dn, obtained by averaging Da(x,y) across the entire nucleus, is proportional to the volume fraction of chromatin domains within the nucleus. As chromatin is organized in mass-fractal packing domains, Dn serves as a measure of the upregulation of chromatin domains. By investigating the relationship between Dn and various cellular phenomena, our work aims to shed light on the role of chromatin conformation in regulating cellular behavior and potentially identify new targets for therapeutic interventions.

4.Figure 2, panels A and D: the authors should use a different color gradient as the differences in the chromatin organization and dynamics are not apparent. Also, the zoomed in images of the blebs formed under different treatments should be provided.

We agree and have included the following different gradient in the updated figure 2.

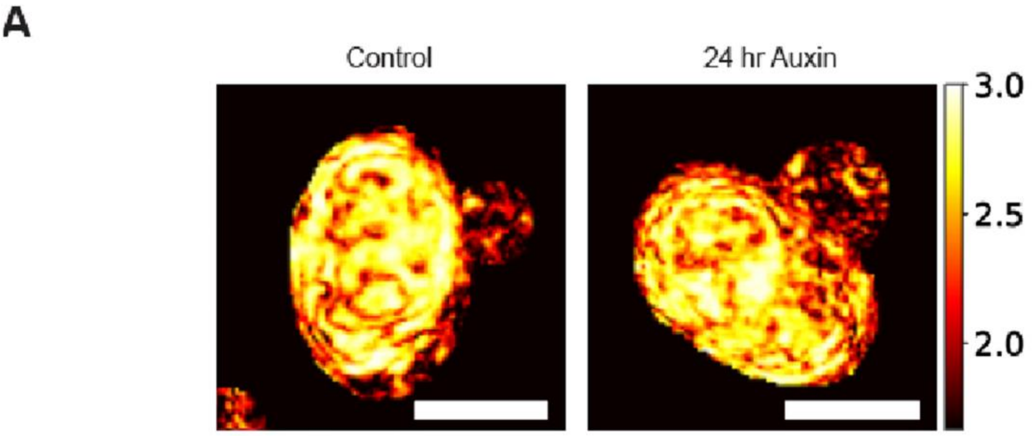

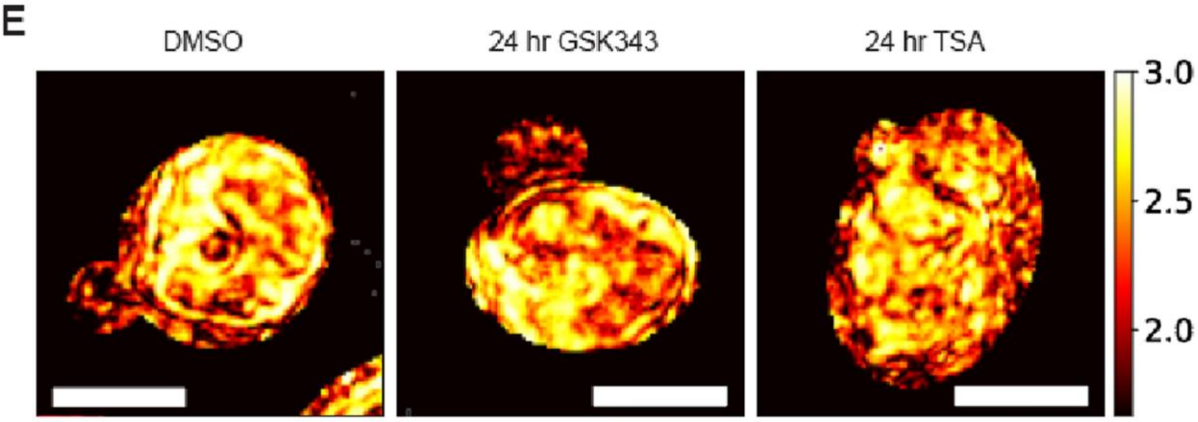

© 2025. Published by The Company of Biologists under the terms of the Creative Commons Attribution License (https://creativecommons.org/licenses/by/4.0/).

5.Figure 2, panels B, C, E and F: The authors should differentiate the measurements done on the nuclear body vs those done for the blebs.

We thank the reviewer for this feedback and have modified the manuscript accordingly. We have changed the layout of the figures and have marked which distributions refer to blebs and which to nuclear body.

6.Figure 3: What is the scientific basis of comparing TSA treatment with DMSO. Was TSA dissolved in DMSO.

DMSO was used as the solvent for TSA at concentration of 300 nM. We modified the materials and methods to justify this

7.Figure 4, panels A to C: Left panel should show the regions (with a box) from where the zoomed in sections are taken.

We have modified the figure as suggested and included it below. The figure itself has been split into two figures for clarity, with figure 4 detailing results for HCT116 and figure 5 U2OS.

8.Figure 4: The difference in the nanodomain formation in the nuclear blebs is not clear from the STORM images obtained with anti-H3K9me3 primary antibody staining. These results do not strengthen the scientific conclusions obtained with PWS-based imaging. In my opinion, this figure and section should be removed.

We understand that under the initial presentation of the manuscript it was not evident. The purpose of figure 4 has been re-worked where we show qualitative results for HCT116 cells. STORM here captures the nanodomains that PWS is sensitive to; PWS is sensitive to these packing domains while STORM complements this method by showing the domains through labeling of H3K9me3. Figure 5 then shows quantitative analysis of these domains in U2OS cells. Please see the new figure 4 and five, as well as description of SMLM within the text jn lines 254-257.

9.What is the frequency of bleb formation in a field of view, i.e. how many cells compared to the total cell population show the bleb formation upon treatment with GSK343, TSA and auxin treatments. This should be clearly mentioned in the text.

We thank the reviewer and have included a supplemental table with this information. Please see Supplementary Information Table 1 and reference directly when discussing the data in the text.

10.The authors should provide a schematic at the end to illustrate where is the spatial location of the nuclear body compared to nuclear blebs and how the different treatments bring about change in the chromatin organization and dynamics.

We appreciate this comment from the reviewer and agree that it greatly strengthens the understanding of the experiments as these are novel methods in the field. We have included this in the new figure 4 demonstrating the cumulative results in the context of domains and the distinct measurements performed.

11.The authors should compare the diffusion coefficients obtained by PWS method with other methods such as FRAP, FCS, fluorescence anisotropy measurements in the discussion.

We appreciate the reviewer's suggestion and have done FRAP in conjunction with dual-PWS as follows. Dual-PWS microscopy was previously validated on here: https://doi.org/10.1038/s41467-019-09717-6

© 2025. Published by The Company of Biologists under the terms of the Creative Commons Attribution License (https://creativecommons.org/licenses/by/4.0/).

Second decision letter

MS ID#: JOCES/2024/262161

MS TITLE: Nuclear blebs are associated with destabilized chromatin packing domains

AUTHORS: Emily M Pujadas Liwag, Nicolas Acosta, Luay Matthew Almassalha, Yuanzhe (Patrick) Su, Ruyi Gong, Masato T Kanemaki, Andrew D Stephens, and Vadim Backman

We have now reached a decision on the above manuscript.

To see the reviewers' reports and a copy of this decision letter, please go to: https://submit-jcs.biologists.org and click on the 'Manuscripts with Decisions' queue in the Author Area. (Corresponding author only has access to reviews.)

As you will see, the reviewers gave favourable reports but raised some critical points that will require amendments to your manuscript. I hope that you will be able to carry these out because I would like to be able to accept your paper, depending on further comments from reviewers.

Please ensure that you clearly highlight all changes made in the revised manuscript. Please avoid using 'Tracked changes' in Word files as these are lost in PDF conversion.

I should be grateful if you would also provide a point-by-point response detailing how you have dealt with the points raised by the reviewers in the 'Response to Reviewers' box. Please attend to all of the reviewers' comments. If you do not agree with any of their criticisms or suggestions please explain clearly why this is so.

Reviewer 1

Advance summary and potential significance to field

-

Comments for the author

In this manuscript Emily et al., investigate the role of the nuclear lamina protein laminB and heterochromatin enzymes in bleb formation and the structure/state and dynamics of chromatin in the blebs versus in the main nucleus. Toward that end they induce laminB degradation by employing an auxin-degron system or by employing drugs that inhibit heterochromatin (which was previously shown to cause an increase bleb in frequency). They observed an increase in frequency of nuclear blebs in all perturbations. Authors claim that domains observed within blebs were typically poorly formed, with increased fragmentation and a higher diffusion compared to the domains observed in the nuclear body. However, it was difficult to interpret the data throughout the paper, in part because the writing was not clear and in part because of what I expose below. The image and graphs shown are not convincing and the data are poorly/confusingly represented. Moreover, authors draw wrong conclusions and often interpret their own data incorrectly.

Below my comments:
1- Throughout the manuscript authors interchangeably use the terms “nuclear deformations” and “nuclear blebs” as synonyms, which is not right and makes it difficult to interpret the data. Authors should explain/define the differences of each term and use only nuclear blebs (since this is their subject of study).

© 2025. Published by The Company of Biologists under the terms of the Creative Commons Attribution License (https://creativecommons.org/licenses/by/4.0/).

We appreciate the reviewers feedback and modifying the text throughout to clarify that blebs are a subset of nuclear deformations. We then rectified the text to indicate that the measurements are focused on blebs in line 71 with "Among the most radical deformations in nuclear shape is the protrusion of chromatin from the nuclear surface, known as a nuclear bleb, that are associated with pathological transformation.[2, 6, 15, 16]"

The nuclear bleb is not necessarily a chromatin protrusion. It can also be a result of a local nuclear envelope detachment from the underlying nuclear lamina devoid of chromatin (as the lamina might still be intact). Subsequently, upon nuclear lamina disruption/rupture the chromatin can then also gain access to the bleb (see ref https://doi.org/10.1016/j.ceb.2021.01.007)

2- In line 166 authors write: "…and associated with severely deformed nuclear periphery (Fig. 1B; Fig. S1A)". However, this data is not shown in these panels nor in the paper.

We appreciate the reviewer's careful review and apologize for this omission, we have corrected the manuscript as follows:
Line 158-162: "Our results confirmed that GSK343 and TSA treatment significantly increased the percentage of blebbed nuclei within HCT-116 cells; with TSA treatment also inducing micronuclei formation and associated with a deformed nuclear periphery via increased number of blebs **(Fig. 1A).**"

Again, the revised data still does not show any image or quantification of micronuclei (!).

We have also added arrows to the figure panel to highlight the nuclear blebs in the TSA case.

3- GSK343 and TSA should be used throughout the manuscript as opposed to sometimes using EZH2 and HDAC inhibitor, respectively. This also applies to all the graphs/figures. It is very confusing as it is.

We thank the reviewer for their feedback and simplified the manuscript to refer to EZH2 inhibition and HDAC inhibition instead of alternating between the mechanism of the drug and the agent itself.

Ok.

We have revised this as follows in lines 227-231:
*"Comparing the observed behavior of chromatin domains across the nuclei in these conditions, we observed that treatment with EZH2 or TSA resulted in decreased D and FMM **(Fig 2F, G)** in comparison to untreated controls. Upon B-type lamin depletion, we observed an increase in D and FMM **(Fig 2. B,C)**, indicating that disruption of heterochromatin enzymatic processes result in fragmentation of chromatin domains and loss of coherent chromatin motion."*

Ok.

6- It's very hard to compare and interpret the data in the graphs in Figure 2 (one has to keep going back and forth between different graphs). Please put them all in the same graph.

We have consolidated the data into a single graph as requested. All quantitative measurements were placed in a graph with 3x3 subpanels for easier navigation.

There are no major changes made in revised Fig 2, in fact it is almost the same as in the first version (!), so it is still very confusing. And I see no single graph or 3x3 subpanels.

7- The following sentence in line 223 needs revision in order to be understood/clear: "As removal of B- type lamins led to a significant increase in nuclear blebbing, these findings point to a bleb-associated chromatin phenotype, in which nuclear blebs contain fewer packing domains, and B-type lamin degradation could reduce the barriers for nuclear bleb formation that results from the restructuring or redistribution of packing domains".

We have modified the text as follows in lines 241-246:

© 2025. Published by The Company of Biologists under the terms of the Creative Commons Attribution License (https://creativecommons.org/licenses/by/4.0/).

“When paired with the findings in the nuclear body of each respective perturbation, this suggests a potential differential mechanism of bleb translocation for each model. Specifically, heterochromatin inhibition may be producing smaller domains that are spontaneously passing through smaller initial deformation of the nucleus. By contrast, the disruption of the lamina may be resulting in larger membrane defects that facilitate the passage of otherwise stable domains..”

Ok.

8- mClover signal is shown in figure 3 however no mention is made in the main text. Please explain and justify the use of mClover.

The use of mClover is a marker for the stable introduction of the cell line. We have modified the text to reflect that appropriately in lines 142-143.
“This cell line has an mClover fluorescent reporter fused to the lamin B1/B2 proteins that was used in our degradation experiments.”

What do the authors mean by “stable introduction of the cell line”? This makes no sense to me. Also, if the mClover is fused to the laminB1/2 proteins, why can’t we see the LaminB1/2 signal (decorating the nuclear envelope/lamina) in the DMSO panel of Fig.3? (as it nicely shown in figs. 1 and 2 of the referenced paper by the authors: *Depletion of lamins B1 and B2 promotes chromatin mobility and induces differential gene expression by a mesoscale-motion dependent mechanism, 2024)* Also, I do not know how to interpret the presence of the mClover signal and localization in the TSA- treated sample. This is all very confusing to me and will certainly be to any reader.

Additionally, please refer to the methods section of *Depletion of lamins B1 and B2 promotes chromatin mobility and induces differential gene expression by a mesoscale-motion-dependent mechanism*. Genome Biol 25, 77 (2024). https://doi.org/10.1186/s13059-024-03212-y.

9 – In fig.3A authors mention that temporal interference signal is inversely proportional to chromatin density. In line 242 authors mention that: “…within a TSA treated cell with a visually apparent bleb, chromatin decreases rapidly adjacent to the nuclear bleb…” However, the frames show no apparent changes (let alone an increase) in the temporal interference signal (which would be expected if chromatin density were to decrease).

This observation was in reference to the accompanying video with still captures of the events. We have added arrows to indicate the locations we are referring to on the respective image.

It is still very hard for me to see the changes the authors are referring to (arrows are not helping). Additionally, the authors did not address my concern regarding no apparent changes in the temporal interference signal shown in the panels (which if any, are VERY subtle). Maybe I just don’t fully understand the technique/approach used so I’m not able to interpret these panels. If this is the case, I anticipate that other readers will have the same issue and a much better job in the main text AND in the figure panels must be made.

Also, the concept of CVC should be fully explained in the main text as well as its significance and meaning (the authors simply bring it up out of the blue in the main text without any explanation of what it means and why they are looking at this parameter: “*When measuring the relative CVC change over time in individual cells, we see that there is a heterogeneity when looking at both nuclear body and bleb regions of interest (Fig. 3B)).”* Although mentioned in the *Methods* section, I could not find any reasonable explanation of the term. In fact, in the first version of the manuscript it was at least written that it means chromatin volume concentration (CVC) but now in the revised version this was fully omitted.

10- In line 245 authors say: “…evidenced by increased local chromatin density and the visual changing of density in the bleb”. I could not see or understand what the authors are referring to. Can the authors point more specifically to the referred data?

© 2025. Published by The Company of Biologists under the terms of the Creative Commons Attribution License (https://creativecommons.org/licenses/by/4.0/).

We thank the reviewer for their comment and have modified the text as follows to improve clarity in lines 265-268:
*"In the TSA treated case **(Fig. 3A bottom)**, we observe active transit of small amounts of mass between the nuclear bleb and the main nuclear body. This is evidenced by an increased local chromatin density and the temporally evolving density in the bleb **(Fig 3A TSA t:1/200-t:200/200)**."*

We have also added arrows to in the figure panel to indicate the area of interest with the referred change in density.

Again, I have the same issues as mentioned in my reply to the point above (point 9) – no apparent changes in the temporal interference signal can be observed in the panels (Fig. 3A, TSA t:1/200-t:200/200).

11- Authors should briefly explain Single Molecule Localization Microscopy and justify this approach in the main text.

We thank the reviewer for this comment and have edited the text to include this description of Single Molecule Localization Microscopy in lines 297-309:
"*Single Molecule Localization Microscopy (SMLM) is a super-resolution imaging technique that allows for the precise localization of individual fluorescent molecules, achieving spatial resolutions beyond the diffraction limit of light. This method enables detailed visualization of molecular structures and interactions within cells, providing insights into biological processes at the nanoscale. A specific version of this technique STORM (stochastic optical reconstruction microscopy) was used to image chromatin, specifically allows us to differentiate between distinct structures within a diffraction limited volume or the ensemble behavior of mass. In the manuscript, we chose to image the positioning of H3K9me3 as it's associated with high-density regions that are observed to form the centroids of domain cores on electron microscopy. By pairing this with dual-PWS microscopy, we can investigate both the ensemble behavior of chromatin throughout the nucleus and the nanoscopic structure of high-density regions formed by heterochromatin. This is achieved with its high resolution capable of resolving nanoscopic structures between 10-30nm*".

12- In line 277 authors write: "we found that the nuclear blebs arising from either B- type lamin degradation or TSA treatment contained heterochromatin around the periphery of blebs and within the center of the bleb (Fig. 4C, E, F; Fig. S2)".

1. However, the figure panels they are referring to do not match what they are saying AND there is no heterochromatin enrichment in any bleb region of TSA- treated U2OS cells.
2. Also, why there is no "Nuclear bleb" and "Nuclear body" for the Control U2OS cells in panel 4F (but there is "Nuclear bleb" and "Nuclear body" for the Control HCT116 cells in fig. S2)? Where are the graphs for auxin and GSK343 treatments for U2OS cells?
3. The scales in the graphs shown in panel 4F are different making it difficult to interpret the results. This entire figure needs a major revision to be interpretable.

We have modified the manuscript as follows:
Figure 4 and Fig. S2 has been split into two figures (Figure 4 and Figure 5). This was done to emphasize the purpose of each dataset. While both used SMLM to visualize heterochromatin in the nuclear body and blebs, the HCT116 lamin degraded dataset was used to visually confirm findings from PWS. The U2OS dataset in figure 5 was used to quantify these nanodomains statistically as the n for HCT116 cells was low. In each figure we have added arrows to clearly dictate the presence of heterochromatin both in the periphery and interior of these nuclear blebs in the lamin degradation and TSA treatment case. Quantification of these images have been placed along with the respective cell line. The manuscript text has been modified to reflect these changes between lines in the SMLM results section.

I see no arrows in the revised figure.

1. **There is no CTRL DMSO nuclear bleb and nuclear body plot as the U2OS cell line has lower frequency of blebbing and no bleb images in the DMSO case were obtained. Only TSA was shown here as this treatment has been shown (Fig. 1A) to cause the highest percentage of nuclear blebs and as such was the pharmacological agent used to**

© 2025. Published by The Company of Biologists under the terms of the Creative Commons Attribution License (https://creativecommons.org/licenses/by/4.0/).

perturb the cells and induce nuclear blebbing.

Ok.

2. **The scales are different as the number of heterochromatin localizations (H3K9me3) captured in the nuclear body vs nuclear bleb are different. The nuclear body has upwards of ~20,000 localizations while the bleb has less than ~8,000. As such, clustering algorithms will find less clusters in the bleb by default, so we chose a smaller range for our count histogram so the data can be visualized. If the same scales were used, the bleb graph would not be seen as its scale (<100) is far smaller than the nuclear body (<3500).**

Ok.

13- In line 281 authors say: "HDAC inhibition gives rise to non-canonical nuclear blebs enriched in heterochromatin around near their boundaries and the transfer of nanoscopic heterochromatin domains into the bleb". However, the data shows no heterochromatin enrichment in the bleb and it is unclear what the authors meant by "transfer of nanoscopic heterochromatin domains into the bleb" (where is the data for that?).

We thank the reviewer for this comment. The stained molecule is constitutive heterochromatin, H3K9me3, which is clearly visualized within the nuclear bleb. SMLM utilizes a time acquisition of blinking fluorophores (~10,000 frames, 30 ms exposure). This data is used to reconstruct the images shown in this figure with brighter pixels representing the presence of many localizations within that area. The inset for Fig. 5B with the added arrow shows bright areas in the bleb near the periphery representing the enrichment for heterochromatin we are referring to for the TSA treatment case.

Again, I see no arrows in the revised figure. Critically, there is no enrichment of heterochromatin in the bleb as the authors claim-the graphs clearly show this (the counts in the nuclear body are orders of magnitude higher than in the bleb). Rather, there is presence of heterochromatin in the blebs (the authors even underlines this word in point 1 above).

14- Figure legend of figure S4 is wrong and incomplete.

We apologize for the confusion, but there is no fourth figure in the supplementary figures. We have revised all legends and titles.

Ok.

15- There is no reason to have Figure 1A, B and C. Instead, they should be merged into one single graph (i.e. make Fig S1 into main figure 1).

We have merged this into one graph per the reviewer's request

Ok.

16- All the observed changes in panels 1E, F are very minimal, so one could wonder how relevant these changes are. Authors should perform WB analysis of the histone markers.

We have previously demonstrated that these histone marks are changed with WB analysis in the cell population. Western blot analysis for these treatments have been done in https://doi.org/10.1091/mbc.E17-06-0410 which corroborate our results. Further, these agents are widely used for this exact intended purpose.

Ok for WB but still, most IF quantifications shown in this manuscript show very discrete/minimal changes so it makes it difficult to assess their real contribution to the phenotypes described.

17- In line 177 authors write: Consistent with prior reports, we observe a reduction in H3K27me3 levels upon auxin-induced degradation of B-type lamins, GSK343, and TSA treatment…".

© 2025. Published by The Company of Biologists under the terms of the Creative Commons Attribution License (https://creativecommons.org/licenses/by/4.0/).

However, panel 1E shows no reduction in H3K27me3 levels upon TSA treatment (there is even a "ns" on the comparison between Control and 24hr TSA).

We apologize for this mistake; we have modified the text as follows in lines 174- 176:
"Consistent with prior reports, we observe a reduction in H3K27me3 levels upon auxin- induced degradation of B-type lamins, GSK343, however, only a concomitant increase in H3K27ac levels was observed in the auxin-treatment group ***(Fig. 1E, F).****"*

Ok.

Reviewer 2

Advance summary and potential significance to field

The topic of the manuscript is very interesting, since we still do not fully understand how the nuclear blebs are formed. The used methods are unique, and the result that nuclear blebs would contain heterochromatin is interesting.

Comments for the author

In the revised manuscript, the authors have addressed some of the concerns.
Nevertheless, the manuscript still appears premature and unpolished, it is also difficult to read and contains overinterpretation of the data. For example, there are still claims in the manuscript about significant differences, when the figure indicates that the result is not significant. It is also a pity that the authors decided not to solidify the most interesting finding, presence of hetechromatin in blebs, further.

Reviewer 3

Advance summary and potential significance to field

This paper presents significant advances in understanding the formation of nuclear blebs, particularly how chromatin structure influences this process. The authors investigate the relationship between aberrant gene expression and the mechanical properties of cell nuclei, emphasizing that the nanoscopic packing of chromatin may drive bleb formation. Using quantitative live-cell PWS-microscopy and qualitative validation by single molecule localization microscopy, they reveal distinct structural changes within nuclear blebs, suggesting that histone modifications play a key role in altering chromatin packing. This research enhances our understanding of chromatin dynamics in pathological contexts and may provide insights into cellular phenotype and function, with potential implications for diseases characterized by nuclear abnormalities.

Comments for the author

Nuclear blebs are associated with destabilized chromatin packing domains

Following are my comments on the revised manuscript which should be addressed by the authors.

Major comments:
1. Line 177-180: The following sentences should be rephrased to be clear "Consistent with prior reports, we observe a reduction in H3K27me3 levels upon auxin-induced degradation of B-type lamins, GSK343, however, only a concomitant increase in H3K27ac levels was observed in the auxin-treatment group".

It should be mentioned clearly that a reduction in H3K27me3 levels is observed upon auxin and GSK343 induced degradation of B-type lamin. What happens to H3K27ac levels with GSK343 treatment.

2. The authors have now explained the "Chromatin packing scaling D".

© 2025. Published by The Company of Biologists under the terms of the Creative Commons Attribution License (https://creativecommons.org/licenses/by/4.0/).

However, this part needs to be rephrased for clarity “As previously mentioned chromatin structure can be quantified by how its mass fills an occupied volume using the relationship between, D, known as the packing density scaling or mass fractal dimension, quantifies the relationship between the genomic length N and the radius of the occupied volume r within chromatin structures”.

3. Lines 321-322: “Visually, we observed distinct differences in H3K9me3 chromatin nanodomains (Fig. 4A) in these three conditions.” The authors should put the schematic at the end Fig. 5 to conclude their findings, and improve the schematic and explanation based on the next comment. Also, putting Fig 4A beside this sentence is misleading, as the differences in nanodomains observed in the blebs are not obvious from SMLM images. More significant differences in nanodomain clusters are observed in the nuclear body.

4. Figure 4: (i) Figure 4a: The schematic needs to be improved with effects of different treatments clearly indicated. The authors should provide a detailed explanation of the schematic in the figure legend. (ii) Figure 4 legend should contain the information that SMLM images are obtained by labeling of H3K9me3. (iii) “Red: H3K9me3, Cyan: RNAPII.” It is not clear what does the two colors mean (iv) Authors should also mention that control means DMSO treated. The % of DMSO (v/v) used in these studies should also be clearly mentioned in materials and methods.

Second revision

Author response to reviewers' comments

All changes are highlighted in the manuscript and detailed in the reviewer responses below.
Reviewer 1:
Below my comments: 1-Throughout the manuscript authors interchangeably use the terms “nuclear deformations” and “nuclear blebs” as synonyms, which is not right and makes it difficult to interpret the data. Authors should explain/define the differences of each term and use only nuclear blebs (since this is their subject of study).

We appreciate the reviewers feedback and modifying the text throughout to clarify that blebs are a subset of nuclear deformations. We then rectified the text to indicate that the measurements are focused on blebs in line 71 with “Among the most radical deformations in nuclear shape is the protrusion of chromatin from the nuclear surface, known as a nuclear bleb, that are associated with pathological transformation. [2, 6, 15, 16]”

The nuclear bleb is not necessarily a chromatin protrusion. It can also be a result of a local nuclear envelope detachment from the underlying nuclear lamina devoid of chromatin (as the lamina might still be intact). Subsequently, upon nuclear lamina disruption/rupture the chromatin can then also gain access to the bleb (see ref https://doi.org/10.1016/j.ceb.2021.01.007)

We appreciate the reviewer's comment regarding this precise point. This is a commonly held perspective within the field that nuclear blebs are not chromatin protrusions. In our accompanying manuscript, Brunner et al, we experimentally demonstrate that the hallmark of blebs/protrusions is the protrusion of chromatin and not by the disruption of the lamina. As reported in the review above, this indicates that the widely held perspective in the field needs to be revisited as these deformations are defined by the chromatin state.

2-In line 166 authors write: “…and associated with severely deformed nuclear periphery (Fig. 1B; Fig. S1A)”. However, this data is not shown in these panels nor in the paper.
We appreciate the reviewer’s careful review and apologize for this omission, we have corrected the manuscript as follows: Line 158-162: “Our results confirmed that GSK343 and TSA treatment significantly increased the percentage of blebbed nuclei within HCT-116 cells; with TSA treatment also inducing micronuclei formation and associated with a deformed nuclear periphery via increased number of blebs (Fig. 1A).”
Again, the revised data still does not show any image or quantification of micronuclei (!).

© 2025. Published by The Company of Biologists under the terms of the Creative Commons Attribution License (https://creativecommons.org/licenses/by/4.0/).

We apologize to the reviewer for omitting the quantification of the micro-nuclei from the re-submission. We have included it as a new row in supplementary table 1 which shows the average number of blebs and micronuclei across three replicates. For ease, we have attached table here as well for your reference.

SI Table 1: Average Number of Nuclei, Blebs and Micronuclei per FOV across three replicates

	Control	DMSO	24 hr. Auxin	24 hr. GSK343	24 hr. Auxin + GSK 343	24 hr. TSA	24 hr. Auxin + TSA
# of Nucelli	379.50	98.33	388.50	365.33	311.00	152.00	152.00
# Blebs	7.00	1.33	21.00	24.33	25.33	26.33	37.33
# Micro-nuclei	3	0.667	18.5	9.333	9.667	12.000	13.000

6-It's very hard to compare and interpret the data in the graphs in Figure 2 (one has to keep going back and forth between different graphs). Please put them all in the same graph.

We have consolidated the data into a single graph as requested. All quantitative measurements were placed in a graph with 3x3 subpanels for easier navigation.

There are no major changes made in revised Fig 2, in fact it is almost the same as in the first version (!), so it is still very confusing. And I see no single graph or 3x3 subpanels.

We apologize for the miscommunication in how we described the changes to figure 2. We reorganized the figures for clarity by repositioning all the quantitative parameters measured (D, FMM, Effective Diffusion coefficient) on one side of the figure in a ***3x2 organization****. We believe that this separation is clearer since the images for both treatment types: the auxin treatment in HCT116 LMNBB1&B2-AID cells and the GSK343, TSA treated cells are separated from the data. In reviewing the results, we find that separating the conditions makes it clearer and avoids crowded graphs.*

8-mClover signal is shown in figure 3 however no mention is made in the main text. Please explain and justify the use of mClover.

The use of mClover is a marker for the stable introduction of the cell line. We have modified the text to reflect that appropriately in lines 142-143. "This cell line has an mClover fluorescent reporter fused to the lamin B1/B2 proteins that was used in our degradation experiments."

What do the authors mean by "stable introduction of the cell line"? This makes no sense to me. Also, if the mClover is fused to the laminB1/2 proteins, why can't we see the LaminB1/2 signal (decorating the nuclear envelope/lamina) in the DMSO panel of Fig.3? (as it nicely shown in figs. 1 and 2 of the referenced paper by the authors: Depletion of lamins B1 and B2 promotes chromatin mobility and induces differential gene expression by a mesoscale-motion dependent mechanism, 2024)

Also, I do not know how to interpret the presence of the mClover signal and localization in the TSA treated sample. This is all very confusing to me and will certainly be to any reader.

The HCT116 cell line was genetically engineered to constitutively express mClover fluorescent protein fused to lamin B1/B2. This genetic modification ***was stably integrated*** *into the genome, creating a cell line that permanently expresses the fluorescent reporter across cell generations. Hence we replied to the first reviewer comments with mClover is a marker for the stable introduction of the AID system.*

We would like to clarify to the reviewer that the imaging modality used in this manuscript's figure 1, and the referred manuscript (Depletion of lamins B1 and B2 promotes chromatin mobility and induces differential gene expression by a mesoscale-motion dependent mechanism, 2024) is different from that used in figure 3. Figure 1 and the reference use confocal imaging while the fluorescent images in figure 3 are taken on the PWS imaging system which uses a Liquid Crystal Tunable Filter (LCTF). This component performs the spectral discrimination needed by PWS. The LCTF has a narrow passband which causes a weaker SNR, as such the enriched borders seen in

© 2025. Published by The Company of Biologists under the terms of the Creative Commons Attribution License (https://creativecommons.org/licenses/by/4.0/).

figure 1and in the referenced manuscript are not present here. We recognize that this is not clear and have updated the legend to reflect this difference.
Lastly we would like to clarify to the reviewer that the cells in this figure were treated with TSA not auxin so there is no lamin degradation. The label on the images signifies that these are the stable AID cell lines used throughout the experiment. We clarified and emphasized in the legend to avoid reader confusion.
9 – In fig.3A authors mention that temporal interference signal is inversely proportional to chromatin density. In line 242 authors mention that: "…within a TSA treated cell with a visually apparent bleb, chromatin decreases rapidly adjacent to the nuclear bleb…" However, the frames show no apparent changes (let alone an increase) in the temporal interference signal (which would be expected if chromatin density were to decrease).
This observation was in reference to the accompanying video with still captures of the events. We have added arrows to indicate the locations we are referring to on the respective image.

1. It is still very hard for me to see the changes the authors are referring to (arrows are not helping).
2. Additionally, the authors did not address my concern regarding no apparent changes in the temporal interference signal shown in the panels (which if any, are VERY subtle). Maybe I just don't fully understand the technique/approach used so I'm not able to interpret these panels. If this is the case, I anticipate that other readers will have the same issue and a much better job in the main text AND in the figure panels must be made.
3. Also, the concept of CVC should be fully explained in the main text as well as its significance and meaning (the authors simply bring it up out of the blue in the main text without any explanation of what it means and why they are looking at this parameter: "When measuring the relative CVC change over time in individual cells, we see that there is a heterogeneity when looking at both nuclear body and bleb regions of interest (Fig. 3B))." Although mentioned in the Methods section, I could not find any reasonable explanation of the term. In fact, in the first version of the manuscript it was at least written that it means chromatin volume concentration (CVC) but now in the revised version this was fully omitted.

1. *We apologize to the reviewer if the changes are not clear in our last revision. Firstly, we would like to draw your attention to the cropped image below. The yellow arrow is supposed to point towards the region of the nucleus near the bleb which shows a change in the signal. We have selected the best cell and time to show case these differences.*

2. [illegible]

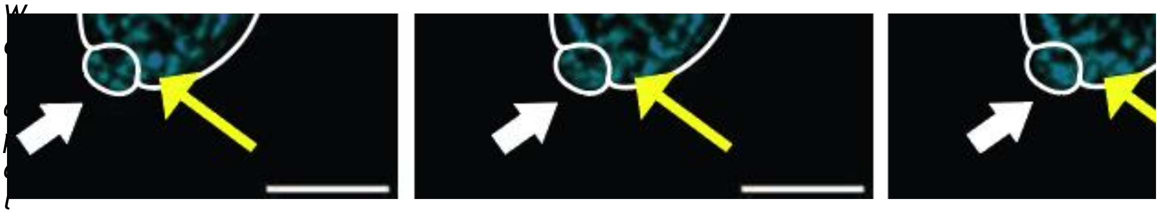

ogize for the difficulty in interpreting these figures. To facilitate understanding, we have added two diagrams that visually show examples of two nuclear CVC's so readers can see how these changes are physically. These diagrams have been added as panels to figure 3.

3. *We apologize for the lack of explanation in the resubmission, we have added a brief description of CVC when it is first introduced in the results section for figure 3 and point the reader to the methods section. Please see the description that we have added below.*

As discussed above, variations in the temporal interference quantifies the FMM while the temporal-average signal is inversely proportional to the chromatin volume concentration (chromatin density) and the density of other nuclear macromolecules.[39, 41]. Chromatin volume concentration (CVC), as the name implies, describes how chromatin occupies the volume on a relative scale of 0 to 1. Two examples of relative CVC that are seen in our analysis are illustrated in Fig. 3A

10-In line 245 authors say: "…evidenced by increased local chromatin density and the visual changing of density in the bleb". I could not see or understand what the authors are referring to. Can the authors point more specifically to the referred data?

© 2025. Published by The Company of Biologists under the terms of the Creative Commons Attribution License (https://creativecommons.org/licenses/by/4.0/).

We thank the reviewer for their comment and have modified the text as follows to improve clarity in lines 265-268: "In the TSA treated case (Fig. 3A bottom), we observe active transit of small amounts of mass between the nuclear bleb and the main nuclear body. This is evidenced by an increased local chromatin density and the temporally evolving density in the bleb (Fig 3A TSA t:1/200-t:200/200)." We have also added arrows to the figure panel to indicate the area of interest with the referred change in density.
Again, I have the same issues as mentioned in my reply to the point above (point 9) – no apparent changes in the temporal interference signal can be observed in the panels (Fig. 3A, TSA t:1/200-t:200/200).
We appreciate that the differences may be visually subtle but the quantification for an example cell treated with TSA (Fig. 3C) and ensemble averages (Fig. 3D) are shown.

12-In line 277 authors write: "we found that the nuclear blebs arising from either B type lamin degradation or TSA treatment contained heterochromatin around the periphery of blebs and within the center of the bleb (Fig. 4C, E, F; Fig. S2)".

1. However, the figure panels they are referring to do not match what they are saying AND there is no heterochromatin enrichment in any bleb region of TSA treated U2OS cells.
2. Also, why there is no "Nuclear bleb" and "Nuclear body" for the Control U2OS cells in panel 4F (but there is "Nuclear bleb" and "Nuclear body" for the Control HCT116 cells in fig. S2)? Where are the graphs for auxin and GSK343 treatments for U2OS cells?
3. The scales in the graphs shown in panel 4F are different making it difficult to interpret the results. This entire figure needs a major revision to be interpretable.

I see no arrows in the revised figure
We apologize to the reviewer for the oversight. The arrows have now been incorporated into figure 4.

13-In line 281 authors say: "HDAC inhibition gives rise to non-canonical nuclear blebs enriched in heterochromatin around near their boundaries and the transfer of nanoscopic heterochromatin domains into the bleb". However, the data shows no heterochromatin enrichment in the bleb and it is unclear what the authors meant by "transfer of nanoscopic heterochromatin domains into the bleb" (where is the data for that?).
We thank the reviewer for this comment. The stained molecule is constitutive heterochromatin, H3K9me3, which is clearly visualized within the nuclear bleb. SMLM utilizes a time acquisition of blinking fluorophores (~10,000 frames, 30 ms exposure). This data is used to reconstruct the images shown in this figure with brighter pixels representing the presence of many localizations within that area. The inset for Fig. 5B with the added arrow shows bright areas in the bleb near the periphery representing the <u>enrichment for heterochromatin</u> we are referring to for the TSA treatment case.
Again, I see no arrows in the revised figure. Critically, there is no enrichment of heterochromatin in the bleb as the authors claim-the graphs clearly show this (the counts in the nuclear body are orders of magnitude higher than in the bleb). Rather, there is presence of heterochromatin in the blebs (the authors even underlines this word in point 1 above).
We apologize to the reviewer for this oversight. We have added the arrows that were intended to show the presence of heterochromatin (yellow signal) in the nuclear bleb. We agree that the use of enrichment in our first response is misleading. We have ensured that there is no mention of enrichment in the main manuscript and only highlight the ***presence*** *of heterochromatin in the nuclear bleb. Our use of enrichment was in reference to the bright borders of the nuclear envelope in the bleb but appreciate the reviewer for expressing the confusion.*
The quantification in this figure is not a general measure of heterochromatin, instead it is quantifying the number of heterochromatic clusters that have been identified by our analysis algorithm The nuclear body and bleb are not expected to share the same magnitude, as the body contains a greater proportion of the genome. The body and bleb should not be compared to each other, rather they should be considered separately and compared across treatments. We reviewed the figure legend and the manuscript to make sure this is clear.

16-All the observed changes in panels 1E, F are very minimal, so one could wonder how relevant these changes are. Authors should perform WB analysis of the histone markers
We have previously demonstrated that these histone marks are changed with WB analysis in the cell population. Western blot analysis for these treatments have been done in

© 2025. Published by The Company of Biologists under the terms of the Creative Commons Attribution License (https://creativecommons.org/licenses/by/4.0/).

https://doi.org/10.1091/mbc.E17-06-0410 which corroborate our results. Further, these agents are widely used for this exact intended purpose.

Ok for WB but still, most IF quantifications shown in this manuscript show very discrete/minimal changes so it makes it difficult to assess their real contribution to the phenotypes described.

We appreciate the reviewer's comment on the IF quantification. In figure 1, we show plots C and D which demonstrate a change in the distribution of corrected total fluorescent signal of H3K27me3 and H3K27ac respectively for different conditions. As stated in the manuscript, these changes were statistically tested via a One-way ANOVA, and multiple comparisons were done via Dunnett's test. Within the main text, we only note the statistically different groups thus only highlighting the treatments that were contributing to phenotypes we are describing.

Reviewer 2:

In the revised manuscript, the authors have addressed some of the concerns. Nevertheless, the manuscript still appears premature and unpolished, it is also difficult to read and contains overinterpretation of the data. For example, there are still claims in the manuscript about significant differences, when the figure indicates that the result is not significant. It is also a pity that the authors decided not to solidify the most interesting finding, presence of heterochromatin in blebs, further.

We thank the reviewer for their comment. With regards to overinterpretation of the data, we have double checked our manuscript to ensure our claims are in line with the statistical testing done. As stated for a reviewer 1 comment, our discussion of IF data of H3K27me3 and H3K27ac only speaks to the differences that were found to be significantly different (Auxin and GSK343 treatment).

For figure 2, it might seem that the differences are numerically minute. For example, in figure 2 subpanel B, the mean for control to treated goes from ~2.6 to 2.7, but these small changes have a large effect on volume as chromatin organization follows a power law behavior not a linear one. Analysis of PWS data should be done with this consideration as values will range from 2.2 to 2.8 and the volume follows r^D as such small changes have considerable effects.

In this manuscript, we reported finding heterochromatin in the nuclear blebs and have characterized the number and size of the heterochromatin nanodomains as identified by our analysis pipeline. There are nuances in the type of heterochromatin as evidenced by changes seen in IF for H3K27me3, as such additional work is required to explore these nuances further.

Reviewer 3:

Nuclear blebs are associated with destabilized chromatin packing domains. Following are my comments on the revised manuscript which should be addressed by the authors.

Major comments:

1. Line 177-180: The following sentences should be rephrased to be clear "Consistent with prior reports, we observe a reduction in H3K27me3 levels upon auxin-induced degradation of B-type lamins, GSK343, however, only a concomitant increase in H3K27ac levels was observed in the auxin-treatment group". It should be mentioned clearly that a reduction in H3K27me3 levels is observed upon auxin and GSK343 induced degradation of B-type lamin. What happens to H3K27ac levels with GSK343 treatment.

We apologize to the reviewer for the confusion. We have re-phrased the sentence as follows.

"Consistent with prior reports, we observe a reduction in H3K27me3 levels upon auxin-induced degradation of B-type lamins and GSK343 treatment respectively. For H3K27ac, we observe an increase in levels in the auxin-treatment group and GSK343 treatment respectively. **(Fig. 1C, D).**"

As for H3K27ac, we explicitly state the effect in the sentence to avoid any lack of clarity.

2. The authors have now explained the "Chromatin packing scaling D". However, this part needs to be rephrased for clarity "As previously mentioned, chromatin structure can be quantified by how its mass fills an occupied volume using the relationship between, D, known as the packing density scaling or mass fractal dimension, quantifies the relationship between the genomic length N and the radius of the occupied volume r within chromatin structures".

We apologize for the confusing wording; we have changed the wording to have a clearer description of the parameter.

"The relationship between genomic length (N) and radius (r) can be used to quantify how chromatin is packed within a given volume. This relationship is characterized by the mass fractal dimension (D), which describes how densely the chromatin mass fills the space it occupies."

© 2025. Published by The Company of Biologists under the terms of the Creative Commons Attribution License (https://creativecommons.org/licenses/by/4.0/).

3. Lines 321-322: “Visually, we observed distinct differences in H3K9me3 chromatin nanodomains (Fig. 4A) in these three conditions.” The authors should put the schematic at the end Fig. 5 to conclude their findings, and improve the schematic and explanation based on the next comment. Also, putting Fig 4A beside this sentence is misleading, as the differences in nanodomains observed in the blebs are not obvious from SMLM images. More significant differences in nanodomain clusters are observed in the nuclear body.
We appreciate the reviewer’s comment suggesting inclusion of other perturbations and their resultant effect on the domains. We have added a new version of the figure that includes these in Figure 4A.

4. Figure 4: (i) Figure 4a: The schematic needs to be improved with effects of different treatments clearly indicated. The authors should provide a detailed explanation of the schematic in the figure legend. (ii) Figure 4 legend should
contain the information that SMLM images are obtained by labeling of H3K9me3.
(iii) “Red: H3K9me3, Cyan: RNAPII.” It is not clear what does the two colors
mean (iv) Authors should also mention that control means DMSO treated. The % of
DMSO (v/v) used in these studies should also be clearly mentioned in materials
and methods.
We apologize to the reviewer for the incorrect description of the figure. We have removed the sentence and have added the arrow that was stated to be present. We also have detailed DMSO concentration in the figure legend. And materials and methods.

Third decision letter

MS ID#: JOCES/2024/262161

MS TITLE: Nuclear blebs are associated with destabilized chromatin packing domains

AUTHORS: Emily M Pujadas Liwag, Nicolas Acosta, Luay Matthew Almassalha, Yuanzhe (Patrick) Su, Ruyi Gong, Masato T Kanemaki, Andrew D Stephens, and Vadim Backman

I am happy to tell you that your manuscript has been accepted for publication in Journal of Cell Science, pending standard publication integrity checks. As you will see, the referees still have some minor suggestions for further improving your manuscript's readability. Please incorporate these small changes when possible.

Reviewer 1

Advance summary and potential significance to field

My comments are written in green.

Comments for the author

Reviewer 1:

Below my comments: 1-Throughout the manuscript authors interchangeably use the terms “nuclear deformations” and “nuclear blebs” as synonyms, which is not right and makes it difficult to interpret the data. Authors should explain/define the differences of each term and use only nuclear blebs (since this is their subject of study).

We appreciate the reviewers feedback and modifying the text throughout to clarify that blebs are a subset of nuclear deformations. We then rectified the text to indicate that the measurements are focused on blebs in line 71 with “Among the most radical deformations in nuclear shape is the protrusion of chromatin from the nuclear surface, known as a nuclear

© 2025. Published by The Company of Biologists under the terms of the Creative Commons Attribution License (https://creativecommons.org/licenses/by/4.0/).

bleb, that are associated with pathological transformation. [2, 6, 15, 16]"

The nuclear bleb is not necessarily a chromatin protrusion. It can also be a result of a local nuclear envelope detachment from the underlying nuclear lamina devoid of chromatin (as the lamina might still be intact). Subsequently, upon nuclear lamina disruption/rupture the chromatin can then also gain access to the bleb (see ref https://doi.org/10.1016/j.ceb.2021.01.007)

We appreciate the reviewer's comment regarding this precise point. This is a commonly held perspective within the field that nuclear blebs are not chromatin protrusions. In our accompanying manuscript, Brunner et al, we experimentally demonstrate that the hallmark of blebs/protrusions is the protrusion of chromatin and not by the disruption of the lamina. As reported in the review above, this indicates that the widely held perspective in the field needs to be revisited as these deformations are defined by the chromatin state.

Thank you for the explanation as I did not know this paper. However, the cited paper does not address whether it has to occur a breach/rupture in the nuclear lamina in order to allow for chromatin to enter into the bleb. So, the question remains: Can a bleb be devoid of chromatin (as the lamina might still be intact and thus prevent chromatin from entering the bleb)? Or perhaps could the chromatin simply push the lamina into the bleb without breaking it? The fact the Lamin B1 is drastically different in blebs in comparison to the primary nucleus does not help us answering these questions which I must confess have been intriguing me for a long while!

2-In line 166 authors write: "…and associated with severely deformed nuclear periphery (Fig. 1B; Fig. S1A)". However, this data is not shown in these panels nor in the paper.

We appreciate the reviewer's careful review and apologize for this omission, we have corrected the manuscript as follows: Line 158-162: "Our results confirmed that GSK343 and TSA treatment significantly increased the percentage of blebbed nuclei within HCT-116 cells; with TSA treatment also inducing micronuclei formation and associated with a deformed nuclear periphery via increased number of blebs (Fig. 1A)."

Again, the revised data still does not show any image or quantification of micronuclei (!).

We apologize to the reviewer for omitting the quantification of the micro-nuclei from the re-submission. We have included it as a new row in supplementary table 1 which shows the average number of blebs and micronuclei across three replicates. For ease, we have attached table here as well for your reference.

SI Table 1: Average Number of Nuclei, Blebs and Micronuclei per FOV across three replicates

	Control	DMSO	24 hr. Auxin	24 hr. GSK343	24 hr. Auxin + GSK 343	24 hr. TSA	24 hr. Auxin + TSA
# of Nucelli	379.50	98.33	388.50	365.33	311.00	152.00	152.00
# Blebs	7.00	1.33	21.00	24.33	25.33	26.33	37.33
# Micro-nuclei	3	0.667	18.5	9.333	9.667	12.000	13.000

OK!

© 2025. Published by The Company of Biologists under the terms of the Creative Commons Attribution License (https://creativecommons.org/licenses/by/4.0/).

6-It's very hard to compare and interpret the data in the graphs in Figure 2 (one has to keep going back and forth between different graphs). Please put them all in the same graph.

We have consolidated the data into a single graph as requested. All quantitative measurements were placed in a graph with 3x3 subpanels for easier navigation.

There are no major changes made in revised Fig 2, in fact it is almost the same as in the first version (!), so it is still very confusing. And I see no single graph or 3x3 subpanels.

*We apologize for the miscommunication in how we described the changes to figure 2. We reorganized the figures for clarity by repositioning all the quantitative parameters measured (D, FMM, Effective Diffusion coefficient) on one side of the figure in a **3x2 organization**. We believe that this separation is clearer since the images for both treatment types: the auxin treatment in HCT116 LMNBB1&B2-AID cells and the GSK343, TSA treated cells are separated from the data. In reviewing the results, we find that separating the conditions makes it clearer and avoids crowded graphs.*

OK!

8-mClover signal is shown in figure 3 however no mention is made in the main text. Please explain and justify the use of mClover.

The use of mClover is a marker for the stable introduction of the cell line. We have modified the text to reflect that appropriately in lines 142-143. "This cell line has an mClover fluorescent reporter fused to the lamin B1/B2 proteins that was used in our degradation experiments."

What do the authors mean by "stable introduction of the cell line"? This makes no sense to me. Also, if the mClover is fused to the laminB1/2 proteins, why can't we see the LaminB1/2 signal (decorating the nuclear envelope/lamina) in the DMSO panel of Fig.3? (as it nicely shown in figs. 1 and 2 of the referenced paper by the authors: Depletion of lamins B1 and B2 promotes chromatin mobility and induces differential gene expression by a mesoscale-motion dependent mechanism, 2024).

Also, I do not know how to interpret the presence of the mClover signal and localization in the TSA treated sample. This is all very confusing to me and will certainly be to any reader.

*The HCT116 cell line was genetically engineered to constitutively express mClover fluorescent protein fused to lamin B1/B2. This genetic modification **was stably integrated** into the genome, creating a cell line that permanently expresses the fluorescent reporter across cell generations. Hence we replied to the first reviewer comments with mClover is a marker for the stable introduction of the AID system.*

OK, but please be more clear as "stable introduction of the cell line" is not a term used to indicate the generation of a stable cell line. That's why I was confused.

We would like to clarify to the reviewer that the imaging modality used in this manuscript's figure 1, and the referred manuscript (Depletion of lamins B1 and B2 promotes chromatin mobility and induces differential gene expression by a mesoscale- motion dependent mechanism, 2024) is different from that used in figure 3. Figure 1 and the reference use confocal imaging while the fluorescent images in figure 3 are taken on the PWS imaging system which uses a Liquid Crystal Tunable Filter (LCTF). This component performs the spectral discrimination needed by PWS. The LCTF has a narrow passband which causes a weaker SNR, as such the enriched borders seen in figure 1and in the referenced manuscript are not present here. We recognize that this is not clear and have updated the legend to reflect this difference.

OK, but can the authors please include to the figure legend the exact explanation above. I think this will be helpful to the readers.

© 2025. Published by The Company of Biologists under the terms of the Creative Commons Attribution License (https://creativecommons.org/licenses/by/4.0/).

Lastly, we would like to clarify to the reviewer that the cells in this figure were treated with TSA not auxin so there is no lamin degradation. The label on the images signifies that these are the stable AID cell lines used throughout the experiment. We clarified and emphasized in the legend to avoid reader confusion.

I got that the cells were not treated with auxin. I just did not know if there was some meaning/result/message being conveyed by the authors regarding the distribution of the mClover signal in this sample since there is virtually no signal in the DMSO sample.

9 – In fig.3A authors mention that temporal interference signal is inversely proportional to chromatin density. In line 242 authors mention that: "…within a TSA treated cell with a visually apparent bleb, chromatin decreases rapidly adjacent to the nuclear bleb…" However, the frames show no apparent changes (let alone an increase) in the temporal interference signal (which would be expected if chromatin density were to decrease).

This observation was in reference to the accompanying video with still captures of the events. We have added arrows to indicate the locations we are referring to on the respective image.

1. It is still very hard for me to see the changes the authors are referring to (arrows are not helping).
2. Additionally, the authors did not address my concern regarding no apparent changes in the temporal interference signal shown in the panels (which if any, are VERY subtle). Maybe I just don't fully understand the technique/approach used so I'm not able to interpret these panels. If this is the case, I anticipate that other readers will have the same issue and a much better job in the main text AND in the figure panels must be made.
3. Also, the concept of CVC should be fully explained in the main text as well as its significance and meaning (the authors simply bring it up out of the blue in the main text without any explanation of what it means and why they are looking at this parameter: "When measuring the relative CVC change over time in individual cells, we see that there is a heterogeneity when looking at both nuclear body and bleb regions of interest (Fig. 3B))." Although mentioned in the Methods section, I could not find any reasonable explanation of the term. In fact, in the first version of the manuscript it was at least written that it means chromatin volume concentration (CVC) but now in the revised version this was fully omitted.

1. *We apologize to the reviewer if the changes are not clear in our last revision. Firstly, we would like to draw your attention to the cropped image below. The yellow arrow is supposed to point towards the region of the nucleus near the bleb which shows a change in the signal. We have selected the best cell and time to show case these differences.*

   OK! I guess what caused confusion was the fact that I'm not used to "read" this type of data. The authors should be more didactic when mentioning this specific result in the main text.

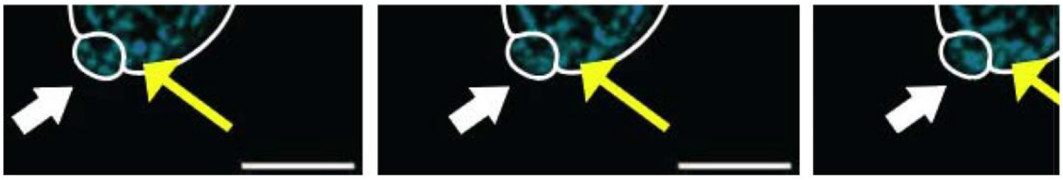

2. We *apologize for the difficulty in interpreting these figures. To facilitate understanding, we have added two diagrams that visually show examples of two nuclear CVC's so readers can see how these changes are physically. These diagrams have been added as panels to figure 3.*

© 2025. Published by The Company of Biologists under the terms of the Creative Commons Attribution License (https://creativecommons.org/licenses/by/4.0/).

Please add graph keys to both graphs in Fig 3B to indicate that blue and orange curves represent blebs and nuclear body respectively, just as it was done for panel 3C (actually authors should make the colors the same for graphs in figure 3B and 3C to be consistent).

3. *We apologize for the lack of explanation in the resubmission, we have added a brief description of CVC when it is first introduced in the results section for figure 3 and point the reader to the methods section. Please see the description that we have added below.*

   As discussed above, variations in the temporal interference quantifies the FMM while the temporal-average signal is inversely proportional to the chromatin volume concentration (chromatin density) and the density of other nuclear macromolecules.[39, 41]. Chromatin volume concentration (CVC), as the name implies, describes how chromatin occupies the volume on a relative scale of 0 to 1. Two examples of relative CVC that are seen in our analysis are illustrated in Fig. 3A

   OK!

10-In line 245 authors say: "…evidenced by increased local chromatin density and the visual changing of density in the bleb". I could not see or understand what the authors are referring to. Can the authors point more specifically to the referred data?

We thank the reviewer for their comment and have modified the text as follows to improve clarity in lines 265-268: "In the TSA treated case (Fig. 3A bottom), we observe active transit of small amounts of mass between the nuclear bleb and the main nuclear body. This is evidenced by an increased local chromatin density and the temporally evolving density in the bleb (Fig 3A TSA t:1/200-t:200/200)." We have also added arrows to the figure panel to indicate the area of interest with the referred change in density.

Again, I have the same issues as mentioned in my reply to the point above (point 9) – no apparent changes in the temporal interference signal can be observed in the panels (Fig. 3A, TSA t:1/200-t:200/200).

We appreciate that the differences may be visually subtle but the quantification for an example cell treated with TSA (Fig. 3C) and ensemble averages (Fig. 3D) are shown.

OK! And again: I guess what caused confusion was the fact that I'm not used to "read" this type of data-despite having a deep knowledge on microscopy techniques. Thus, the authors should be more didactic when mentioning this specific result in the main text for a broader audience.

12-In line 277 authors write: "we found that the nuclear blebs arising from either B type lamin degradation or TSA treatment contained heterochromatin around the periphery of blebs and within the center of the bleb (Fig. 4C, E, F; Fig. S2)".

1. However, the figure panels they are referring to do not match what they are saying AND there is no heterochromatin enrichment in any bleb region of TSA treated U2OS cells.
2. Also, why there is no "Nuclear bleb" and "Nuclear body" for the Control U2OS cells in panel 4F (but there is "Nuclear bleb" and "Nuclear body" for the Control HCT116 cells in fig. S2)? Where are the graphs for auxin and GSK343 treatments for U2OS cells?
3. The scales in the graphs shown in panel 4F are different making it difficult to interpret the results. This entire figure needs a major revision to be interpretable.

I see no arrows in the revised figure.

© 2025. Published by The Company of Biologists under the terms of the Creative Commons Attribution License (https://creativecommons.org/licenses/by/4.0/).

We apologize to the reviewer for the oversight. The arrows have now been incorporated into figure 4.

OK!

13-In line 281 authors say: "HDAC inhibition gives rise to non-canonical nuclear blebs enriched in heterochromatin around near their boundaries and the transfer of nanoscopic heterochromatin domains into the bleb". However, the data shows no heterochromatin enrichment in the bleb and it is unclear what the authors meant by "transfer of nanoscopic heterochromatin domains into the bleb" (where is the data for that?).

We thank the reviewer for this comment. The stained molecule is constitutive heterochromatin, H3K9me3, which is clearly visualized within the nuclear bleb. SMLM utilizes a time acquisition of blinking fluorophores (~10,000 frames, 30 ms exposure). This data is used to reconstruct the images shown in this figure with brighter pixels representing the presence of many localizations within that area. The inset for Fig. 5B with the added arrow shows bright areas in the bleb near the periphery representing the enrichment for heterochromatin we are referring to for the TSA treatment case.

Again, I see no arrows in the revised figure. Critically, there is no enrichment of heterochromatin in the bleb as the authors claim-the graphs clearly show this (the counts in the nuclear body are orders of magnitude higher than in the bleb). Rather, there is presence of heterochromatin in the blebs (the authors even underline this word in point 1 above).

*We apologize to the reviewer for this oversight. We have added the arrows that were intended to show the presence of heterochromatin (yellow signal) in the nuclear bleb. We agree that the use of enrichment in our first response is misleading. We have ensured that there is no mention of enrichment in the main manuscript and only highlight the **presence** of heterochromatin in the nuclear bleb. Our use of enrichment was in reference to the bright borders of the nuclear envelope in the bleb but appreciate the reviewer for expressing the confusion.*

Although the authors claim that they added the arrows-I still cannot see any arrows (!)- unless I downloaded the wrong updated figure. Of note, the same happened in the second round of revisions (when authors claimed they had added arrows in fig. 4 and micronuclei quantification to fig. 1, but nothing was added-only in this latest version after I again pointed that these were missing, they were finally added). In my opinion, this shows that the authors are not being careful enough when revising their manuscript as this is already the 3 round of revisions and the same problem persists.

The quantification in this figure is not a general measure of heterochromatin, instead it is quantifying the number of heterochromatic clusters that have been identified by our analysis algorithm. The nuclear body and bleb are not expected to share the same magnitude, as the body contains a greater proportion of the genome. The body and bleb should not be compared to each other, rather they should be considered separately and compared across treatments. We reviewed the figure legend and the manuscript to make sure this is clear.

OK!

16-All the observed changes in panels 1E, F are very minimal, so one could wonder how relevant these changes are. Authors should perform WB analysis of the histone markers

We have previously demonstrated that these histone marks are changed with WB analysis in the cell population. Western blot analysis for these treatments have been done in https://doi.org/10.1091/mbc.E17-06-0410 which corroborate our results. Further, these agents are widely used for this exact intended purpose.

Ok for WB but still, most IF quantifications shown in this manuscript show very discrete/minimal changes so it makes it difficult to assess their real contribution to the phenotypes described.

© 2025. Published by The Company of Biologists under the terms of the Creative Commons Attribution License (https://creativecommons.org/licenses/by/4.0/).

We appreciate the reviewer's comment on the IF quantification. In figure 1, we show plots C and D which demonstrate a change in the distribution of corrected total fluorescent signal of H3K27me3 and H3K27ac respectively for different conditions. As stated in the manuscript, these changes were statistically tested via a One-way ANOVA, and multiple comparisons were done via Dunnett's test. Within the main text, we only note the statistically different groups thus only highlighting the treatments that were contributing to phenotypes we are describing.

OK!

Reviewer 2:

In the revised manuscript, the authors have addressed some of the concerns. Nevertheless, the manuscript still appears premature and unpolished, it is also difficult to read and contains overinterpretation of the data. For example, there are still claims in the manuscript about significant differences, when the figure indicates that the result is not significant. It is also a pity that the authors decided not to solidify the most interesting finding, presence of heterochromatin in blebs, further.

We thank the reviewer for their comment. With regards to overinterpretation of the data, we have double checked our manuscript to ensure our claims are in line with the statistical testing done. As stated for a reviewer 1 comment, our discussion of IF data of H3K27me3 and H3K27ac only speaks to the differences that were found to be significantly different (Auxin and GSK343 treatment).

For figure 2, it might seem that the differences are numerically minute. For example, in figure 2 subpanel B, the mean for control to treated goes from ~2.6 to 2.7, but these small changes have a large effect on volume as chromatin organization follows a power law behavior not a linear one. Analysis of PWS data should be done with this consideration as values will range from 2.2 to 2.8 and the volume follows r^D *as such small changes have considerable effects.*

In this manuscript, we reported finding heterochromatin in the nuclear blebs and have characterized the number and size of the heterochromatin nanodomains as identified by our analysis pipeline. There are nuances in the type of heterochromatin as evidenced by changes seen in IF for H3K27me3, as such additional work is required to explore these nuances further.

Reviewer 3:

Nuclear blebs are associated with destabilized chromatin packing domains. Following are my comments on the revised manuscript which should be addressed by the authors.

Major comments:

1. Line 177-180: The following sentences should be rephrased to be clear "Consistent with prior reports, we observe a reduction in H3K27me3 levels upon auxin- induced degradation of B-type lamins, GSK343, however, only a concomitant increase in H3K27ac levels was observed in the auxin-treatment group". It should be mentioned clearly that a reduction in H3K27me3 levels is observed upon auxin and GSK343 induced degradation of B-type lamin. What happens to H3K27ac levels with GSK343 treatment.

We apologize to the reviewer for the confusion. We have re-phrased the sentence as follows.

"Consistent with prior reports, we observe a reduction in H3K27me3 levels upon auxin-induced degradation of B-type lamins and GSK343 treatment respectively. For H3K27ac, we observe an increase in levels in the auxin-treatment group and GSK343 treatment respectively. **(Fig. 1C, D).**"

As for H3K27ac, we explicitly state the effect in the sentence to avoid any lack of clarity.

2. The authors have now explained the "Chromatin packing scaling D". However, this part

© 2025. Published by The Company of Biologists under the terms of the Creative Commons Attribution License (https://creativecommons.org/licenses/by/4.0/).

needs to be rephrased for clarity “As previously mentioned, chromatin structure can be quantified by how its mass fills an occupied volume using the relationship between, D, known as the packing density scaling or mass fractal dimension, quantifies the relationship between the genomic length N and the radius of the occupied volume r within chromatin structures”.

We apologize for the confusing wording; we have changed the wording to have a clearer description of the parameter.

“The relationship between genomic length (N) and radius (r) can be used to quantify how chromatin is packed within a given volume. This relationship is characterized by the mass fractal dimension (D), which describes how densely the chromatin mass fills the space it occupies.”

3. Lines 321-322: “Visually, we observed distinct differences in H3K9me3 chromatin nanodomains (Fig. 4A) in these three conditions.” The authors should put the schematic at the end Fig. 5 to conclude their findings, and improve the schematic and explanation based on the next comment. Also, putting Fig 4A beside this sentence is misleading, as the differences in nanodomains observed in the blebs are not obvious from SMLM images. More significant differences in nanodomain clusters are observed in the nuclear body.

We appreciate the reviewer’s comment suggesting inclusion of other perturbations and their resultant effect on the domains. We have added a new version of the figure that includes these in Figure 4A.

4. Figure 4: (i) Figure 4a: The schematic needs to be improved with effects of different treatments clearly indicated. The authors should provide a detailed explanation of the schematic in the figure legend. (ii) Figure 4 legend should contain the information that SMLM images are obtained by labeling of H3K9me3.
(iii) “Red: H3K9me3, Cyan: RNAPII.” It is not clear what does the two colors mean (iv) Authors should also mention that control means DMSO treated. The % of DMSO (v/v) used in these studies should also be clearly mentioned in materials and methods.

We apologize to the reviewer for the incorrect description of the figure. We have removed the sentence and have added the arrow that was stated to be present. We also have detailed DMSO concentration in the figure legend. And materials and methods.

<u>Reviewer 3</u>

Advance summary and potential significance to field

The manuscript investigates the dynamics and organization of heterochromatin nanodomains in the nucleus and nuclear blebs induced by heterochromatin loss and b-type lamin depletion. This study employs live-cell PWS microscopy, complemented by super-resolution imaging, to validate the findings. It explores a highly novel and biologically significant area with implications for both physiological and pathological conditions, utilizing an advanced and emerging imaging technique.

Comments for the author

The authors have made thorough revisions to the manuscript and adequately addressed all comments and recommendations provided earlier. One minor comment: “An equivalent final concentration of DMSO in media was used for the control group.” The authors should rephrase this statement and mention the exact amount of DMSO added in volume/volume units(v/v). Given that DMSO can influence cell viability and experimental outcomes even at low concentrations, providing precise details is critical for reproducibility and for evaluating whether the DMSO levels used are within a non-toxic range. After addressing this minor comment, the manuscript is suitable for publication."

© 2025. Published by The Company of Biologists under the terms of the Creative Commons Attribution License (https://creativecommons.org/licenses/by/4.0/).

Final Response to Reviewers

Author response to reviewers' comments

All changes are highlighted in the manuscript and detailed in the reviewer responses below.

Response to reviewer suggestions are written in *blue*.

Reviewer 1:

Below my comments: 1-Throughout the manuscript authors interchangeably use the terms "nuclear deformations" and "nuclear blebs" as synonyms, which is not right and makes it difficult to interpret the data. Authors should explain/define the differences of each term and use only nuclear blebs (since this is their subject of study).

We appreciate the reviewers feedback and modifying the text throughout to clarify that blebs are a subset of nuclear deformations. We then rectified the text to indicate that the measurements are focused on blebs in line 71 with "Among the most radical deformations in nuclear shape is the protrusion of chromatin from the nuclear surface, known as a nuclear bleb, that are associated with pathological transformation. [2, 6, 15, 16]"

The nuclear bleb is not necessarily a chromatin protrusion. It can also be a result of a local nuclear envelope detachment from the underlying nuclear lamina devoid of chromatin (as the lamina might still be intact). Subsequently, upon nuclear lamina disruption/rupture the chromatin can then also gain access to the bleb (see ref https://doi.org/10.1016/j.ceb.2021.01.007)

We appreciate the reviewer's comment regarding this precise point. This is a commonly held perspective within the field that nuclear blebs are not chromatin protrusions. In our accompanying manuscript, Brunner et al, we experimentally demonstrate that the hallmark of blebs/protrusions is the protrusion of chromatin and not by the disruption of the lamina. As reported in the review above, this indicates that the widely held perspective in the field needs to be revisited as these deformations are defined by the chromatin state.

Thank you for the explanation as I did not know this paper. However, the cited paper does not address whether it has to occur a breach/rupture in the nuclear lamina in order to allow for chromatin to enter into the bleb. So, the question remains: Can a bleb be devoid of chromatin (as the lamina might still be intact and thus prevent chromatin from entering the bleb)? Or perhaps could the chromatin simply push the lamina into the bleb without breaking it? The fact the Lamin B1 is drastically different in blebs in comparison to the primary nucleus does not help us answering these questions which I must confess have been intriguing me for a long while!

This is a very interesting point. We have not observed nuclear blebs in the absence of chromatin, but have seen blebs with chromatin at lower density and collapsed structure.

2-In line 166 authors write: "…and associated with severely deformed nuclear periphery (Fig. 1B; Fig. S1A)". However, this data is not shown in these panels nor in the paper.

We appreciate the reviewer's careful review and apologize for this omission, we have corrected the manuscript as follows: Line 158-162: "Our results confirmed that GSK343 and TSA treatment significantly increased the percentage of blebbed nuclei within HCT-116 cells; with TSA treatment also inducing micronuclei formation and associated with a deformed nuclear periphery via increased number of blebs (Fig. 1A)."

Again, the revised data still does not show any image or quantification of micronuclei (!).

We apologize to the reviewer for omitting the quantification of the micro-nuclei from the re-submission. We have included it as a new row in supplementary table 1 which shows the average number of blebs and micronuclei across three replicates. For ease, we have attached table here as well for your reference.

SI Table 1: Average Number of Nuclei, Blebs and Micronuclei per FOV across three replicates

© 2025. Published by The Company of Biologists under the terms of the Creative Commons Attribution License (https://creativecommons.org/licenses/by/4.0/).

	Contr ol	DMSO	24 hr. Auxin	24 hr. GSK34 3	24 hr. Auxi n + GSK 343	24 hr. TSA	24 hr. Auxin + TSA
# of Nucelli	379.5 0	98.33	388.5 0	365.33	311.0 0	152.0 0	152.00
# Blebs	7.00	1.33	21.0 0	24.33	25.33	26.33	37.33
# Micro - nucle i	3	0.667	18.5	9.333	9.667	12.00 0	13.000

OK!

6-It's very hard to compare and interpret the data in the graphs in Figure 2 (one has to keep going back and forth between different graphs). Please put them all in the same graph.

We have consolidated the data into a single graph as requested. All quantitative measurements were placed in a graph with 3x3 subpanels for easier navigation.

There are no major changes made in revised Fig 2, in fact it is almost the same as in the first version (!), so it is still very confusing. And I see no single graph or 3x3 subpanels.

We apologize for the miscommunication in how we described the changes to figure 2. We reorganized the figures for clarity by repositioning all the quantitative parameters measured (D, FMM, Effective Diffusion coefficient) on one side of the figure in a ***3x2 organization****. We believe that this separation is clearer since the images for both treatment types: the auxin treatment in HCT116 LMNBB1&B2-AID cells and the GSK343, TSA treated cells are separated from the data. In reviewing the results, we find that separating the conditions makes it clearer and avoids crowded graphs.*

OK!

8-mClover signal is shown in figure 3 however no mention is made in the main text. Please explain and justify the use of mClover.

The use of mClover is a marker for the stable introduction of the cell line. We have modified the text to reflect that appropriately in lines 142-143. "This cell line has an mClover fluorescent reporter fused to the lamin B1/B2 proteins that was used in our degradation experiments."

What do the authors mean by "stable introduction of the cell line"? This makes no sense to me. Also, if the mClover is fused to the laminB1/2 proteins, why can't we see the LaminB1/2 signal (decorating the nuclear envelope/lamina) in the DMSO panel of Fig.3? (as it nicely shown in figs. 1 and 2 of the referenced paper by the authors: Depletion of lamins B1 and B2 promotes chromatin mobility and induces differential gene expression by a mesoscale-motion dependent mechanism, 2024).

Also, I do not know how to interpret the presence of the mClover signal and localization in the TSA treated sample. This is all very confusing to me and will certainly be to any reader.

The HCT116 cell line was genetically engineered to constitutively express mClover fluorescent protein fused to lamin B1/B2. This genetic modification ***was stably integrated*** *into the genome, creating a cell line that permanently expresses the fluorescent reporter across cell generations. Hence we replied to the first reviewer comments with mClover is a marker for the stable introduction of the AID system.*

OK, but please be more clear as "stable introduction of the cell line" is not a term used to

© 2025. Published by The Company of Biologists under the terms of the Creative Commons Attribution License (https://creativecommons.org/licenses/by/4.0/).

indicate the generation of a stable cell line. That's why I was confused.

We appreciated the reviewer's comment, we have updated the language to not confuse the readers to " this genetic modification was stably integrated into the genome"
We would like to clarify to the reviewer that the imaging modality used in this manuscript's figure 1, and the referred manuscript (Depletion of lamins B1 and B2 promotes chromatin mobility and induces differential gene expression by a mesoscale motion dependent mechanism, 2024) is different from that used in figure 3. Figure 1 and the reference use confocal imaging while the fluorescent images in figure 3 are taken on the PWS imaging system which uses a Liquid Crystal Tunable Filter (LCTF). This component performs the spectral discrimination needed by PWS. The LCTF has a narrow passband which causes a weaker SNR, as such the enriched borders seen in figure 1and in the referenced manuscript are not present here. We recognize that this is not clear and have updated the legend to reflect this difference.

OK, but can the authors please include to the figure legend the exact explanation above. I think this will be helpful to the readers.

We have clarified in each figure legend the imaging modality used to acquire the images/

Lastly, we would like to clarify to the reviewer that the cells in this figure were treated with TSA not auxin so there is no lamin degradation. The label on the images signifies that these are the stable AID cell lines used throughout the experiment. We clarified and emphasized in the legend to avoid reader confusion.
I got that the cells were not treated with auxin. I just did not know if there was some meaning/result/message being conveyed by the authors regarding the distribution of the mClover signal in this sample since there is virtually no signal in the DMSO sample.

We thank the reviewer for their suggestion.

9 – In fig.3A authors mention that temporal interference signal is inversely proportional to chromatin density. In line 242 authors mention that: "…within a TSA treated cell with a visually apparent bleb, chromatin decreases rapidly adjacent to the nuclear bleb…"
However, the frames show no apparent changes (let alone an increase) in the temporal interference signal (which would be expected if chromatin density were to decrease).
This observation was in reference to the accompanying video with still captures of the events. We have added arrows to indicate the locations we are referring to on the respective image.

1. It is still very hard for me to see the changes the authors are referring to (arrows are not helping).
2. Additionally, the authors did not address my concern regarding no apparent changes in the temporal interference signal shown in the panels (which if any, are VERY subtle). Maybe I just don't fully understand the technique/approach used so I'm not able to interpret these panels. If this is the case, I anticipate that other readers will have the same issue and a much better job in the main text AND in the figure panels must be made.
3. Also, the concept of CVC should be fully explained in the main text as well as its significance and meaning (the authors simply bring it up out of the blue in the main text without any explanation of what it means and why they are looking at this parameter: "When measuring the relative CVC change over time in individual cells, we see that there is a heterogeneity when looking at both nuclear body and bleb regions of interest (Fig. 3B))." Although mentioned in the Methods section, I could not find any reasonable explanation of the term. In fact, in the first version of the manuscript it was at least written that it means chromatin volume concentration (CVC) but now in the revised version this was fully omitted.

1. *We apologize to the reviewer if the changes are not clear in our last revision. Firstly, we would like to draw your attention to the cropped image below. The yellow arrow is supposed to point towards the region of the nucleus near the bleb which shows a change in the signal. We have selected the best cell and time to show case these differences.*

© 2025. Published by The Company of Biologists under the terms of the Creative Commons Attribution License (https://creativecommons.org/licenses/by/4.0/).

OK! I guess what caused confusion was the fact that I'm not used to "read" this type of data. The authors should be more didactic when mentioning this specific result in the main text.

We thank the reviewer for their suggestion.

2. *We apologize for the difficulty in interpreting these figures. To facilitate understanding, we have added two diagrams that visually show examples of two nuclear CVC's so readers can see how these changes are physically. These diagrams have been added as panels to figure 3.*
   Please add graph keys to both graphs in Fig 3B to indicate that blue and orange curves represent blebs and nuclear body respectively, just as it was done for panel 3C (actually authors should make the colors the same for graphs in figure 3B and 3C to be consistent).

   We apologize for not having this in the last submission. We have added the key for ease of interpretation for the reader.

3. *We apologize for the lack of explanation in the resubmission, we have added a brief description of CVC when it is first introduced in the results section for figure 3 and point the reader to the methods section. Please see the description that we have added below.*

   As discussed above, variations in the temporal interference quantifies the FMM while the temporal-average signal is inversely proportional to the chromatin volume concentration (chromatin density) and the density of other nuclear macromolecules.[39, 41]. Chromatin volume concentration (CVC), as the name implies, describes how chromatin occupies the volume on a relative scale of 0 to 1. Two examples of relative CVC that are seen in our analysis are illustrated in Fig. 3A

   OK!

10-In line 245 authors say: "…evidenced by increased local chromatin density and the visual changing of density in the bleb". I could not see or understand what the authors are referring to. Can the authors point more specifically to the referred data?

We thank the reviewer for their comment and have modified the text as follows to improve clarity in lines 265-268: "In the TSA treated case (Fig. 3A bottom), we observe active transit of small amounts of mass between the nuclear bleb and the main nuclear body. This is evidenced by an increased local chromatin density and the temporally evolving density in the bleb (Fig 3A TSA t:1/200-t:200/200)." We have also added arrows to the figure panel to indicate the area of interest with the referred change in density.

Again, I have the same issues as mentioned in my reply to the point above (point 9) - no apparent changes in the temporal interference signal can be observed in the panels (Fig. 3A, TSA t:1/200-t:200/200).

We appreciate that the differences may be visually subtle but the quantification for an example cell treated with TSA (Fig. 3C) and ensemble averages (Fig. 3D) are shown.
OK! And again: I guess what caused confusion was the fact that I'm not used to "read" this type of data-despite having a deep knowledge on microscopy techniques. Thus, the authors should be more didactic when mentioning this specific result in the main text for a broader audience.

We thank the reviewer for the suggestions.
12-In line 277 authors write: "we found that the nuclear blebs arising from either B type lamin degradation or TSA treatment contained heterochromatin around the periphery of blebs and within the center of the bleb (Fig. 4C, E, F; Fig. S2)".

1. However, the figure panels they are referring to do not match what they are saying AND there is no heterochromatin enrichment in any bleb region of TSA treated U2OS cells.

© 2025. Published by The Company of Biologists under the terms of the Creative Commons Attribution License (https://creativecommons.org/licenses/by/4.0/).

2. Also, why there is no "Nuclear bleb" and "Nuclear body" for the Control U2OS cells in panel 4F (but there is "Nuclear bleb" and "Nuclear body" for the Control HCT116 cells in fig. S2)? Where are the graphs for auxin and GSK343 treatments for U2OS cells?
3. The scales in the graphs shown in panel 4F are different making it difficult to interpret the results. This entire figure needs a major revision to be interpretable.

I see no arrows in the revised figure.

We apologize to the reviewer for the oversight. The arrows have now been incorporated into figure 4.

OK!

13-In line 281 authors say: "HDAC inhibition gives rise to non-canonical nuclear blebs enriched in heterochromatin around near their boundaries and the transfer of nanoscopic heterochromatin domains into the bleb". However, the data shows no heterochromatin enrichment in the bleb and it is unclear what the authors meant by "transfer of nanoscopic heterochromatin domains into the bleb" (where is the data for that?).

We thank the reviewer for this comment. The stained molecule is constitutive heterochromatin, H3K9me3, which is clearly visualized within the nuclear bleb. SMLM utilizes a time acquisition of blinking fluorophores (~10,000 frames, 30 ms exposure). This data is used to reconstruct the images shown in this figure with brighter pixels representing the presence of many localizations within that area. The inset for Fig. 5B with the added arrow shows bright areas in the bleb near the periphery representing the enrichment for heterochromatin we are referring to for the TSA treatment case.

Again, I see no arrows in the revised figure. Critically, there is no enrichment of heterochromatin in the bleb as the authors claim-the graphs clearly show this (the counts in the nuclear body are orders of magnitude higher than in the bleb). Rather, there is presence of heterochromatin in the blebs (the authors even underline this word in point 1 above).

We apologize to the reviewer for this oversight. We have added the arrows that were intended to show the presence of heterochromatin (yellow signal) in the nuclear bleb. We agree that the use of enrichment in our first response is misleading. We have ensured that there is no mention of enrichment in the main manuscript and only highlight the ***presence*** *of heterochromatin in the nuclear bleb. Our use of enrichment was in reference to the bright borders of the nuclear envelope in the bleb but appreciate the reviewer for expressing the confusion.*

Although the authors claim that they added the arrows-I still cannot see any arrows (!)unless I downloaded the wrong updated figure. Of note, the same happened in the second round of revisions (when authors claimed they had added arrows in fig. 4 and micronuclei quantification to fig. 1, but nothing was added-only in this latest version after I again pointed that these were missing, they were finally added). In my opinion, this shows that the authors are not being careful enough when revising their manuscript as this is already the 3 round of revisions and the same problem persists.

We apologize to the reviewer if the version they have downloaded did not have the arrow, but the submitted version for the latest round of revision of has the arrow that seems to be missing. We have double checked the original adobe illustrator file, the pdf and the portal version and they all contain this arrow. We hope the system error is fixed so this does not happen again.

The quantification in this figure is not a general measure of heterochromatin, instead it is quantifying the number of heterochromatic clusters that have been identified by our analysis algorithm. The nuclear body and bleb are not expected to share the same magnitude, as the body contains a greater proportion of the genome. The body and bleb should not be compared to each other, rather they should be considered separately and compared across treatments. We reviewed the figure legend and the manuscript to make sure this is clear.

OK!

© 2025. Published by The Company of Biologists under the terms of the Creative Commons Attribution License (https://creativecommons.org/licenses/by/4.0/).

16-All the observed changes in panels 1E, F are very minimal, so one could wonder how relevant these changes are. Authors should perform WB analysis of the histone markers

We have previously demonstrated that these histone marks are changed with WB analysis in the cell population. Western blot analysis for these treatments have been done in https://doi.org/10.1091/mbc.E17-06-0410 which corroborate our results. Further, these agents are widely used for this exact intended purpose.

Ok for WB but still, most IF quantifications shown in this manuscript show very discrete/minimal changes so it makes it difficult to assess their real contribution to the phenotypes described.

We appreciate the reviewer's comment on the IF quantification. In figure 1, we show plots C and D which demonstrate a change in the distribution of corrected total fluorescent signal of H3K27me3 and H3K27ac respectively for different conditions. As stated in the manuscript, these changes were statistically tested via a One-way ANOVA, and multiple comparisons were done via Dunnett's test. Within the main text, we only note the statistically different groups thus only highlighting the treatments that were contributing to phenotypes we are describing.

OK!

Reviewer 3:

The authors have made thorough revisions to the manuscript and adequately addressed all comments and recommendations provided earlier. One minor comment: "An equivalent final concentration of DMSO in media was used for the control group." The authors should rephrase this statement and mention the exact amount of DMSO added in volume/volume units(v/v). Given that DMSO can influence cell viability and experimental outcomes even at low concentrations, providing precise details is critical for reproducibility and for evaluating whether the DMSO levels used are within a non-toxic range. After addressing this minor comment, the manuscript is suitable for publication.

We apologize to the reviewer for omitting this in the last submission. We have added % v/v values for DMSO in the method section for the drug treatments.

© 2025. Published by The Company of Biologists under the terms of the Creative Commons Attribution License (https://creativecommons.org/licenses/by/4.0/).
